# Supplementary material for: Single-cell spatial metabolomics with cell-type specific protein profiling for tissue systems biology
Source: Nat Commun. 2023 Dec 13;14:8260. doi: 10.1038/s41467-023-43917-5 (PMC10716522; doi:10.1038/s41467-023-43917-5)
Supplement: Supplementary file 1 — SUPPLEMENTARY INFORMATION [file 41467_2023_43917_MOESM1_ESM.pdf]

Supplementary Information for

Single-cell spatial metabolomics with cell-type specific protein profiling for tissue systems biology

Thomas Hu[1,2,#], Mayar Allam[1,#], Shuangyi Cai[1], Walter Henderson[3], Brian Yueh[4], Aybuke Garipcan[4], Anton V. Ievlev[5], Maryam Afkarian[6], Semir Beyaz[4], and Ahmet F. Coskun[1,7,8,9*]

[1] Wallace H. Coulter Department of Biomedical Engineering, Georgia Institute of Technology and Emory University, Atlanta, GA, USA

[2] School of Electrical and Computer Engineering, Georgia Institute of Technology, Atlanta, GA, USA

[3] Institute for Electronics and Nanotechnology, Georgia Institute of Technology, Atlanta, GA, USA

[4] Cold Spring Harbor Laboratory, Cold Spring Harbor, NY, USA

[5] Oak Ridge National Laboratory, Center for Nanophase Materials Sciences, Oak Ridge, TN, USA

[6] Division of Nephrology, Department of Internal Medicine, University of California, Davis, CA, USA

[7] Interdisciplinary Bioengineering Graduate Program, Georgia Institute of Technology, Atlanta, GA, USA

[8] Winship Cancer Institute, Emory University, GA, USA

[9] Parker H. Petit Institute for Bioengineering and Bioscience, Georgia Institute of Technology, Atlanta, GA, USA

[#]Equal authorship *Corresponding author

Ahmet F. Coskun, Ph.D. (ahmet.coskun@bme.gatech.edu)

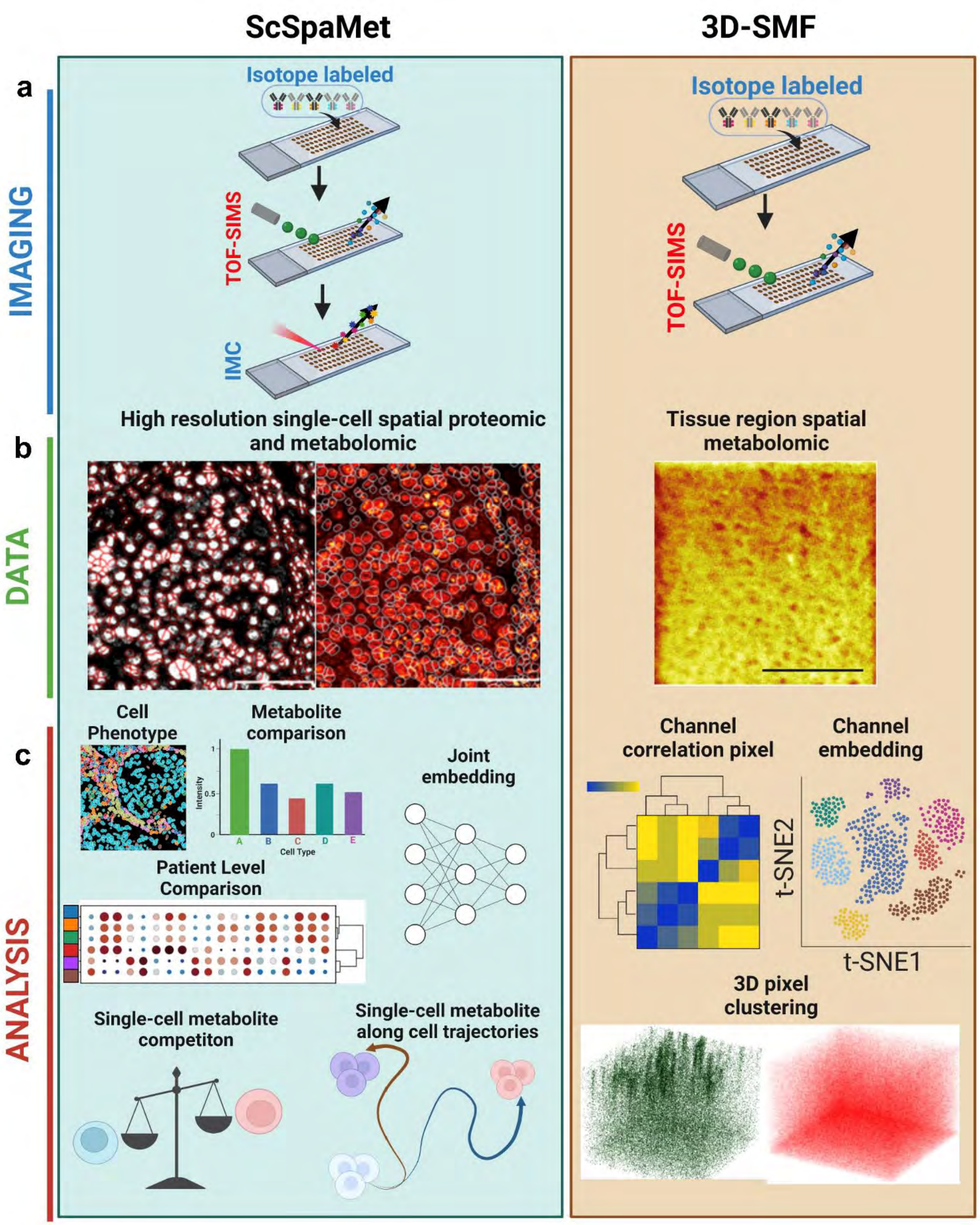

ScSpaMet
3D-SMF
a
IMAGING
Isotope labeled
TOF-SIMS
IMC
Isotope labeled
TOF-SIMS
b
DATA
High resolution single-cell spatial proteomic and metabolomic
Tissue region spatial metabolomic
c
ANALYSIS
Cell Phenotype
Metabolite comparison
Intensity
Cell Type
A
B
C
D
E
Joint embedding
Patient Level Comparison
Single-cell metabolite competiton
Single-cell metabolite along cell trajectories
Channel correlation pixel
Channel embedding
t-SNE2
t-SNE1
3D pixel clustering

Supplementary Fig. 1. Comparison of scSpaMet and 3D-SMF imaging and analysis pipeline.

a Comparison of scSpaMet and 3D-SMF imaging pipeline. ScSpaMet advanced the 3D-SMF imaging pipeline by introducing sequential TOF-SIMS and IMC imaging achieving high-resolution single-cell spatial proteomic and metabolomic. Created with Biorender.com

b Comparison of scSpaMet and 3D-SMF data output. ScSpaMet achieved high-resolution single-cell imaging and cell segmentation for joint proteomic and metabolomic downstream analysis. 3D-SMF revealed tissue region-specific metabolomic info.

c Comparison of scSpaMet and 3D-SMF data analysis. ScSpaMet analyzed single-cell phenotype from proteomic data and compared patient and cell type metabolite variation. ScSpaMet introduced muti-omics cell competition, multi-modal data integration, and multi-omics trajectory inference. The 3D-SMF only analyzed metabolite channel correlation from pixel data, channel embedding, and 3D pixel clustering due to a lack of single-cell information. Created with Biorender.com

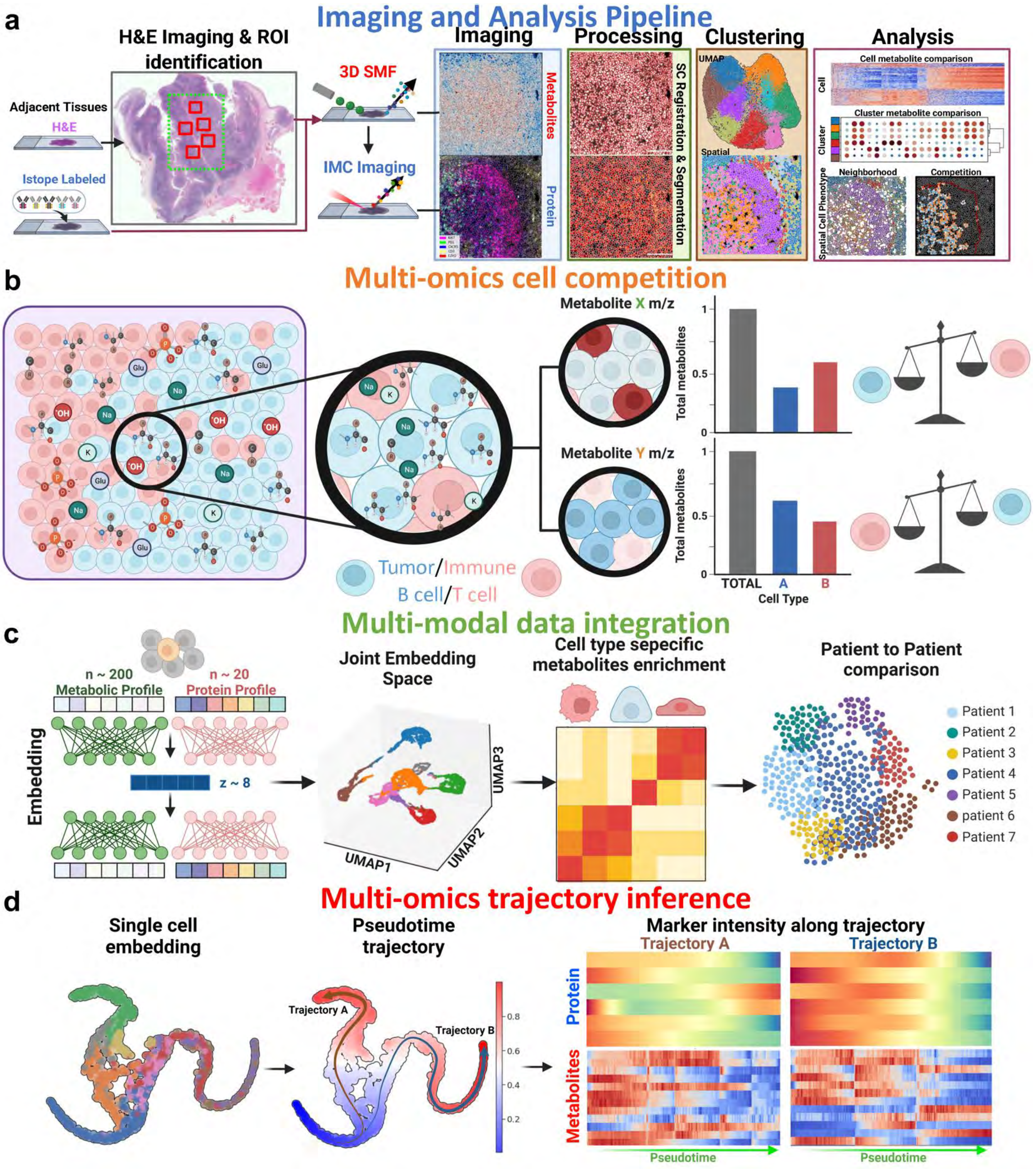
a
Imaging and Analysis Pipeline
H&E Imaging & ROI identification
Adjacent Tissues
H&E
Istope Labeled
3D SMF
IMC Imaging
Imaging
Metabolites
Protein
Processing
SC Registration & Segmentation
Clustering
UMAP
Spatial
Analysis
Cell metabolite comparison
Cell
Cluster metabolite comparison
Cluster
Spatial Cell Phenotype
Neighborhood
Competition
b
Multi-omics cell competition
Metabolite X m/z
Metabolite Y m/z
Total metabolites
Glu
Na
K
OH
Tumor/Immune
B cell/T cell
TOTAL
A
B
Cell Type
c
Multi-modal data integration
n ~ 200
Metabolic Profile
n ~ 20
Protein Profile
Embedding
z ~ 8
Joint Embedding Space
UMAP1
UMAP2
UMAP3
Cell type sepecific metabolites enrichment
Patient to Patient comparison
Patient 1
Patient 2
Patient 3
Patient 4
Patient 5
patient 6
Patient 7
d
Multi-omics trajectory inference
Single cell embedding
Pseudotime trajectory
Trajectory A
Trajectory B
Marker intensity along trajectory
Trajectory A
Trajectory B
Protein
Metabolites
Pseudotime
Pseudotime

Supplementary Fig. 2. ScSpaMet analysis modules.

a Overview of scSpaMet data analysis pipeline. Tissue samples on glass slides are labeled with metal-isotope conjugated antibodies and adjacent tissues stained with H&E are used for the identification of imaging regions for scSpaMet. This is followed by metabolic profiling with 3D-SMF and proteomic profiling using IMC. After imaging, single-cell registration is performed with downstream analysis. Created with Biorender.com

b Overview of ScSpaMet local metabolomic competition analysis pipeline. Single cells compete with their neighboring cell for metabolomic resources. ScSpaMet quantifies neighboring cell types' local metabolite competition. Created with Biorender.com

c Overview of ScSpaMet single cell joint modality VAE embedding pipeline. Single-cell metabolomic and proteomic profiles are used as input for the VAE to extract joint embedding space representation. Joint embedding is used to decipher cell type metabolic states and enrichment as well as stratify patients. Created with Biorender.com

d Overview of ScSpaMet pseudo time analysis pipeline. Using single-cell embedding, cell trajectory differentiation pseudotime analysis is used to measure metabolomic and proteomic change along trajectories. Created with Biorender.com

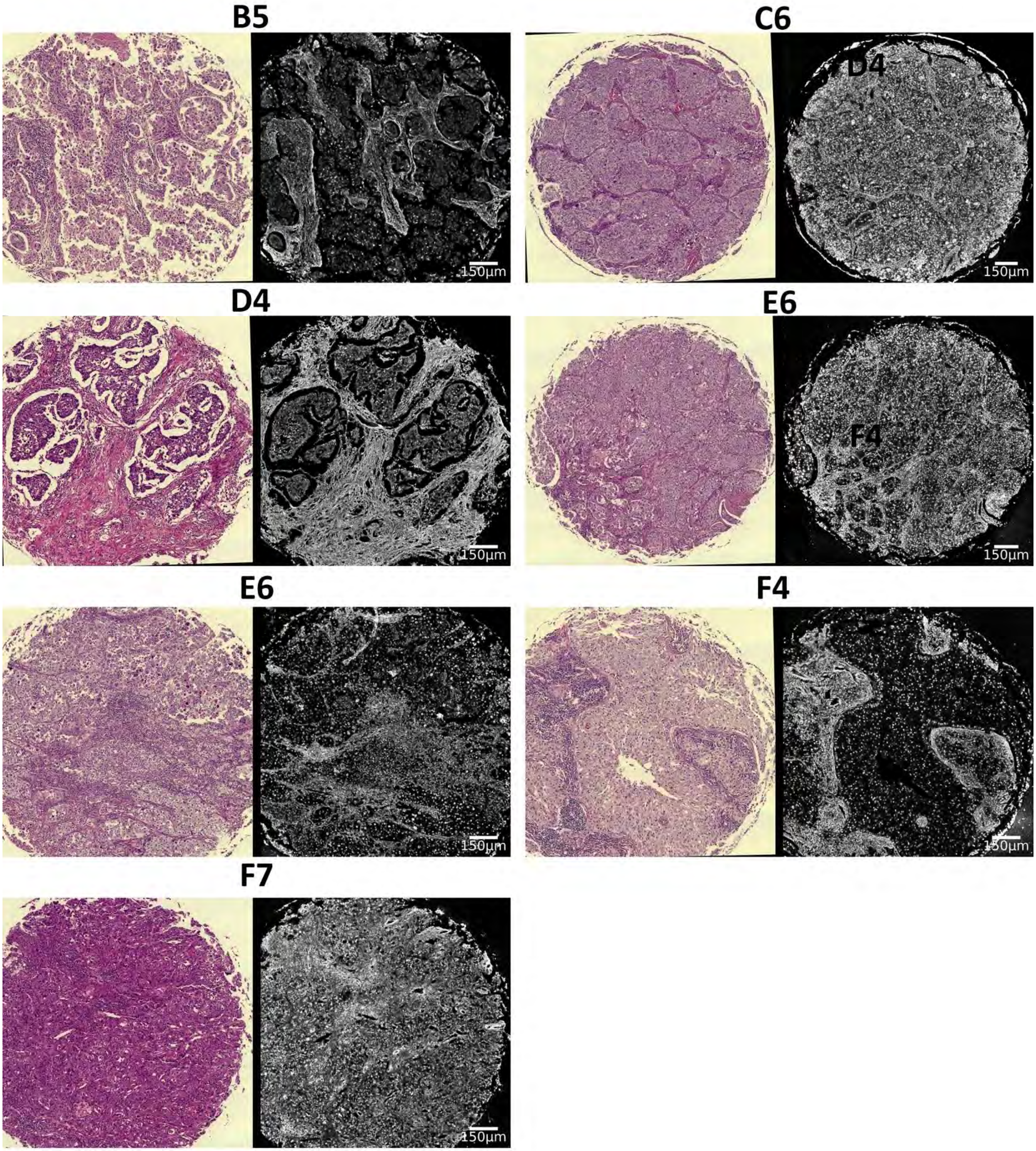
B5
C6
D4
D4
E6
F4
E6
F4
F7
150µm

Supplementary Fig. 3. Matched H&E and IMC Histone H3 marker images in human lung cancer tissues. For each imaged region, the registered H&E stained sequential tissues (left) are shown with the IMC Histone H3 marker images (right). Scale bar 150 μm.

TONSIL DONOR A

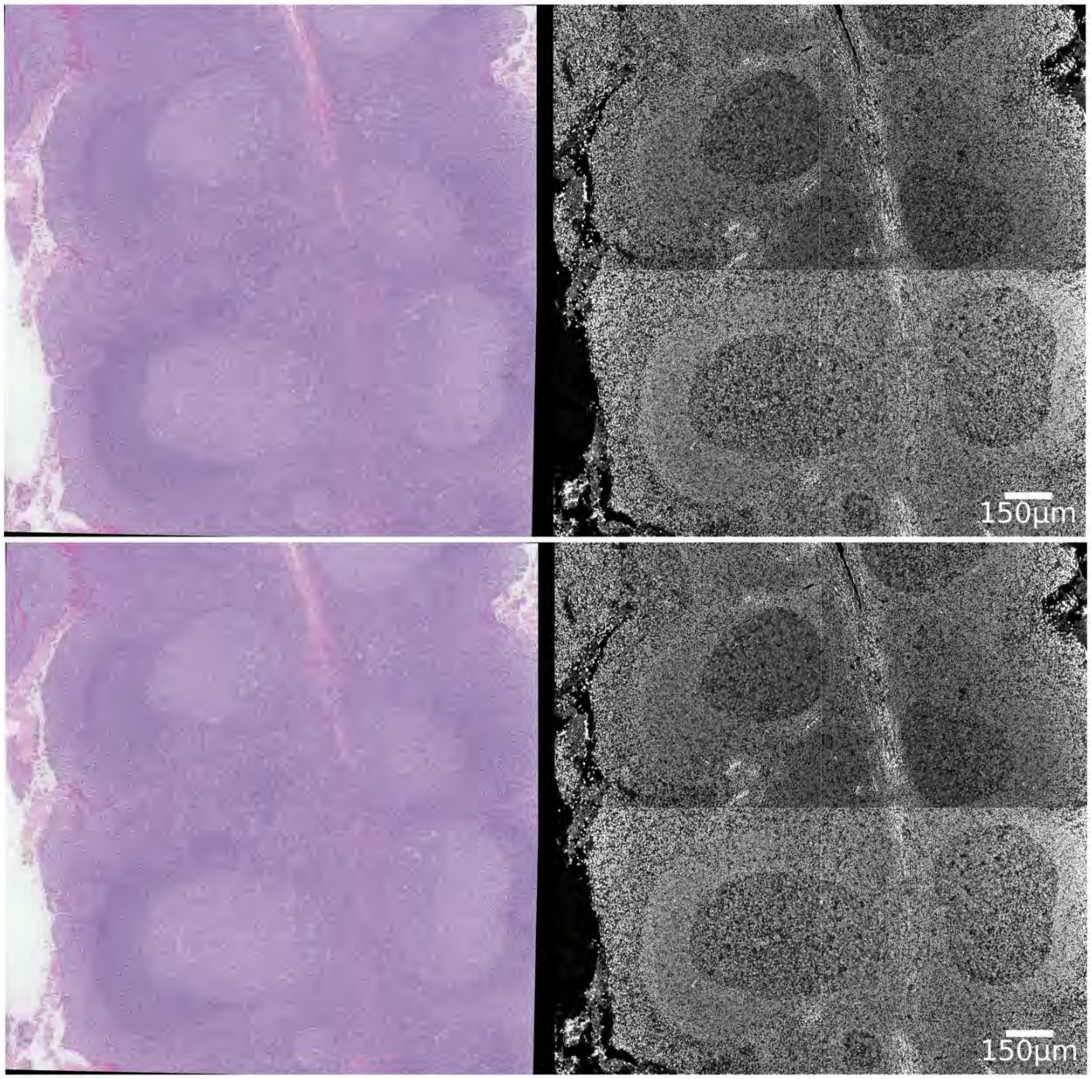

TONSIL DONOR E

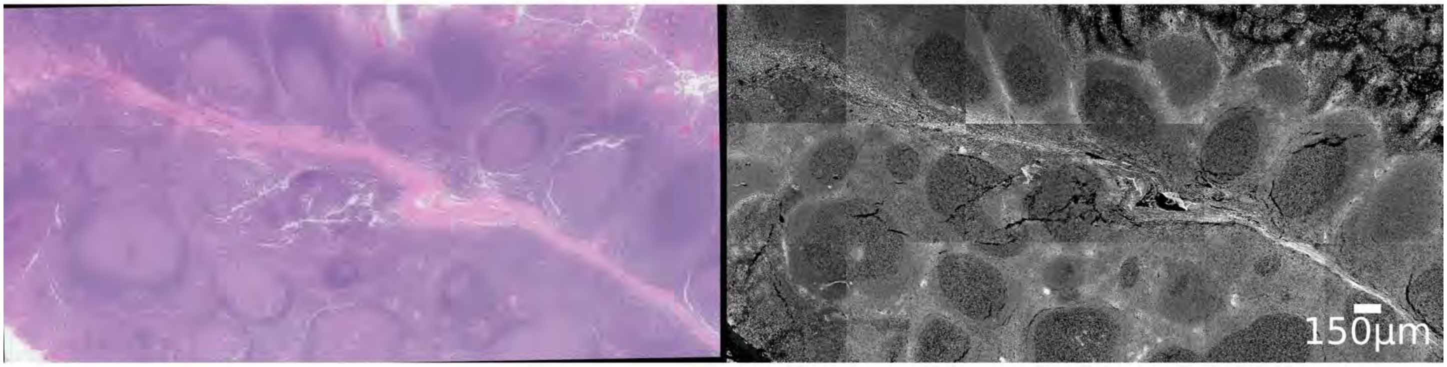

Supplementary Fig. 4. Matched H&E and IMC Intercalator marker images in human tonsil tissues. For each imaged region, the registered H&E stained sequential tissues (left) are shown with the IMC Histone H3 marker images (right). Scale bar 150 μm.

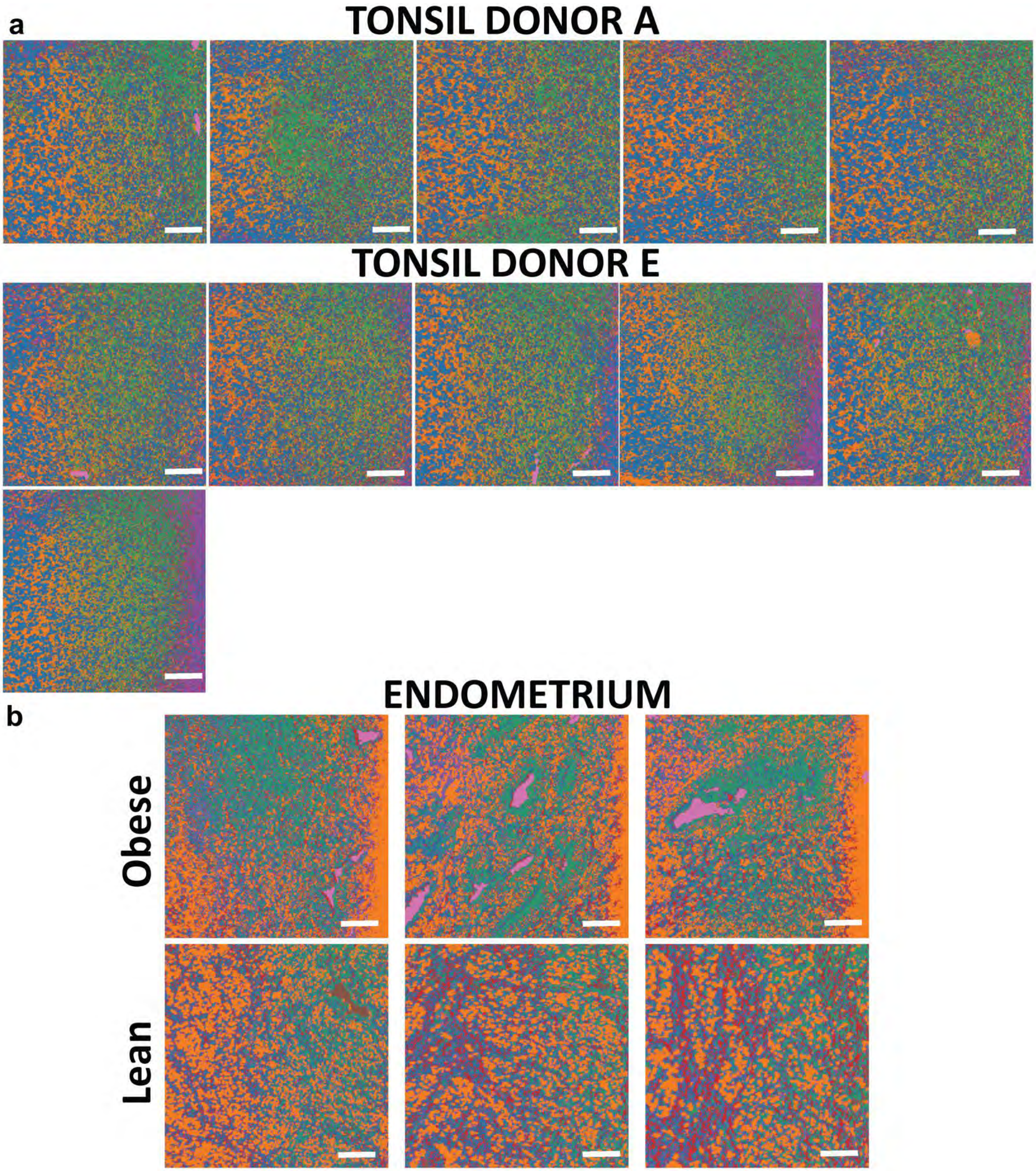
a
TONSIL DONOR A
TONSIL DONOR E
b
ENDOMETRIUM
Obese
Lean

Supplementary Fig. 5. Pixel clustering for SIMS data in tonsil tissues **a** and endometrium tissues **b**. Scale bar 100 μm.

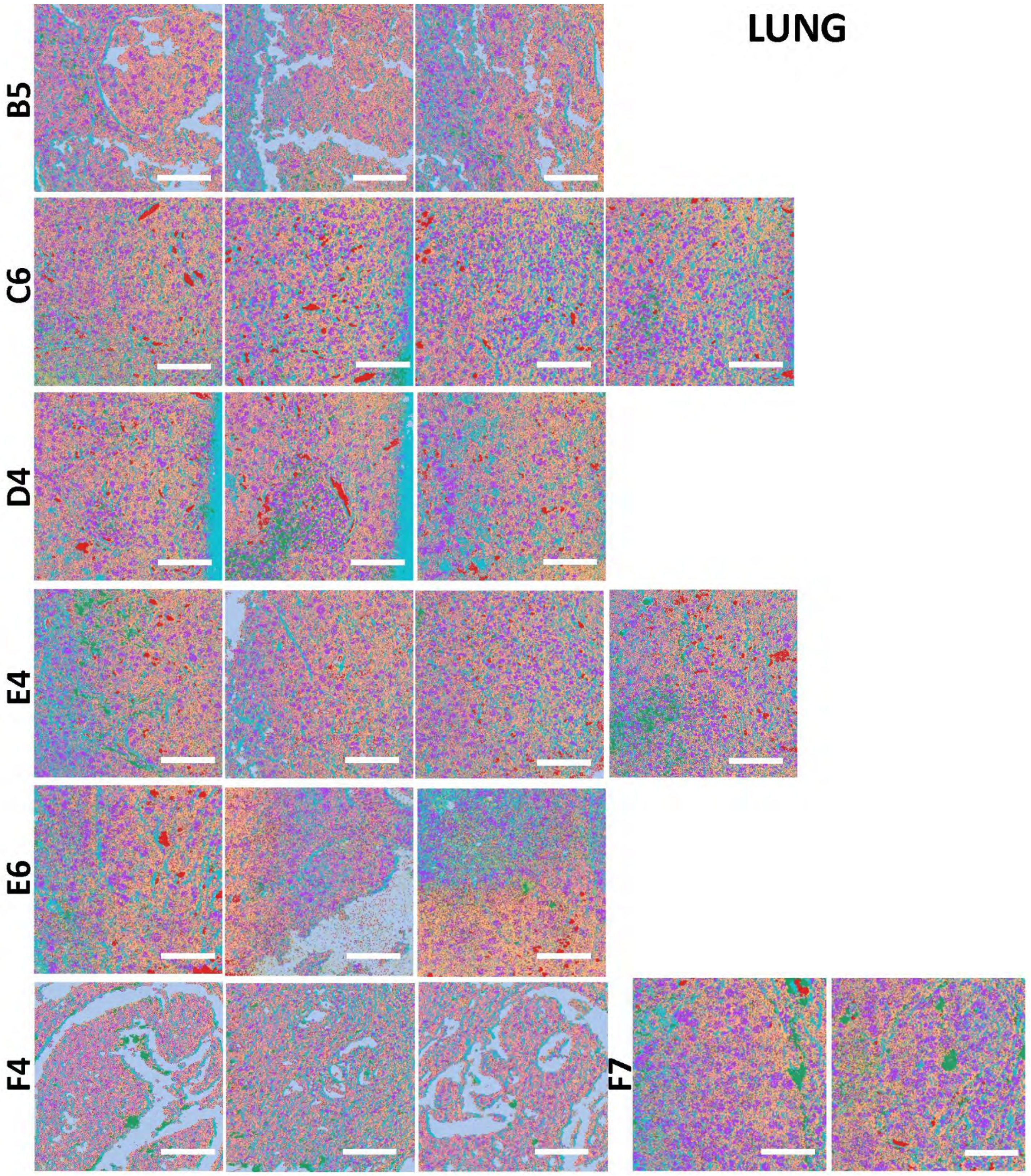
LUNG
B5
C6
D4
E4
E6
F4
F7

Supplementary Fig. 6. Pixel clustering for SIMS data in lung cancer tissues. Scale bar 100 μm.

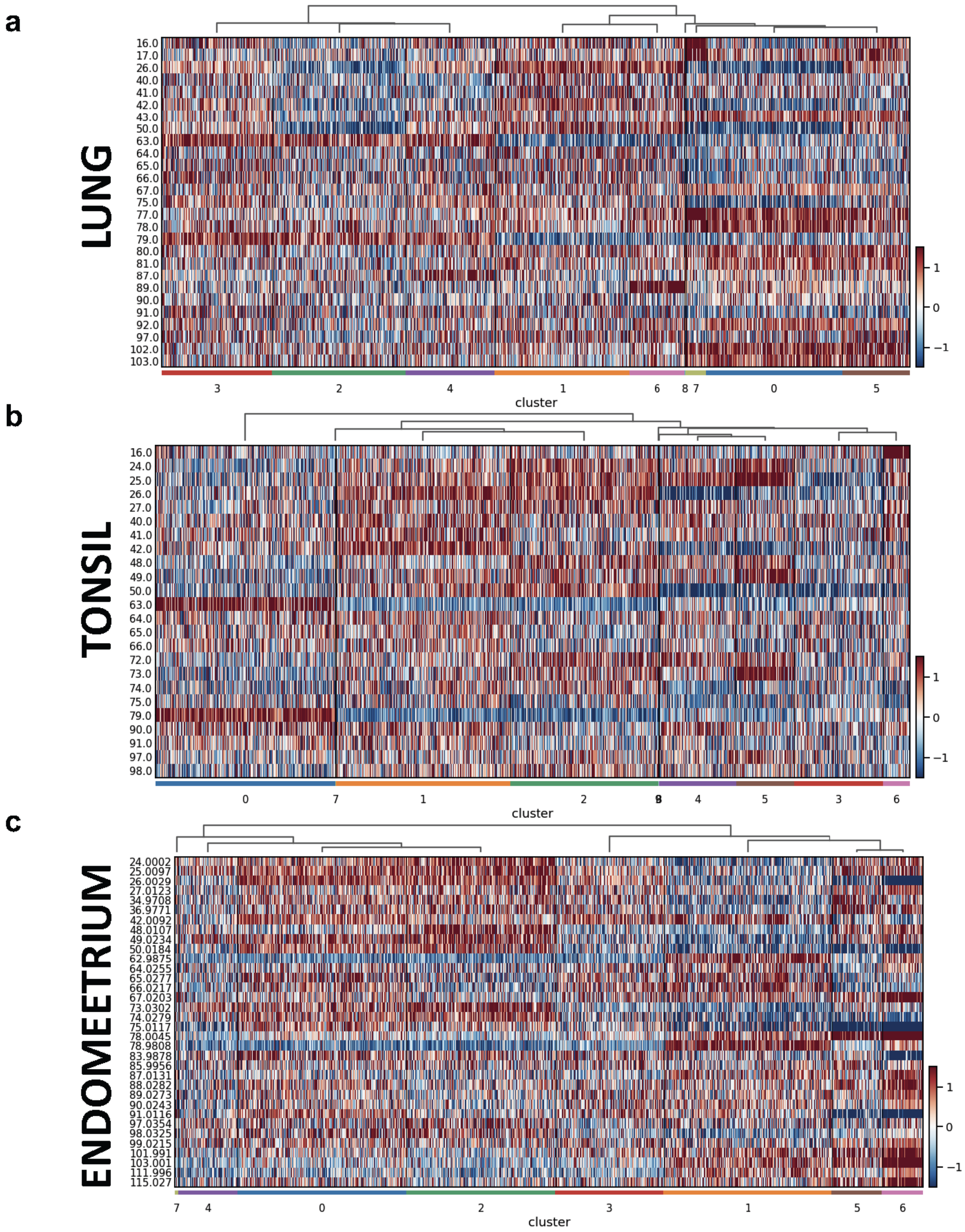
a
LUNG
16.0
17.0
26.0
40.0
41.0
42.0
43.0
50.0
63.0
64.0
65.0
66.0
67.0
75.0
77.0
78.0
79.0
80.0
81.0
87.0
89.0
90.0
91.0
92.0
97.0
102.0
103.0
3 2 4 1 6 8 7 0 5
cluster
1
0
−1
b
TONSIL
16.0
24.0
25.0
26.0
27.0
40.0
41.0
42.0
48.0
49.0
50.0
63.0
64.0
65.0
66.0
72.0
73.0
74.0
75.0
79.0
90.0
91.0
97.0
98.0
0 7 1 2 8 4 5 3 6
cluster
1
0
−1
c
ENDOMEETRIUM
24.0002
25.0097
26.0029
27.0123
34.9708
36.9771
42.0092
48.0107
49.0234
50.0184
62.9875
64.0255
65.0277
66.0217
67.0203
73.0302
74.0279
75.0117
78.0045
78.9808
83.9878
85.9956
87.0131
88.0282
89.0273
90.0243
91.0116
97.0354
98.0325
99.0215
101.991
103.001
111.996
115.027
7 4 0 2 3 1 5 6
cluster
1
0
−1

Supplementary Fig. 7. Pixel cluster expression for SIMS data for lung (**a**), tonsil (**b**), and endometrium (**c**). Heatmap showing the pixel expression level for selected mass channels. Colormap corresponds to Supplementary Fig. 3-4.

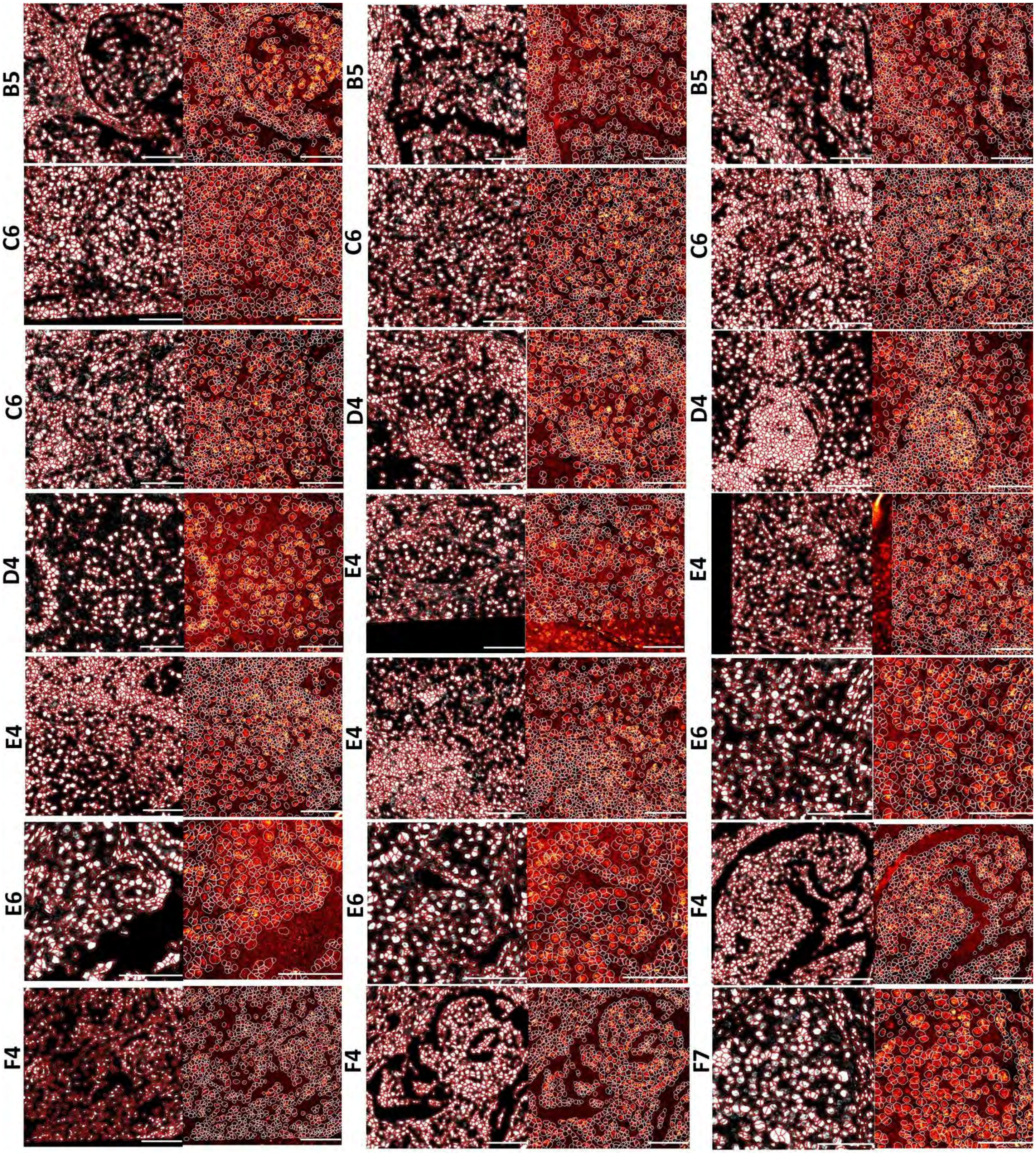
B5
C6
C6
D4
E4
E6
F4
B5
C6
D4
E4
E4
E6
F4
B5
C6
D4
E4
E6
F4
F7

Supplementary Fig. 8. Single-cell registration results between IMC Histone H3 marker (left) and SIMS PO3- channel (right) in human lung cancer tissues. Scale bar 100 μm.

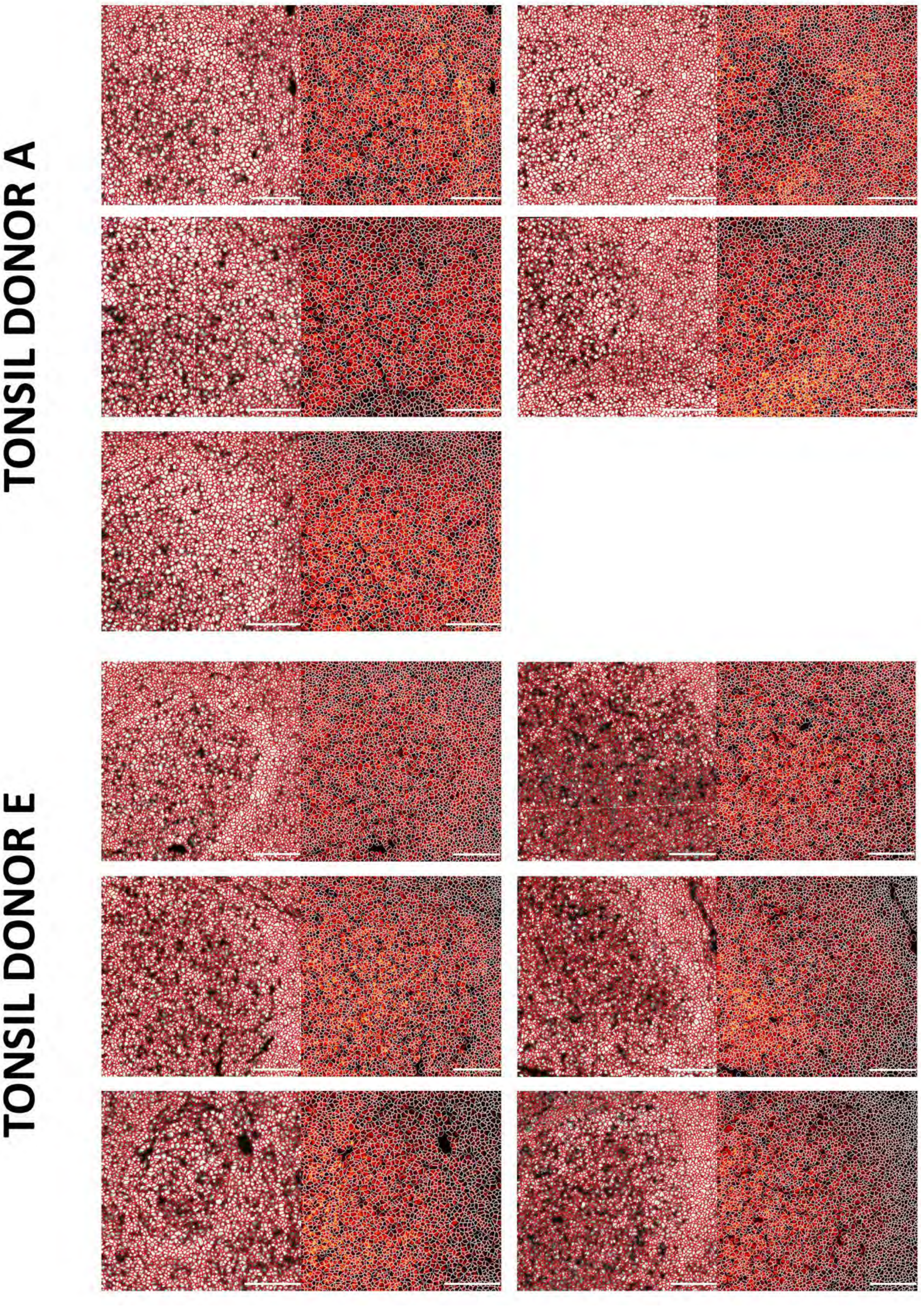
TONSIL DONOR A
TONSIL DONOR E

Supplementary Fig. 9. Single-cell registration results between IMC Intercalator marker (left) and SIMS PO3- channel (right) in human tonsil tissues. Scale bar 100 μm.

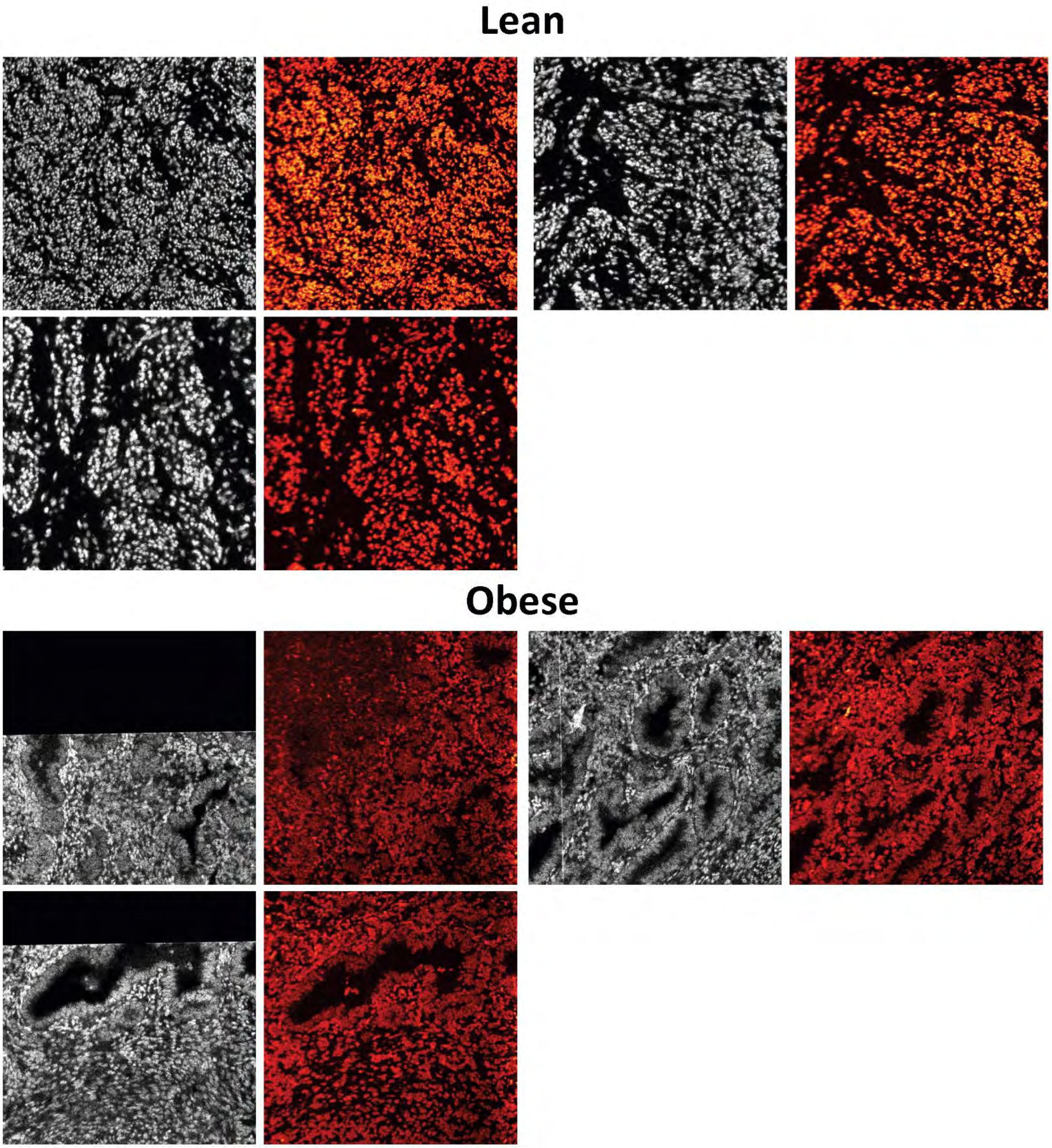
Lean
Obese

Supplementary Fig. 10. Single-cell registration results between IMC Intercalator marker (left) and SIMS PO3- (right) in human endometrium issues. Scale bar 100 μm.

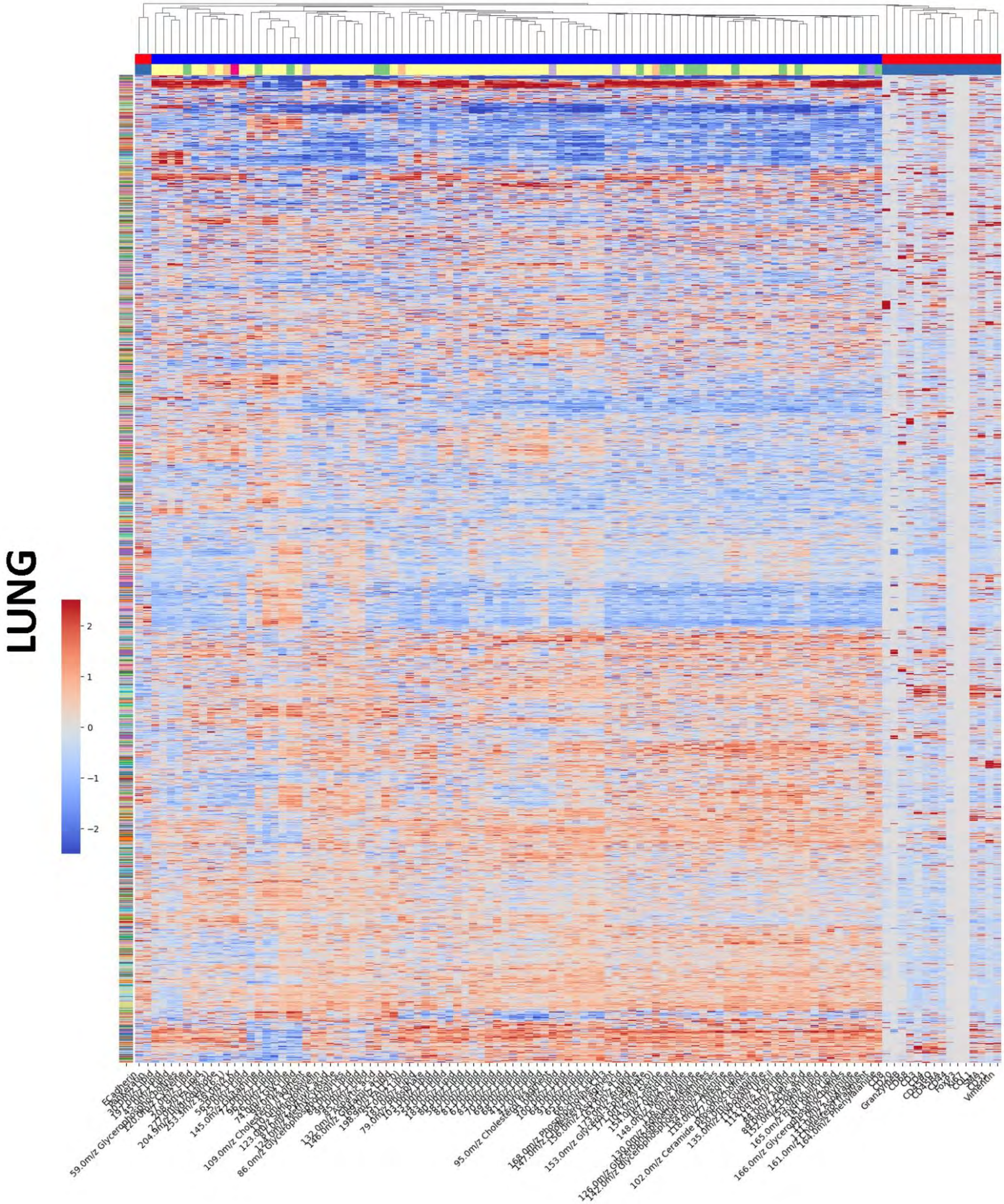
LUNG
2
1
0
−1
−2

Supplementary Fig. 11. Single-cell metabolite and protein profile in lung cancer tissues (n=19507 cells). Cluster map showing the cell-level metabolite and protein profile in all lung cancer tissues. Colorbar corresponds to the mean intensity value at the single cell level.

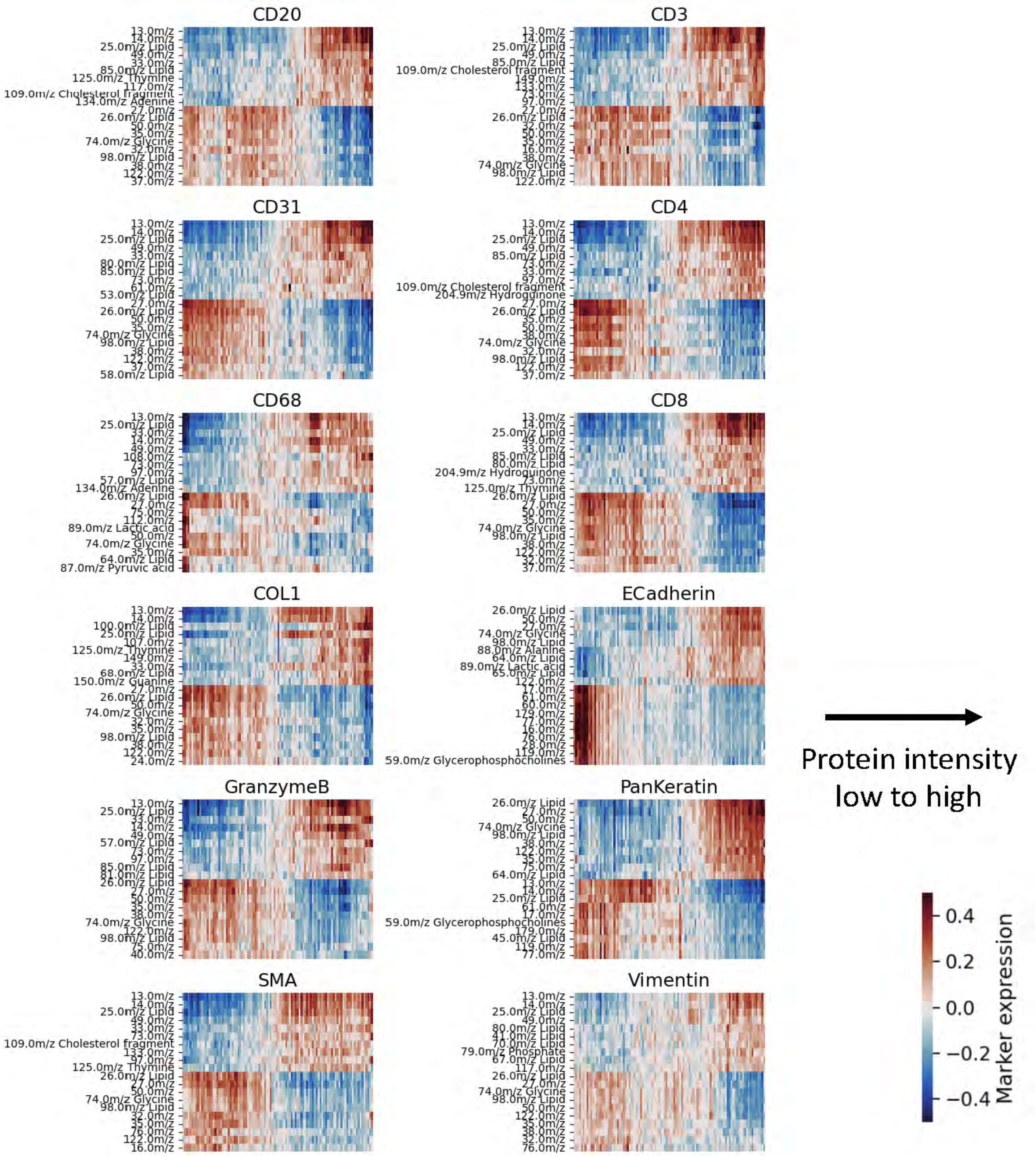
CD20
CD3
CD31
CD4
CD68
CD8
COL1
ECadherin
GranzymeB
PanKeratin
SMA
Vimentin
13.0m/z
14.0m/z
25.0m/z Lipid
49.0m/z
33.0m/z
85.0m/z Lipid
125.0m/z Thymine
117.0m/z
109.0m/z Cholesterol fragment
134.0m/z Adenine
27.0m/z
26.0m/z Lipid
50.0m/z
35.0m/z
74.0m/z Glycine
32.0m/z
98.0m/z Lipid
38.0m/z
122.0m/z
37.0m/z
149.0m/z
133.0m/z
73.0m/z
97.0m/z
16.0m/z
80.0m/z Lipid
61.0m/z
53.0m/z Lipid
58.0m/z Lipid
204.9m/z Hydroquinone
108.0m/z
57.0m/z Lipid
75.0m/z
112.0m/z
89.0m/z Lactic acid
64.0m/z Lipid
87.0m/z Pyruvic acid
100.0m/z Lipid
107.0m/z
68.0m/z Lipid
150.0m/z Guanine
24.0m/z
88.0m/z Alanine
65.0m/z Lipid
17.0m/z
60.0m/z
179.0m/z
77.0m/z
76.0m/z
28.0m/z
119.0m/z
59.0m/z Glycerophosphocholines
81.0m/z Lipid
40.0m/z
45.0m/z Lipid
41.0m/z Lipid
70.0m/z Lipid
79.0m/z Phosphate
67.0m/z Lipid
Protein intensity
low to high
0.4
0.2
0.0
−0.2
−0.4
Marker expression

101

Supplementary Fig. 12. Single-cell metabolite and protein profile correlation in lung cancer tissues. Each heatmap shows the correlation of metabolite channels with corresponding protein markers (n=19507 cells). Left to right shows ascending single-cell protein intensity expression.

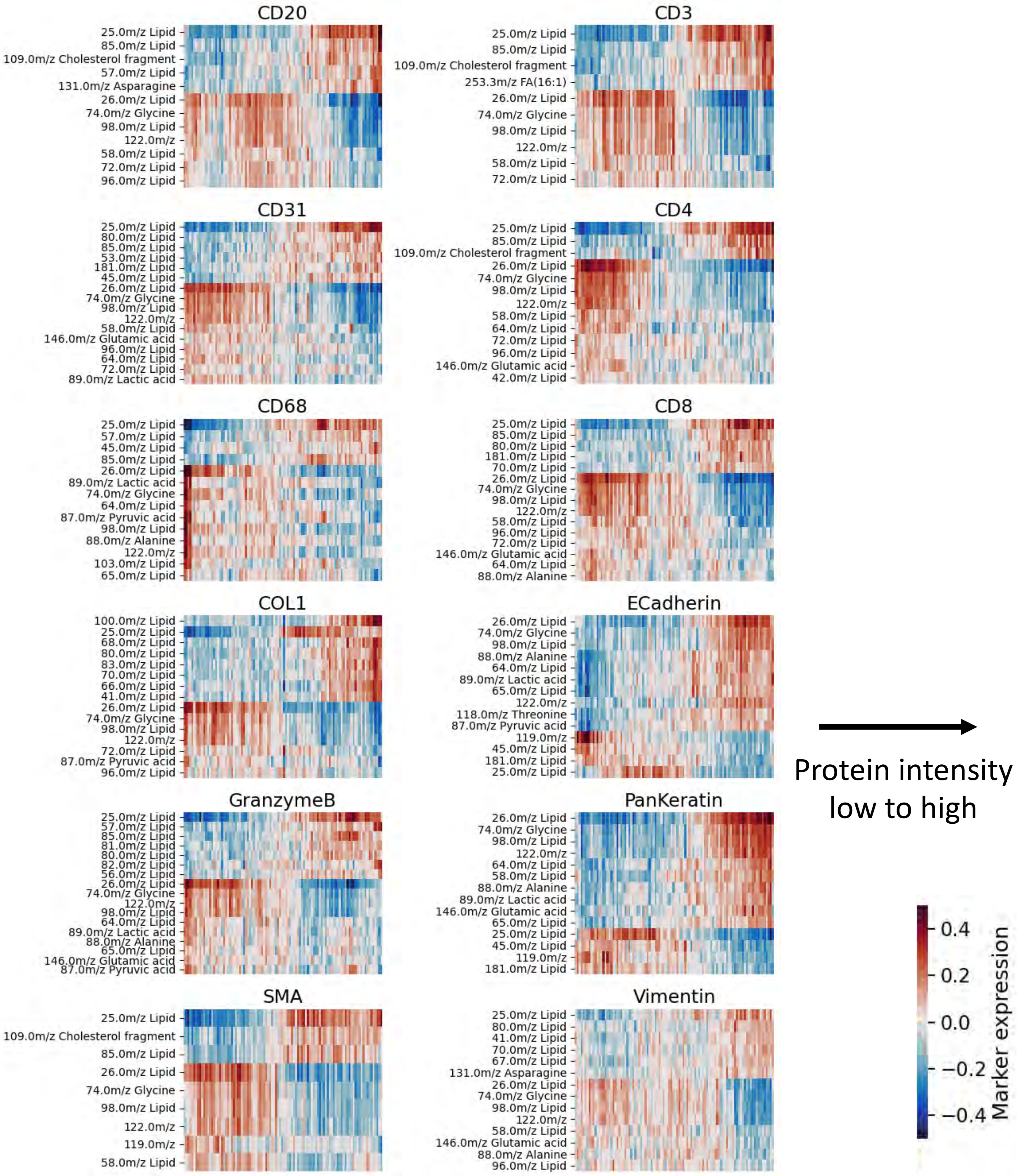
CD20
25.0m/z Lipid
85.0m/z Lipid
109.0m/z Cholesterol fragment
57.0m/z Lipid
131.0m/z Asparagine
26.0m/z Lipid
74.0m/z Glycine
98.0m/z Lipid
122.0m/z
58.0m/z Lipid
72.0m/z Lipid
96.0m/z Lipid
CD3
25.0m/z Lipid
85.0m/z Lipid
109.0m/z Cholesterol fragment
253.3m/z FA(16:1)
26.0m/z Lipid
74.0m/z Glycine
98.0m/z Lipid
122.0m/z
58.0m/z Lipid
72.0m/z Lipid
CD31
25.0m/z Lipid
80.0m/z Lipid
85.0m/z Lipid
53.0m/z Lipid
181.0m/z Lipid
45.0m/z Lipid
26.0m/z Lipid
74.0m/z Glycine
98.0m/z Lipid
122.0m/z
58.0m/z Lipid
146.0m/z Glutamic acid
96.0m/z Lipid
64.0m/z Lipid
72.0m/z Lipid
89.0m/z Lactic acid
CD4
25.0m/z Lipid
85.0m/z Lipid
109.0m/z Cholesterol fragment
26.0m/z Lipid
74.0m/z Glycine
98.0m/z Lipid
122.0m/z
58.0m/z Lipid
64.0m/z Lipid
72.0m/z Lipid
96.0m/z Lipid
146.0m/z Glutamic acid
42.0m/z Lipid
CD68
25.0m/z Lipid
57.0m/z Lipid
45.0m/z Lipid
85.0m/z Lipid
26.0m/z Lipid
89.0m/z Lactic acid
74.0m/z Glycine
64.0m/z Lipid
87.0m/z Pyruvic acid
98.0m/z Lipid
88.0m/z Alanine
122.0m/z
103.0m/z Lipid
65.0m/z Lipid
CD8
25.0m/z Lipid
85.0m/z Lipid
80.0m/z Lipid
181.0m/z Lipid
70.0m/z Lipid
26.0m/z Lipid
74.0m/z Glycine
98.0m/z Lipid
122.0m/z
58.0m/z Lipid
96.0m/z Lipid
72.0m/z Lipid
146.0m/z Glutamic acid
64.0m/z Lipid
88.0m/z Alanine
COL1
100.0m/z Lipid
25.0m/z Lipid
68.0m/z Lipid
80.0m/z Lipid
83.0m/z Lipid
70.0m/z Lipid
66.0m/z Lipid
41.0m/z Lipid
26.0m/z Lipid
74.0m/z Glycine
98.0m/z Lipid
122.0m/z
72.0m/z Lipid
87.0m/z Pyruvic acid
96.0m/z Lipid
ECadherin
26.0m/z Lipid
74.0m/z Glycine
98.0m/z Lipid
88.0m/z Alanine
64.0m/z Lipid
89.0m/z Lactic acid
65.0m/z Lipid
122.0m/z
118.0m/z Threonine
87.0m/z Pyruvic acid
119.0m/z
45.0m/z Lipid
181.0m/z Lipid
25.0m/z Lipid
GranzymeB
25.0m/z Lipid
57.0m/z Lipid
85.0m/z Lipid
81.0m/z Lipid
80.0m/z Lipid
82.0m/z Lipid
56.0m/z Lipid
26.0m/z Lipid
74.0m/z Glycine
122.0m/z
98.0m/z Lipid
64.0m/z Lipid
89.0m/z Lactic acid
88.0m/z Alanine
65.0m/z Lipid
146.0m/z Glutamic acid
87.0m/z Pyruvic acid
PanKeratin
26.0m/z Lipid
74.0m/z Glycine
98.0m/z Lipid
122.0m/z
64.0m/z Lipid
58.0m/z Lipid
88.0m/z Alanine
89.0m/z Lactic acid
146.0m/z Glutamic acid
65.0m/z Lipid
25.0m/z Lipid
45.0m/z Lipid
119.0m/z
181.0m/z Lipid
SMA
25.0m/z Lipid
109.0m/z Cholesterol fragment
85.0m/z Lipid
26.0m/z Lipid
74.0m/z Glycine
98.0m/z Lipid
122.0m/z
119.0m/z
58.0m/z Lipid
Vimentin
25.0m/z Lipid
80.0m/z Lipid
41.0m/z Lipid
70.0m/z Lipid
67.0m/z Lipid
131.0m/z Asparagine
26.0m/z Lipid
74.0m/z Glycine
98.0m/z Lipid
122.0m/z
58.0m/z Lipid
146.0m/z Glutamic acid
88.0m/z Alanine
96.0m/z Lipid
Protein intensity
low to high
0.4
0.2
0.0
−0.2
−0.4
Marker expression

Supplementary Fig. 13. Single-cell metabolite channels associated with glycose, lipid, amino acids, and fatty acids correlated with protein profile in lung cancer tissues. Each heatmap shows the correlation of metabolite channels with corresponding protein markers (n=19507 cells). Left to right shows ascending single-cell protein intensity expression.

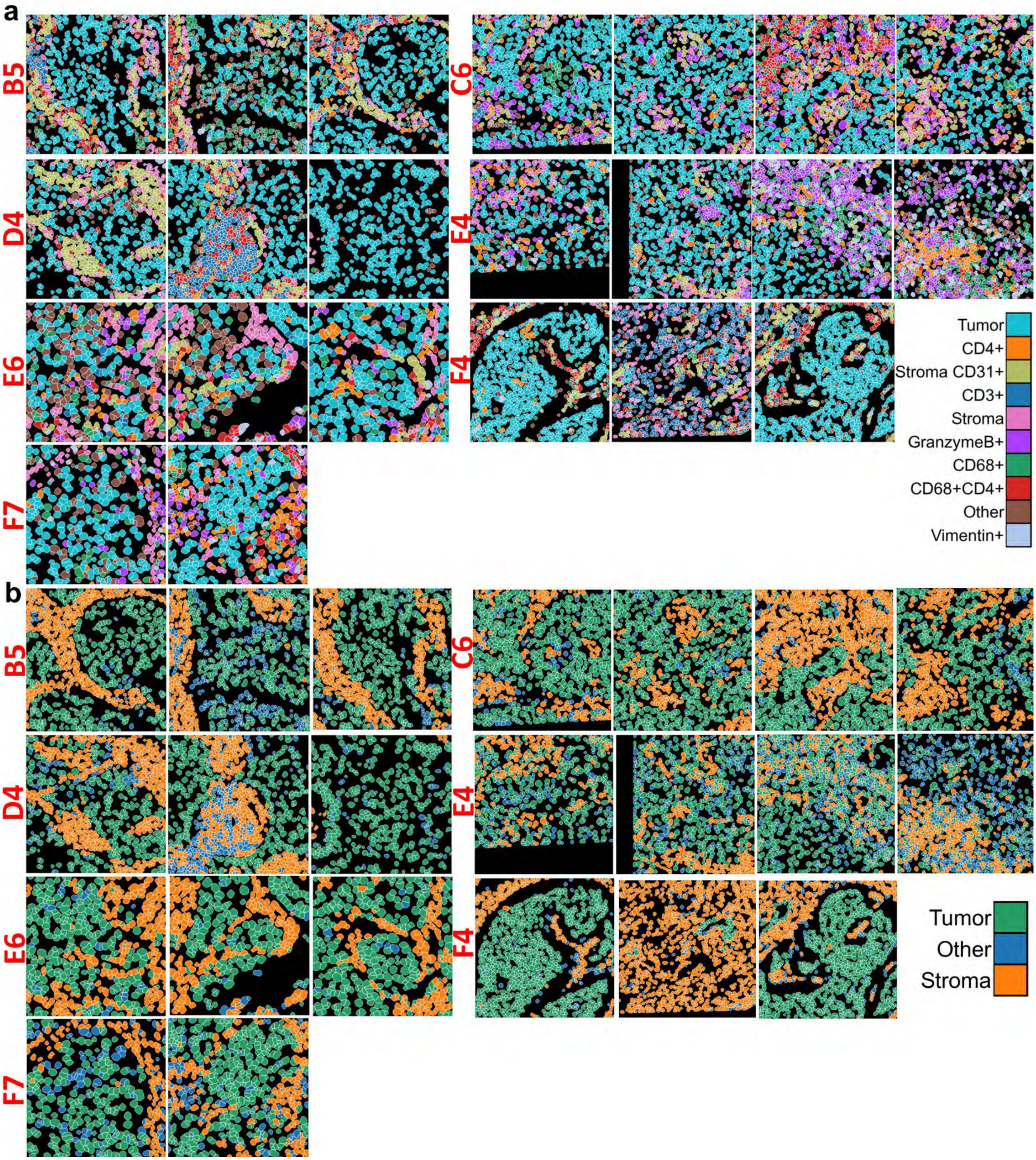

a
B5
C6
D4
E4
E6
F4
F7
Tumor
CD4+
Stroma CD31+
CD3+
Stroma
GranzymeB+
CD68+
CD68+CD4+
Other
Vimentin+
b
B5
C6
D4
E4
E6
F4
F7
Tumor
Other
Stroma

Supplementary Fig. 14. Single-cell protein phenotyping in cancer lung cancer

a Spatial projection of unsupervised single cell clusters from protein profiles in human lung cancer tissues for all imaged regions of interest.

b Classification of cells into the tumor, stroma, and other regions from cell clusters obtained from **a**.

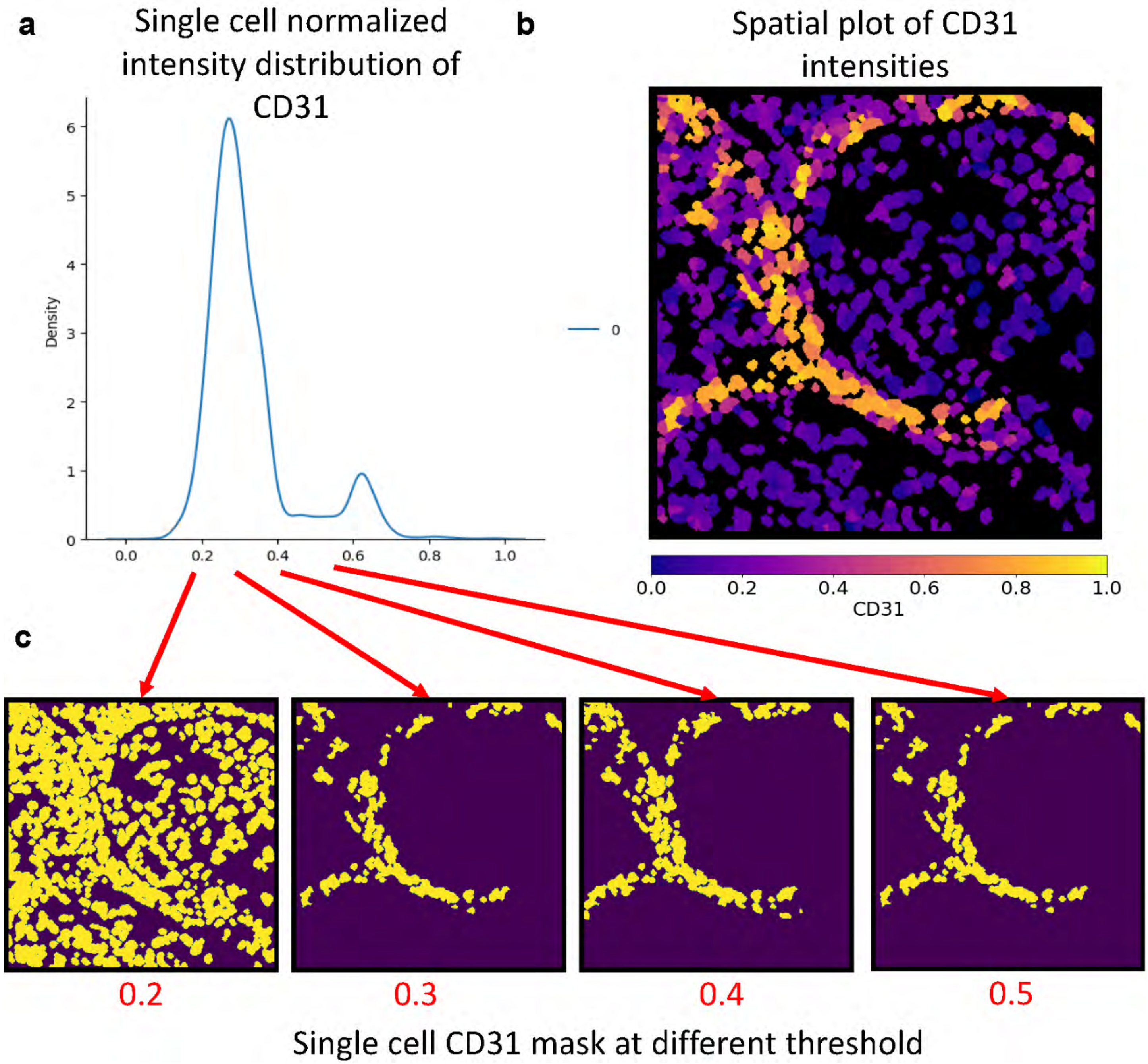
a
Single cell normalized intensity distribution of CD31
Density
0
1
2
3
4
5
6
0.0
0.2
0.4
0.6
0.8
1.0
0
b
Spatial plot of CD31 intensities
0.0
0.2
0.4
0.6
0.8
1.0
CD31
c
0.2
0.3
0.4
0.5
Single cell CD31 mask at different threshold

119

Supplementary Fig. 15. CD31 marker threshold at the single cell level

a Bi-modal distribution of single-cell mean intensity of CD31 markers.

b Corresponding single-cell mask with mean intensity plotted corresponding to the distribution in **a**.

c Example of CD31 positive single-cell masks at different thresholds: 0.2, 0.3, 0.4 and 0.5.

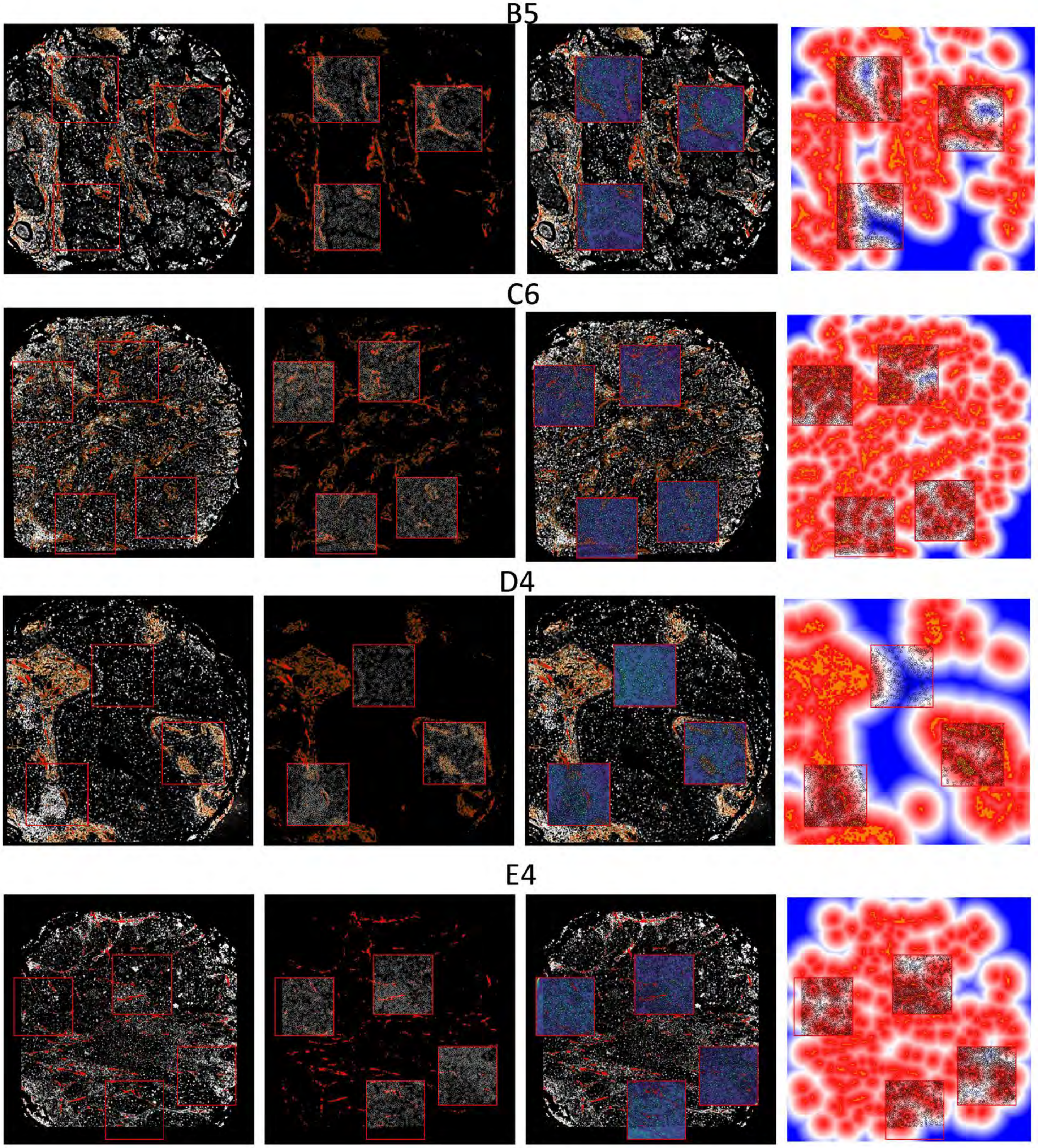
B5
C6
D4
E4

Supplementary Fig. 16. Single-cell to. CD31+ cell distance matching. Left to right show respectively: IMC multiplex HistoneH3 marker (white) superposed with CD31 marker (red) and corresponding matched 3D-SMF imaged regions showed inside the red bounding box (far left), IMC CD31 marker (red) and corresponding matched 3D-SMF imaged regions in the red bounding box (left center), IMC CD31 marker (red) and corresponding matched PO3- channel from 3D-SMF regions (right center), distance to CD31 positive cell in the spatial domain and corresponding single cell mask in imaged 3D-SMF regions (far right).

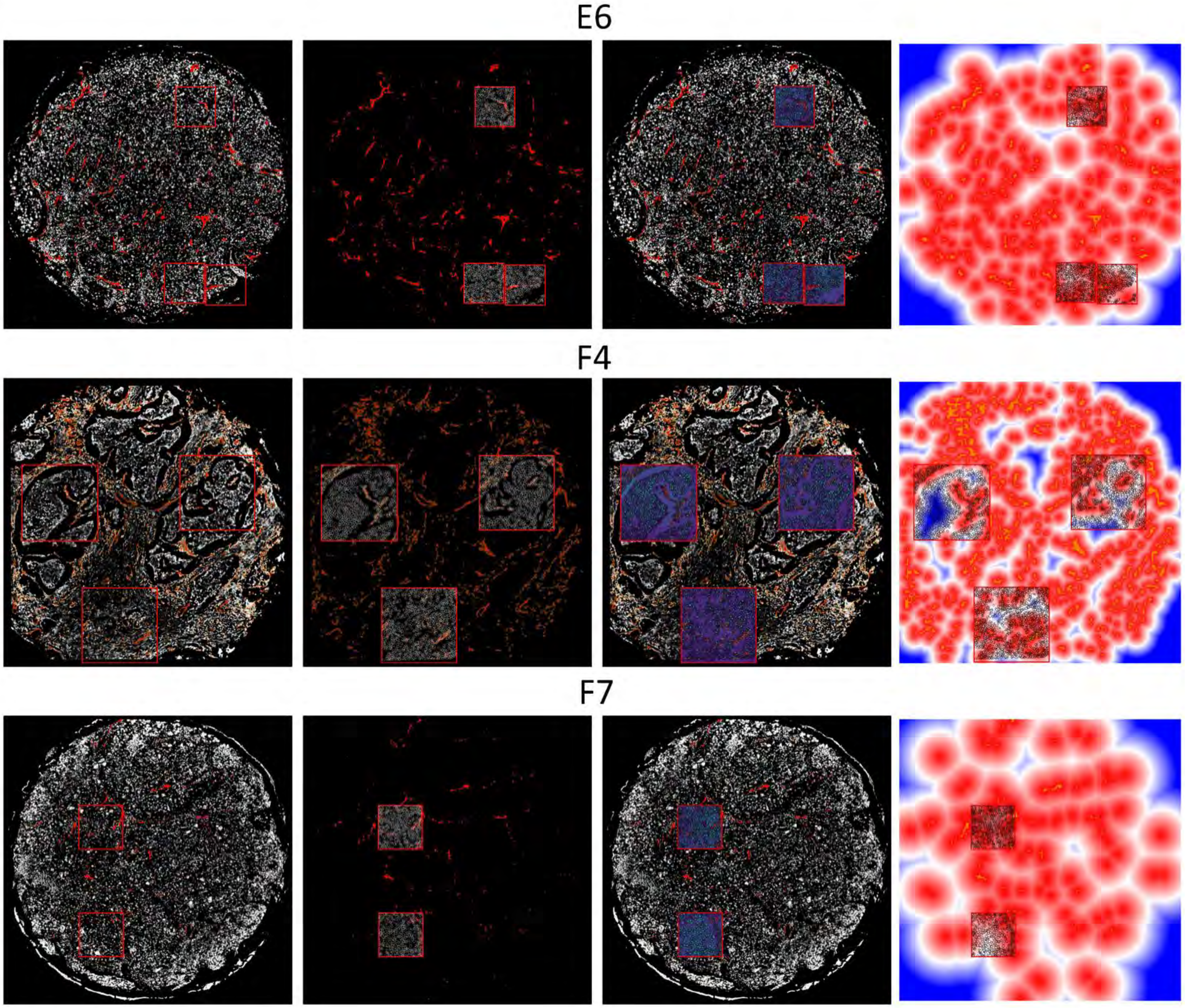
E6
F4
F7

Supplementary Fig. 17. Single-cell to. CD31+ cell distance matching. Left to right show respectively: IMC multiplex Histone H3 marker (white) superposed with CD31 marker (red) and corresponding matched 3D-SMF imaged regions showed inside the red bounding box (far left), IMC CD31 marker (red) and corresponding matched 3D-SMF imaged regions in the red bounding box (left center), IMC CD31 marker (red) and corresponding matched PO3- channel from 3D-SMF regions (right center), distance to CD31 positive cell in the spatial domain and corresponding single cell mask in imaged 3D-SMF regions (far right).

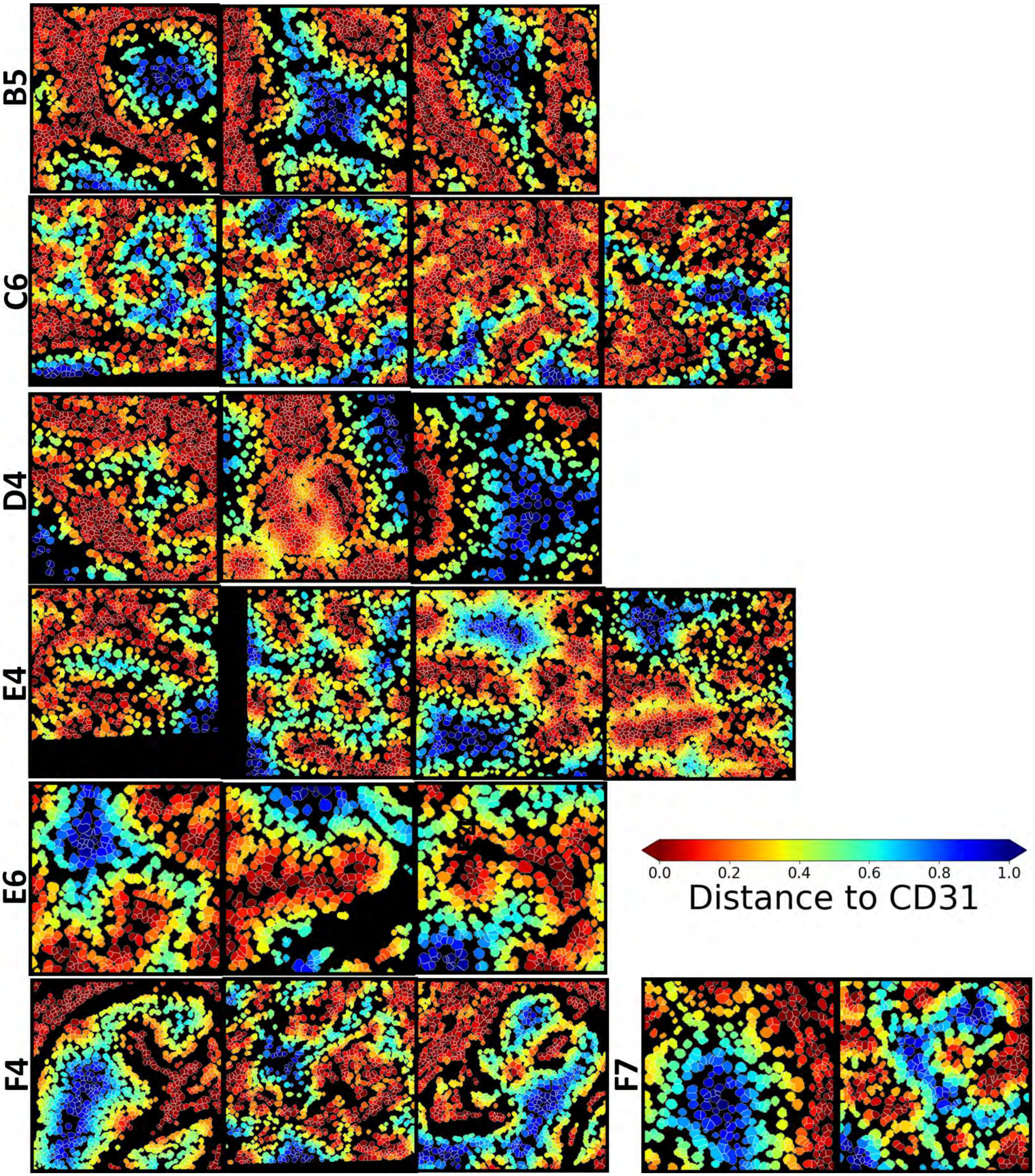
B5
C6
D4
E4
E6
F4
F7
0.0
0.2
0.4
0.6
0.8
1.0
Distance to CD31

Supplementary Fig. 18. Single-cell CD31 cells distance map in lung cancer tissues. Single-cell masks in lung cancer tissues with corresponding distance to CD31+ cells overlaid.

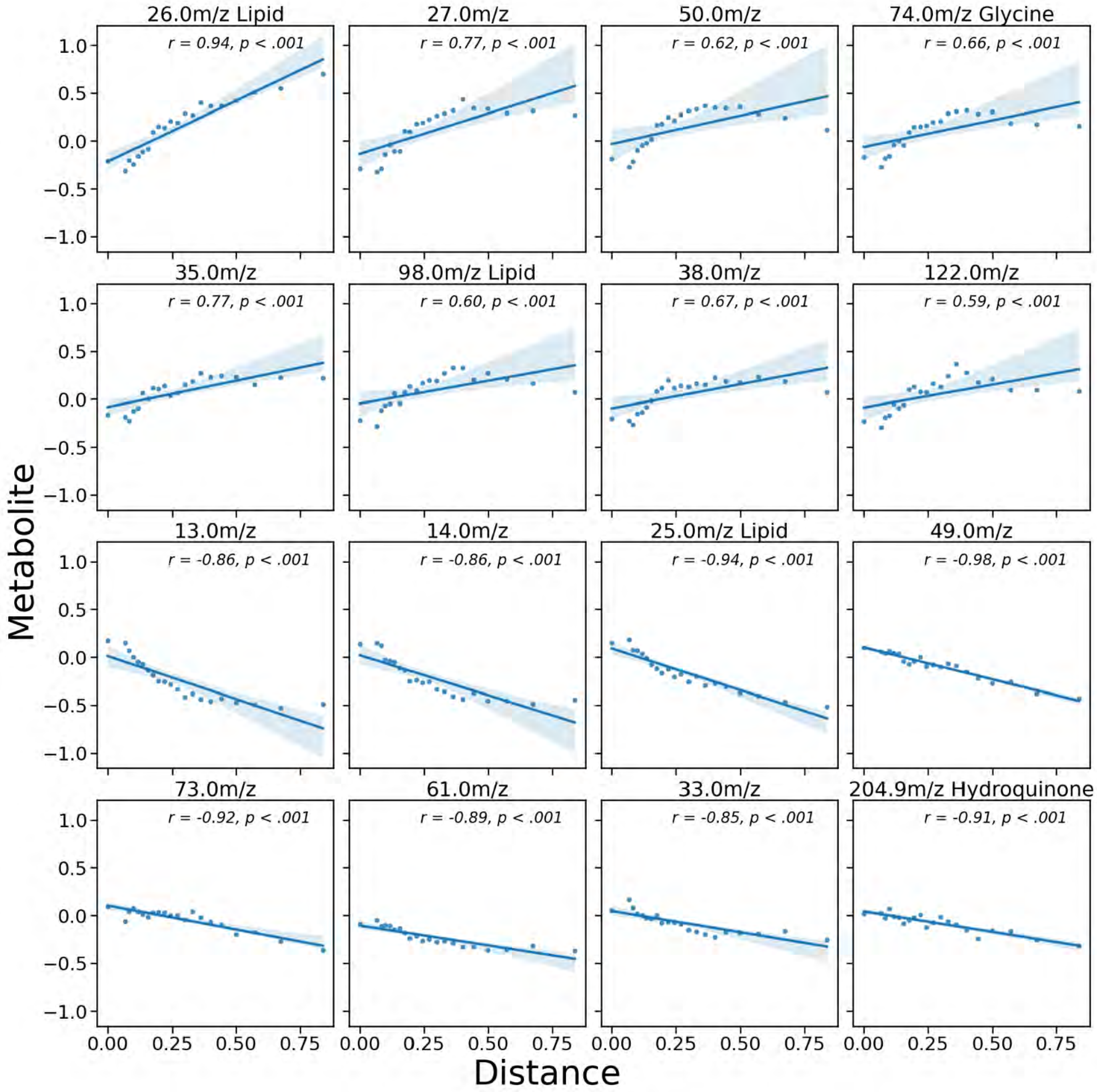

26.0m/z Lipid
r = 0.94, p < .001
27.0m/z
r = 0.77, p < .001
50.0m/z
r = 0.62, p < .001
74.0m/z Glycine
r = 0.66, p < .001
35.0m/z
r = 0.77, p < .001
98.0m/z Lipid
r = 0.60, p < .001
38.0m/z
r = 0.67, p < .001
122.0m/z
r = 0.59, p < .001
13.0m/z
r = -0.86, p < .001
14.0m/z
r = -0.86, p < .001
25.0m/z Lipid
r = -0.94, p < .001
49.0m/z
r = -0.98, p < .001
73.0m/z
r = -0.92, p < .001
61.0m/z
r = -0.89, p < .001
33.0m/z
r = -0.85, p < .001
204.9m/z Hydroquinone
r = -0.91, p < .001
Metabolite
Distance

Supplementary Fig. 19. Correlation plot of most and least correlated metabolite channels with distance to CD31 cells. Pearson correlation and p-value calculated from 25 bins distance defined with n=19507 cells.

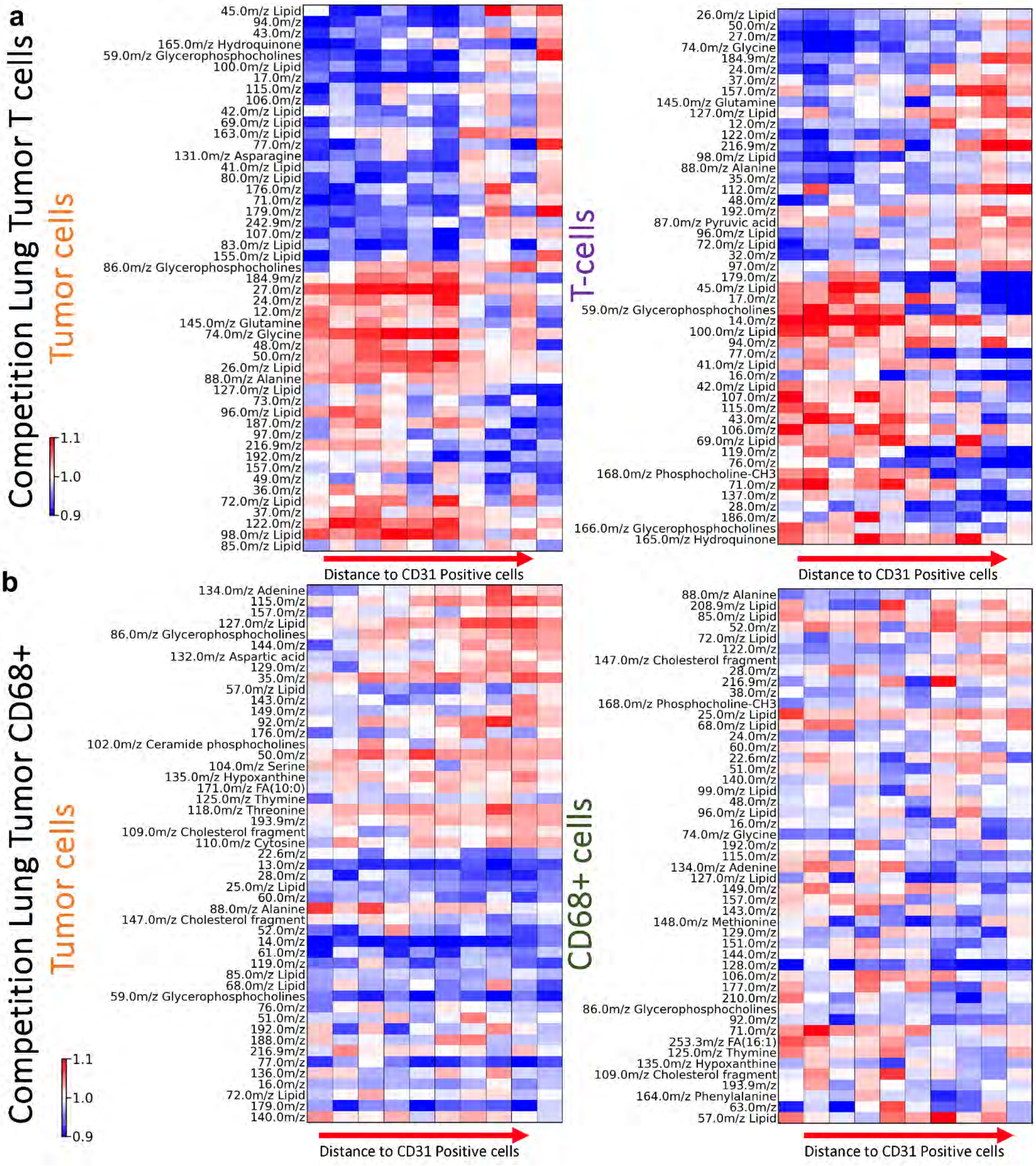
a
Competition Lung Tumor T cells
Tumor cells
45.0m/z Lipid
94.0m/z
43.0m/z
165.0m/z Hydroquinone
59.0m/z Glycerophosphocholines
100.0m/z Lipid
17.0m/z
115.0m/z
106.0m/z
42.0m/z Lipid
69.0m/z Lipid
163.0m/z Lipid
77.0m/z
131.0m/z Asparagine
41.0m/z Lipid
80.0m/z Lipid
176.0m/z
71.0m/z
179.0m/z
242.9m/z
107.0m/z
83.0m/z Lipid
155.0m/z Lipid
86.0m/z Glycerophosphocholines
184.9m/z
27.0m/z
24.0m/z
12.0m/z
145.0m/z Glutamine
74.0m/z Glycine
48.0m/z
50.0m/z
26.0m/z Lipid
88.0m/z Alanine
127.0m/z Lipid
73.0m/z
96.0m/z Lipid
187.0m/z
97.0m/z
216.9m/z
192.0m/z
157.0m/z
49.0m/z
36.0m/z
72.0m/z Lipid
37.0m/z
122.0m/z
98.0m/z Lipid
85.0m/z Lipid
1.1
1.0
0.9
Distance to CD31 Positive cells
T-cells
26.0m/z Lipid
50.0m/z
27.0m/z
74.0m/z Glycine
184.9m/z
24.0m/z
37.0m/z
157.0m/z
145.0m/z Glutamine
127.0m/z Lipid
12.0m/z
122.0m/z
216.9m/z
98.0m/z Lipid
88.0m/z Alanine
35.0m/z
112.0m/z
48.0m/z
192.0m/z
87.0m/z Pyruvic acid
96.0m/z Lipid
72.0m/z Lipid
32.0m/z
97.0m/z
179.0m/z
45.0m/z Lipid
17.0m/z
59.0m/z Glycerophosphocholines
14.0m/z
100.0m/z Lipid
94.0m/z
77.0m/z
41.0m/z Lipid
16.0m/z
42.0m/z Lipid
107.0m/z
115.0m/z
43.0m/z
106.0m/z
69.0m/z Lipid
119.0m/z
76.0m/z
168.0m/z Phosphocholine-CH3
71.0m/z
137.0m/z
28.0m/z
186.0m/z
166.0m/z Glycerophosphocholines
165.0m/z Hydroquinone
Distance to CD31 Positive cells
b
Competition Lung Tumor CD68+
Tumor cells
134.0m/z Adenine
115.0m/z
157.0m/z
127.0m/z Lipid
86.0m/z Glycerophosphocholines
144.0m/z
132.0m/z Aspartic acid
129.0m/z
35.0m/z
57.0m/z Lipid
143.0m/z
149.0m/z
92.0m/z
176.0m/z
102.0m/z Ceramide phosphocholines
50.0m/z
104.0m/z Serine
135.0m/z Hypoxanthine
171.0m/z FA(10:0)
125.0m/z Thymine
118.0m/z Threonine
193.9m/z
109.0m/z Cholesterol fragment
110.0m/z Cytosine
22.6m/z
13.0m/z
28.0m/z
25.0m/z Lipid
60.0m/z
88.0m/z Alanine
147.0m/z Cholesterol fragment
52.0m/z
14.0m/z
61.0m/z
119.0m/z
85.0m/z Lipid
68.0m/z Lipid
59.0m/z Glycerophosphocholines
76.0m/z
51.0m/z
192.0m/z
188.0m/z
216.9m/z
77.0m/z
136.0m/z
16.0m/z
72.0m/z Lipid
179.0m/z
140.0m/z
1.1
1.0
0.9
Distance to CD31 Positive cells
CD68+ cells
88.0m/z Alanine
208.9m/z Lipid
85.0m/z Lipid
52.0m/z
72.0m/z Lipid
122.0m/z
147.0m/z Cholesterol fragment
28.0m/z
216.9m/z
38.0m/z
168.0m/z Phosphocholine-CH3
25.0m/z Lipid
68.0m/z Lipid
24.0m/z
60.0m/z
22.6m/z
51.0m/z
140.0m/z
99.0m/z Lipid
48.0m/z
96.0m/z Lipid
16.0m/z
74.0m/z Glycine
192.0m/z
115.0m/z
134.0m/z Adenine
127.0m/z Lipid
149.0m/z
157.0m/z
143.0m/z
148.0m/z Methionine
129.0m/z
151.0m/z
144.0m/z
128.0m/z
106.0m/z
177.0m/z
210.0m/z
86.0m/z Glycerophosphocholines
92.0m/z
71.0m/z
253.3m/z FA(16:1)
125.0m/z Thymine
135.0m/z Hypoxanthine
109.0m/z Cholesterol fragment
193.9m/z
164.0m/z Phenylalanine
63.0m/z
57.0m/z Lipid
Distance to CD31 Positive cells

Supplementary Fig. 20. Single-cell metabolites competition as distance to CD31 positive cells in lung cancer tissues.

- **a** Local metabolites competition as a distance of CD31+ cells between T-cells and tumor cells (n = 6536 cells). Selected metabolite channels showing positive and negative correlation of tumor cells (left) and T-cells (right). Colorbar shows the metabolites competition ratio.
- **b** Local metabolites competition as a distance of CD31+ cells between CD68+ cells and tumor cells (n = 8130 cells). Selected metabolite channels showing positive and negative correlation of tumor cells (left) and CD68+ cells (right). Colorbar shows the metabolites competition ratio.

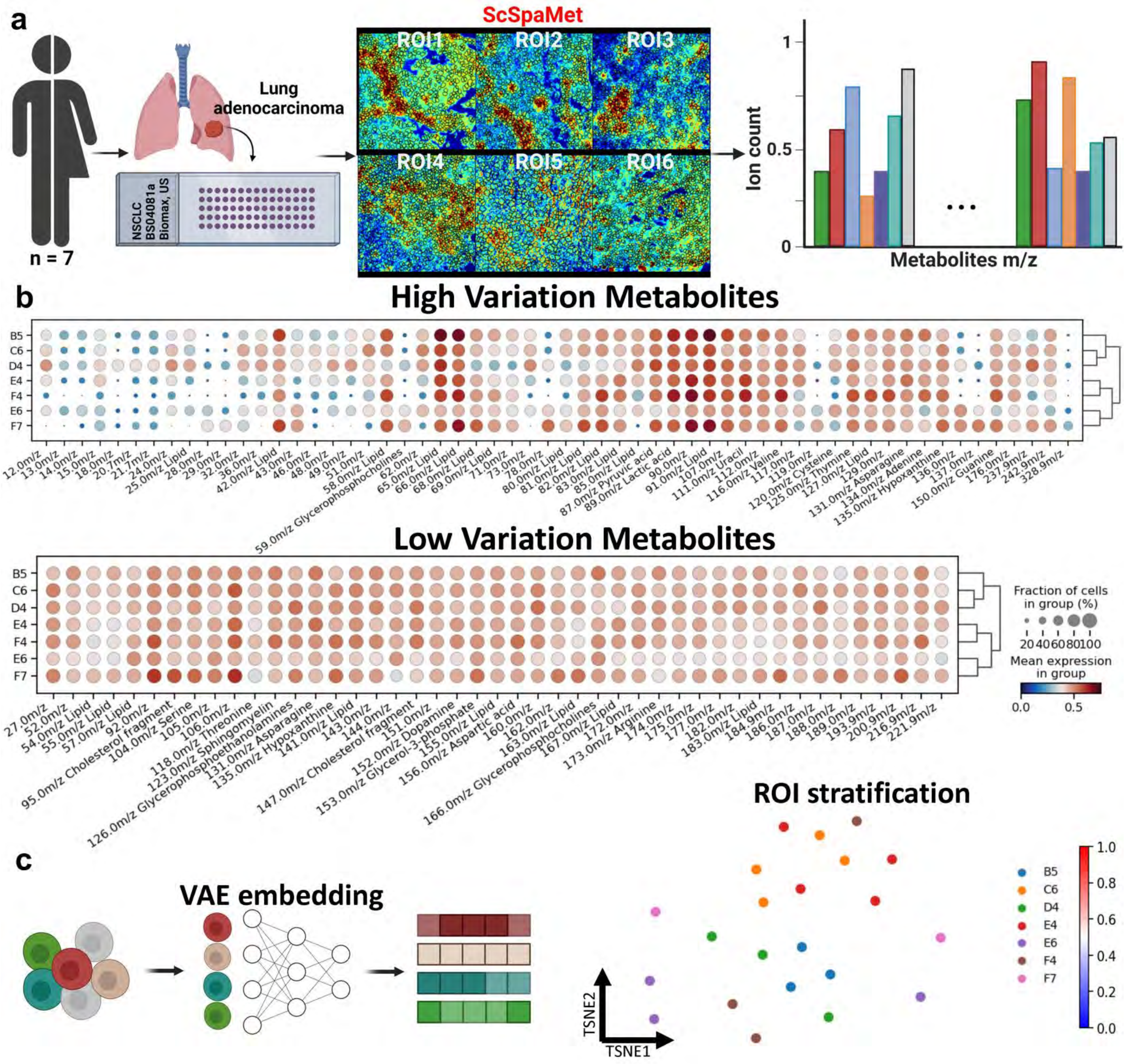
a
n = 7
Lung adenocarcinoma
NSCLC BS04081a Biomax, US
ScSpaMet
ROI1
ROI2
ROI3
ROI4
ROI5
ROI6
Ion count
Metabolites m/z
b
High Variation Metabolites
B5
C6
D4
E4
F4
E6
F7
12.0m/z 13.0m/z 14.0m/z 15.0m/z 18.0m/z 20.7m/z 21.7m/z 24.0m/z 25.0m/z Lipid 28.0m/z 29.0m/z 32.0m/z 36.0m/z 42.0m/z Lipid 43.0m/z 46.0m/z 48.0m/z 49.0m/z 51.0m/z 58.0m/z Lipid 59.0m/z Glycerophosphocholines 62.0m/z 65.0m/z Lipid 66.0m/z Lipid 68.0m/z Lipid 69.0m/z Lipid 71.0m/z 73.0m/z 78.0m/z 80.0m/z Lipid 81.0m/z Lipid 82.0m/z Lipid 83.0m/z Lipid 85.0m/z Lipid 87.0m/z Pyruvic acid 89.0m/z Lactic acid 90.0m/z 91.0m/z Lipid 107.0m/z 111.0m/z Uracil 112.0m/z 116.0m/z Valine 117.0m/z 119.0m/z 120.0m/z Cysteine 125.0m/z Thymine 127.0m/z Lipid 129.0m/z 131.0m/z Asparagine 134.0m/z Adenine 135.0m/z Hypoxanthine 136.0m/z 137.0m/z 150.0m/z Guanine 176.0m/z 237.9m/z 242.9m/z 328.9m/z
Low Variation Metabolites
27.0m/z 52.0m/z 54.0m/z Lipid 55.0m/z Lipid 57.0m/z Lipid 92.0m/z 95.0m/z Cholesterol fragment 104.0m/z Serine 105.0m/z 106.0m/z 118.0m/z Threonine 123.0m/z Sphingomyelin 126.0m/z Glycerophosphoethanolamines 131.0m/z Asparagine 135.0m/z Hypoxanthine 141.0m/z Lipid 143.0m/z 144.0m/z 147.0m/z Cholesterol fragment 151.0m/z 152.0m/z Dopamine 153.0m/z Glycerol-3-phosphate 155.0m/z Lipid 156.0m/z Aspartic acid 160.0m/z 162.0m/z 163.0m/z Lipid 166.0m/z Glycerophosphocholines 167.0m/z Lipid 172.0m/z 173.0m/z Arginine 174.0m/z 175.0m/z 177.0m/z 182.0m/z 183.0m/z Lipid 184.9m/z 186.0m/z 187.0m/z 188.0m/z 189.0m/z 193.9m/z 200.9m/z 216.9m/z 221.9m/z
Fraction of cells in group (%)
20 40 60 80 100
Mean expression in group
0.0 0.5
c
VAE embedding
ROI stratification
TSNE2
TSNE1
B5
C6
D4
E4
E6
F4
F7
1.0 0.8 0.6 0.4 0.2 0.0

Supplementary Fig. 21. ScSpaMet stratifies lung cancer patients and shows cell-specific metabolite states.

- **a** Representative schematic showing the single cell metabolites comparison across patients in lung tumor tissues. Created with Biorender.com
- **b** Dot plot showing high variation (top) and low variation (bottom) metabolite channels across patients in lung tumor tissues.
- **c** VAE single cell protein-metabolite joint embedding showing stratification of patients and joint protein-metabolites single cell phenotype. Created with Biorender.com

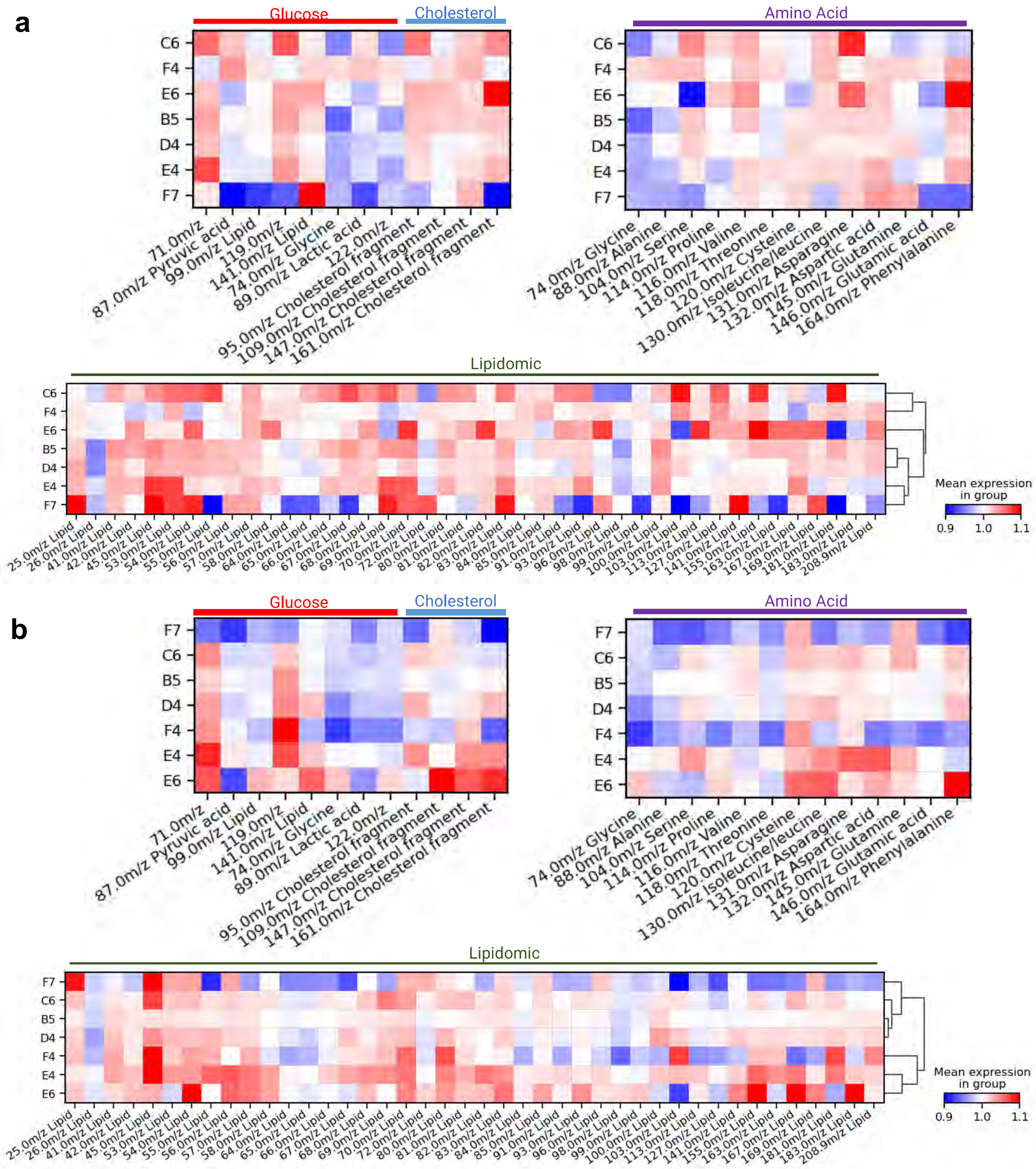
a
Glucose
Cholesterol
Amino Acid
C6
F4
E6
B5
D4
E4
F7
71.0m/z
87.0m/z Pyruvic acid
99.0m/z Lipid
119.0m/z
141.0m/z Lipid
74.0m/z Glycine
89.0m/z Lactic acid
122.0m/z
95.0m/z Cholesterol fragment
109.0m/z Cholesterol fragment
147.0m/z Cholesterol fragment
161.0m/z Cholesterol fragment
74.0m/z Glycine
88.0m/z Alanine
104.0m/z Serine
114.0m/z Proline
116.0m/z Valine
118.0m/z Threonine
120.0m/z Cysteine
130.0m/z Isoleucine/leucine
131.0m/z Asparagine
132.0m/z Aspartic acid
145.0m/z Glutamine
146.0m/z Glutamic acid
164.0m/z Phenylalanine
Lipidomic
25.0m/z Lipid
26.0m/z Lipid
41.0m/z Lipid
42.0m/z Lipid
45.0m/z Lipid
53.0m/z Lipid
54.0m/z Lipid
55.0m/z Lipid
56.0m/z Lipid
57.0m/z Lipid
58.0m/z Lipid
64.0m/z Lipid
65.0m/z Lipid
66.0m/z Lipid
67.0m/z Lipid
68.0m/z Lipid
69.0m/z Lipid
70.0m/z Lipid
72.0m/z Lipid
80.0m/z Lipid
81.0m/z Lipid
82.0m/z Lipid
83.0m/z Lipid
84.0m/z Lipid
85.0m/z Lipid
91.0m/z Lipid
93.0m/z Lipid
96.0m/z Lipid
98.0m/z Lipid
99.0m/z Lipid
100.0m/z Lipid
103.0m/z Lipid
113.0m/z Lipid
127.0m/z Lipid
141.0m/z Lipid
155.0m/z Lipid
163.0m/z Lipid
167.0m/z Lipid
169.0m/z Lipid
181.0m/z Lipid
183.0m/z Lipid
208.9m/z Lipid
Mean expression in group
0.9
1.0
1.1
b
Glucose
Cholesterol
Amino Acid
F7
C6
B5
D4
F4
E4
E6
Lipidomic
Mean expression in group
0.9
1.0
1.1

Supplementary Fig. 22 Single-cell joint metabolite competition for each TMA core for **a** Tumor and T-cells (n = 6536 cells) and **b** Tumor and CD68+ cells (n = 8130 cells).

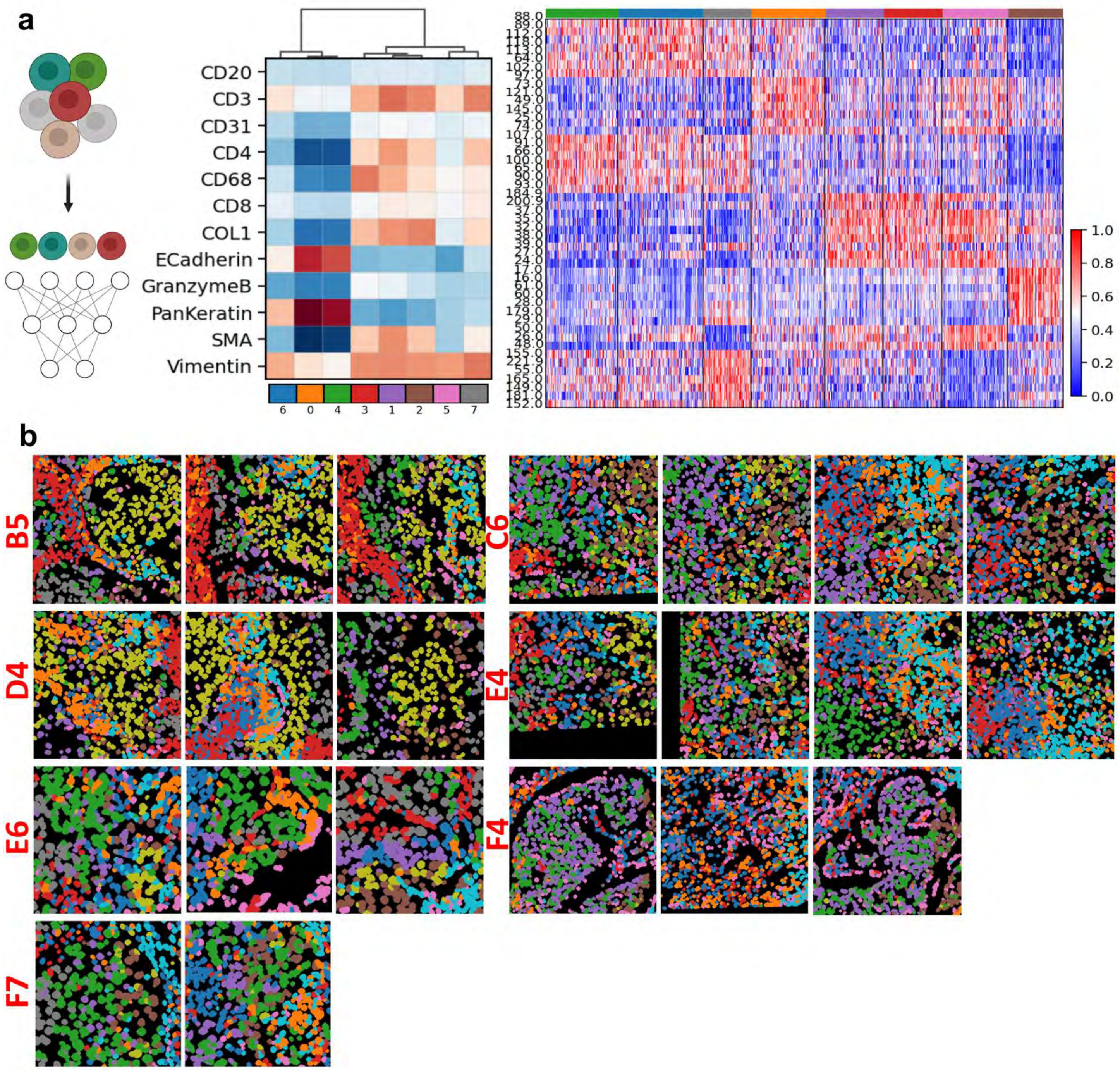

a
CD20
CD3
CD31
CD4
CD68
CD8
COL1
ECadherin
GranzymeB
PanKeratin
SMA
Vimentin
6
0
4
3
1
2
5
7
1.0
0.8
0.6
0.4
0.2
0.0
b
B5
C6
D4
E4
E6
F4
F7

Supplementary Fig. 23. Single-cell joint protein-metabolite phenotyping in lung cancer.

- **a** VAE single cell protein-metabolite joint embedding. Left: architecture for single-cell protein-metabolite joint embedding (n=19507 cells). Right: joint protein-metabolites single-cell phenotype. The colorbar shows the single cell mean expression value. Created with Biorender.com
- **b** Spatial projection of clusters obtained from a) in human lung cancer tissues for all imaged regions of interest.

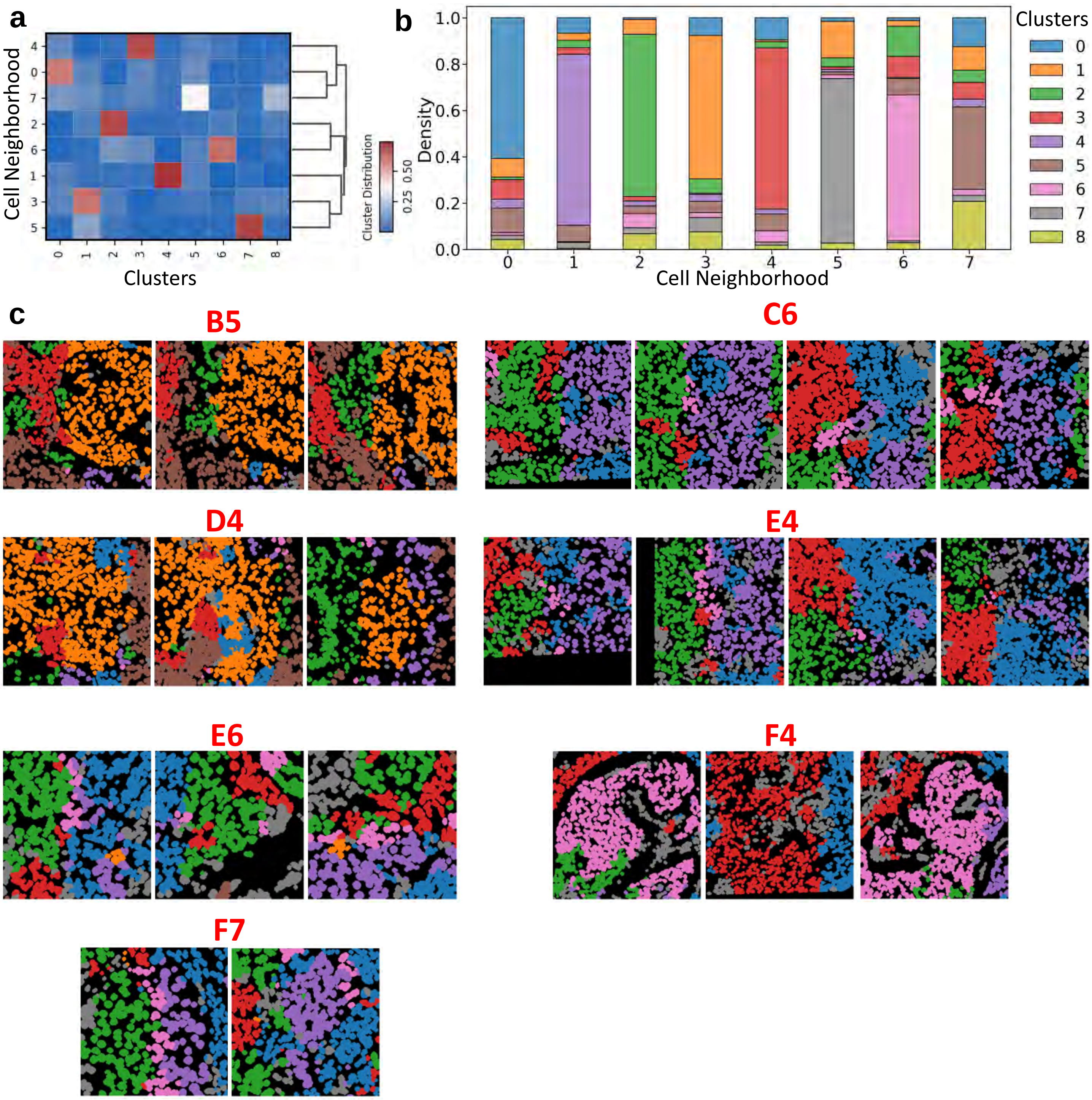

a
Cell Neighborhood
Clusters
Cluster Distribution
0.25 0.50
b
Density
Cell Neighborhood
Clusters
c
B5
C6
D4
E4
E6
F4
F7

Supplementary Fig. 24. Single-cell joint protein-metabolite signatures in lung cancer (n=19507 cells).

a Count of each unsupervised cluster in all the defined cell neighborhoods.

b The density of each unsupervised cluster in all defined cell neighborhoods.

c Spatial projection of cell neighborhood obtained from **a** and **b** in human lung cancer tissues for all imaged regions of interest.

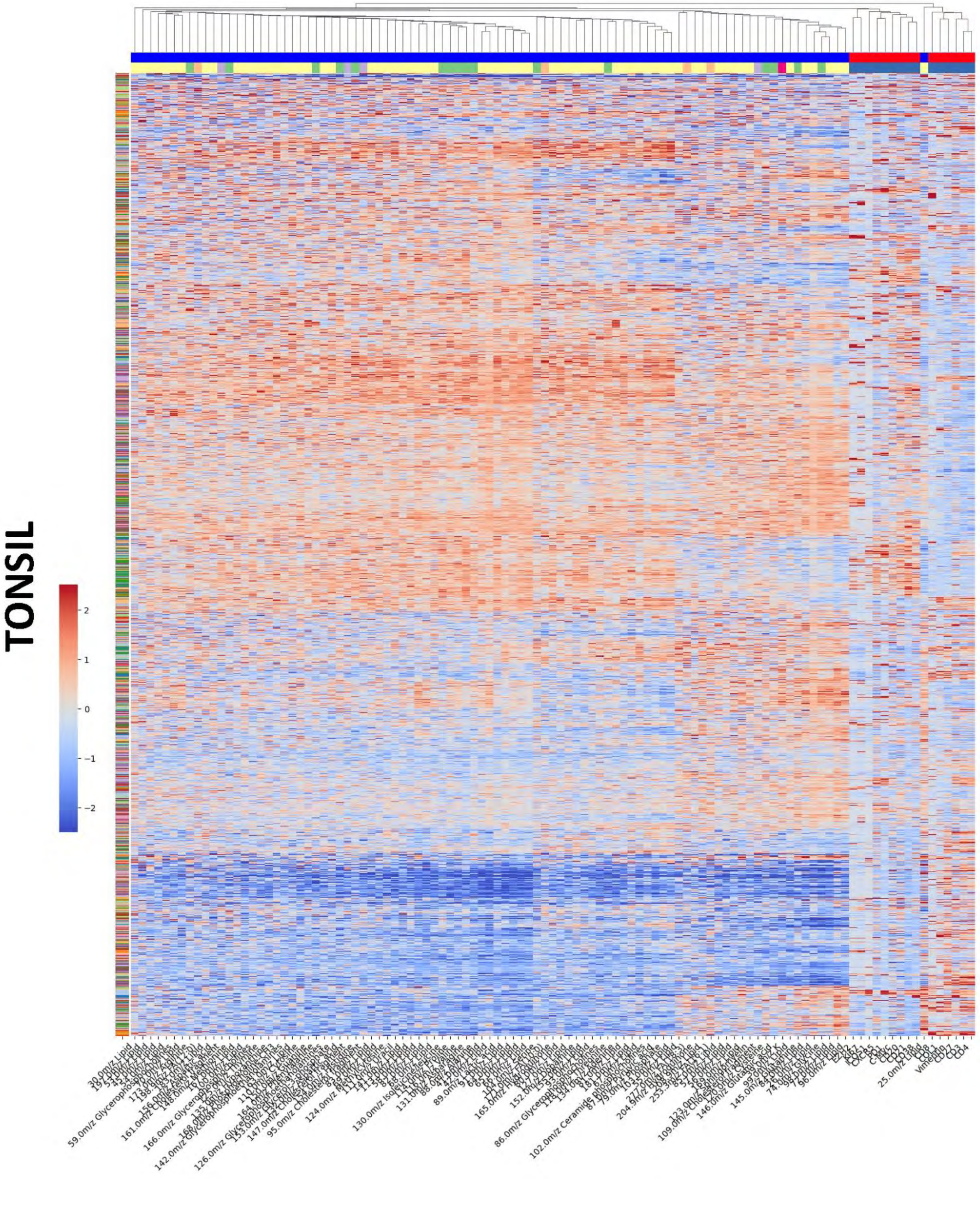
TONSIL
2
1
0
−1
−2

Supplementary Fig. 25. Single-cell metabolite and protein profile in tonsil tissues (n=31156 cells). Cluster map showing the cell-level metabolite and protein profile in all tonsil tissues. Color corresponds to the mean intensity value at the single cell level.

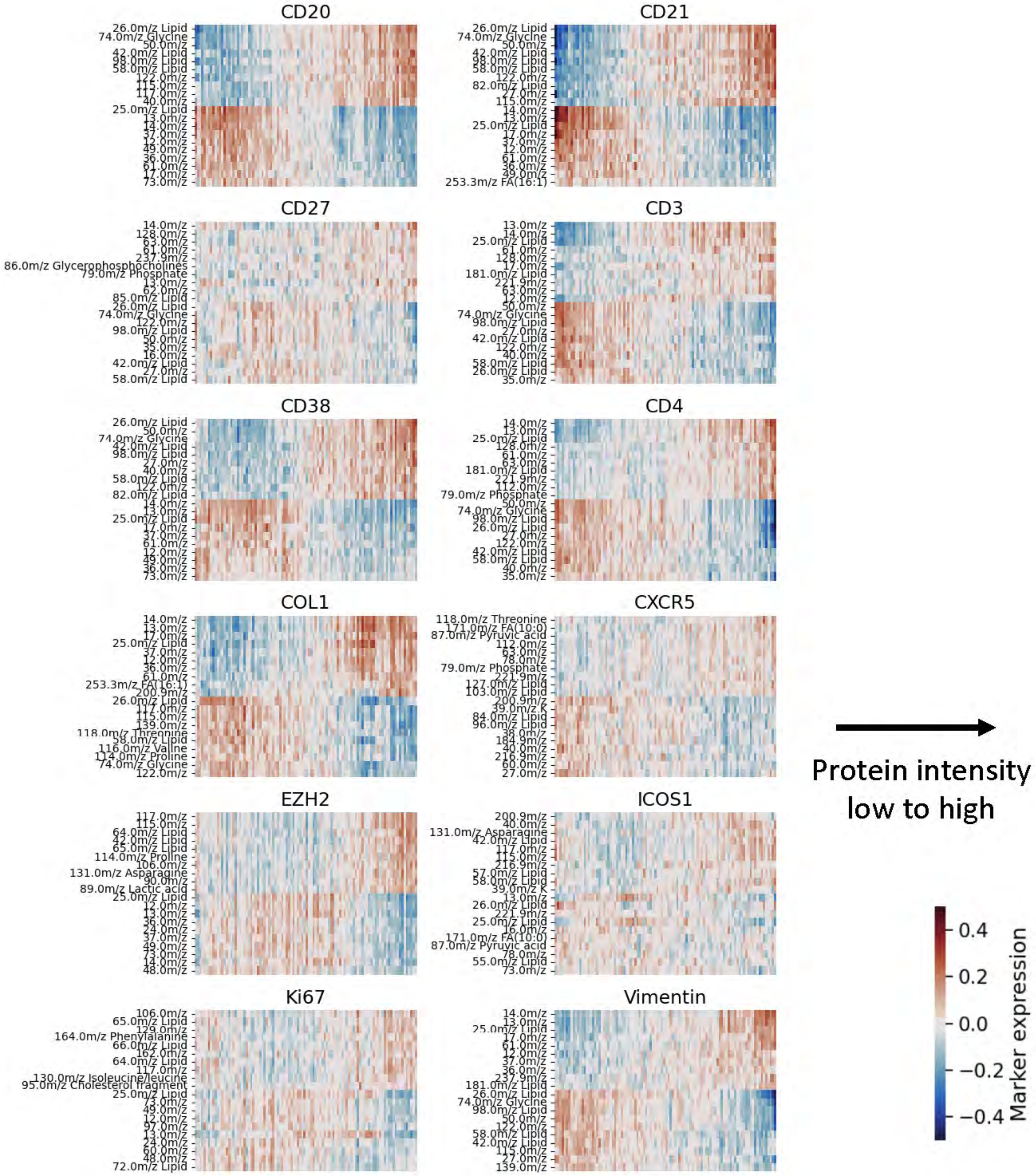
CD20
CD21
CD27
CD3
CD38
CD4
COL1
CXCR5
EZH2
ICOS1
Ki67
Vimentin
Protein intensity
low to high
Marker expression
0.4
0.2
0.0
−0.2
−0.4

Supplementary Fig. 26. Single-cell metabolite and protein profile correlation in tonsil tissues. Each heatmap shows the correlation of metabolite channels with corresponding protein markers (n=31156 cells). Left to right shows ascending single-cell protein intensity expression.

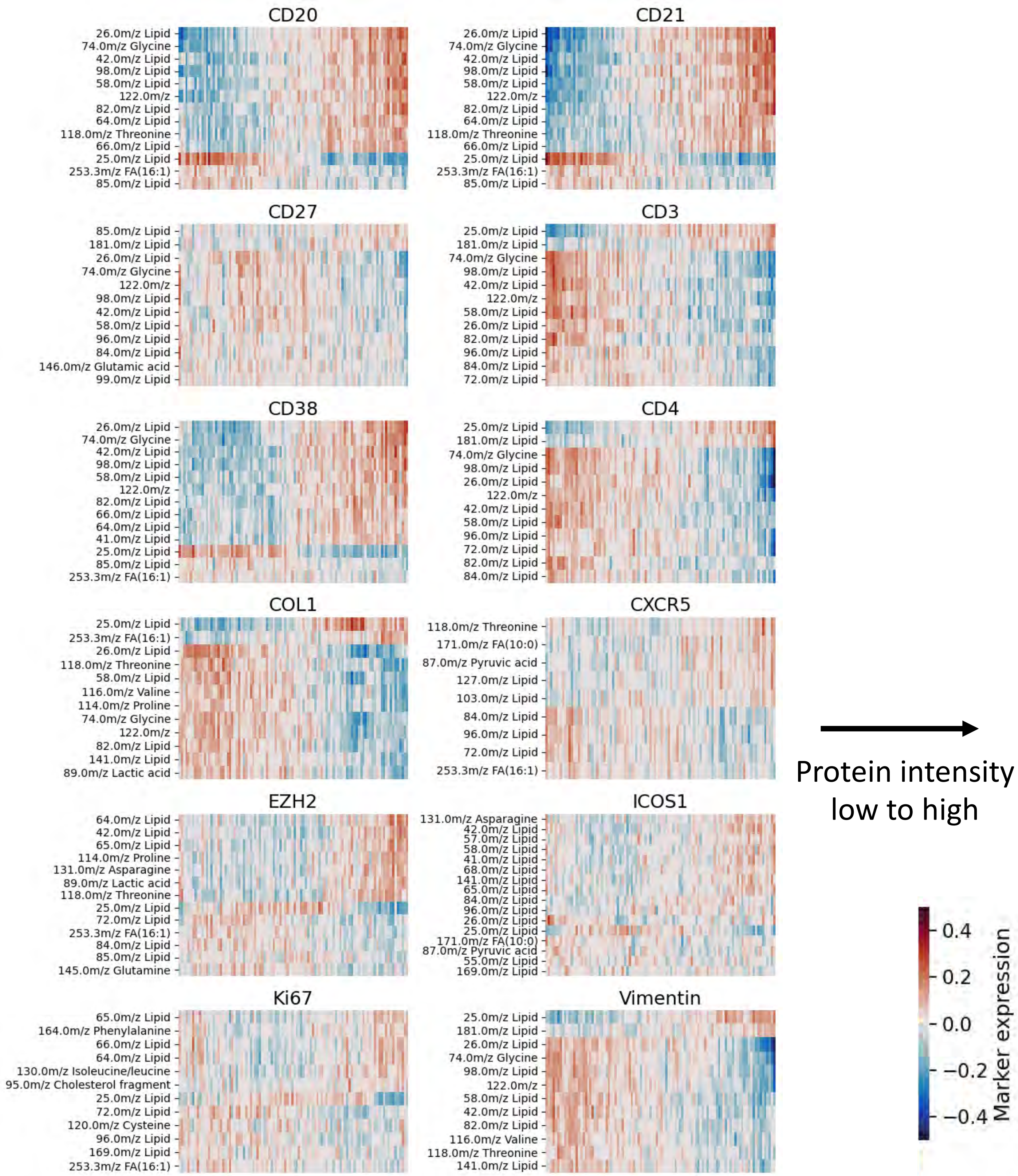
CD20
CD21
CD27
CD3
CD38
CD4
COL1
CXCR5
EZH2
ICOS1
Ki67
Vimentin
Protein intensity low to high
Marker expression
0.4
0.2
0.0
−0.2
−0.4

Supplementary Fig. 27. Single-cell metabolite channels associated with glycose, lipid, amino acids, and fatty acids correlated with protein profile in tonsil tissues (n=31156 cells). Each heatmap shows the correlation of metabolite channels with corresponding protein markers. Left to right shows ascending single-cell protein intensity expression.

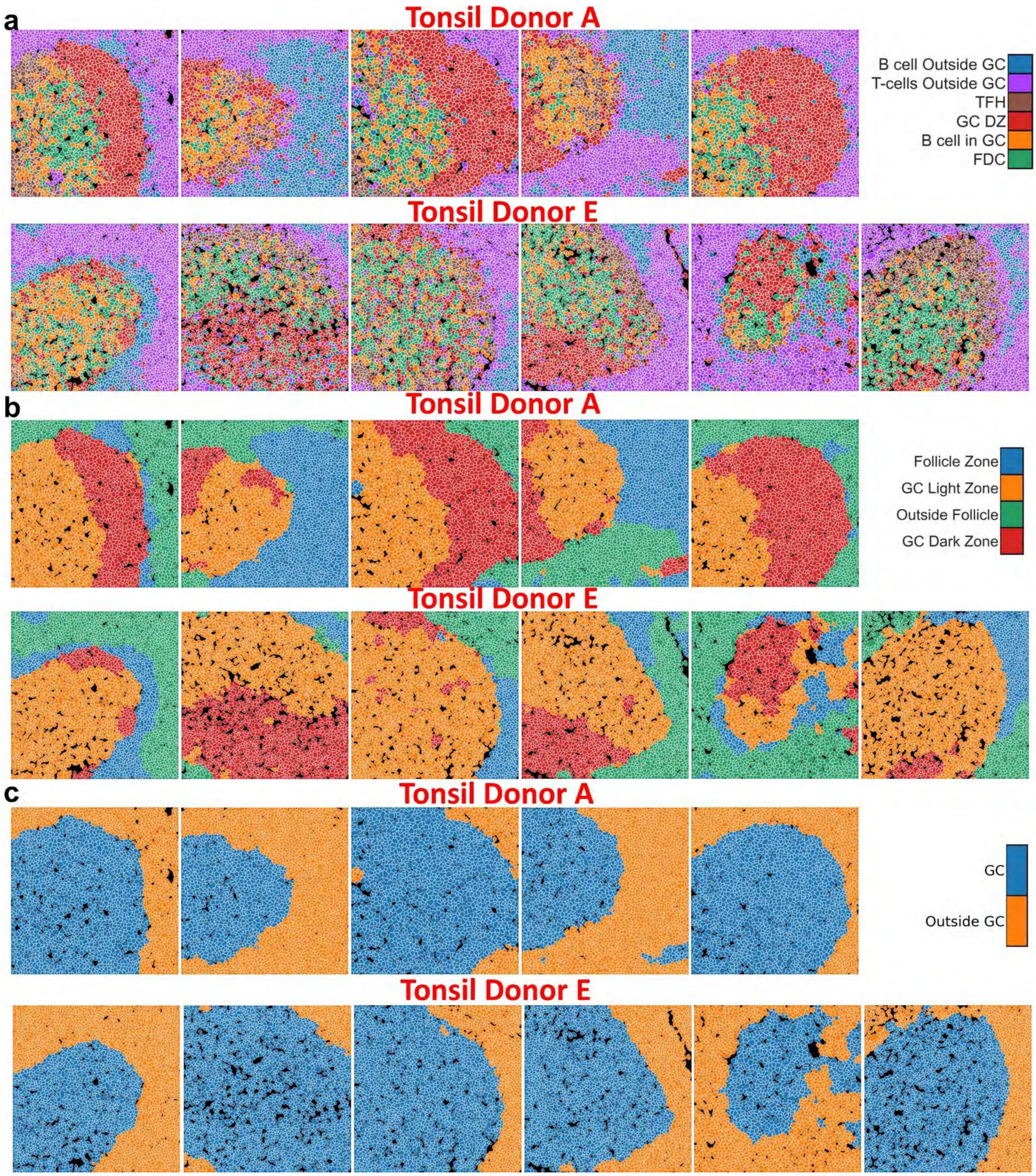
a
Tonsil Donor A
B cell Outside GC
T-cells Outside GC
TFH
GC DZ
B cell in GC
FDC
Tonsil Donor E
b
Tonsil Donor A
Follicle Zone
GC Light Zone
Outside Follicle
GC Dark Zone
Tonsil Donor E
c
Tonsil Donor A
GC
Outside GC
Tonsil Donor E

Supplementary Fig. 28. Single-cell protein phenotyping in human tonsils.

- **a** Spatial projection of unsupervised single-cell clusters from protein profiles in human tonsil tissues for all imaged follicle regions.
- **b** Classification of cells into the follicle, outside follicle, germinal center dark zone, and germinal center light zone regions from cell clusters obtained from a).
- **c** Classification of cells into germinal center dark and non-germinal center regions from cell clusters obtained from a)

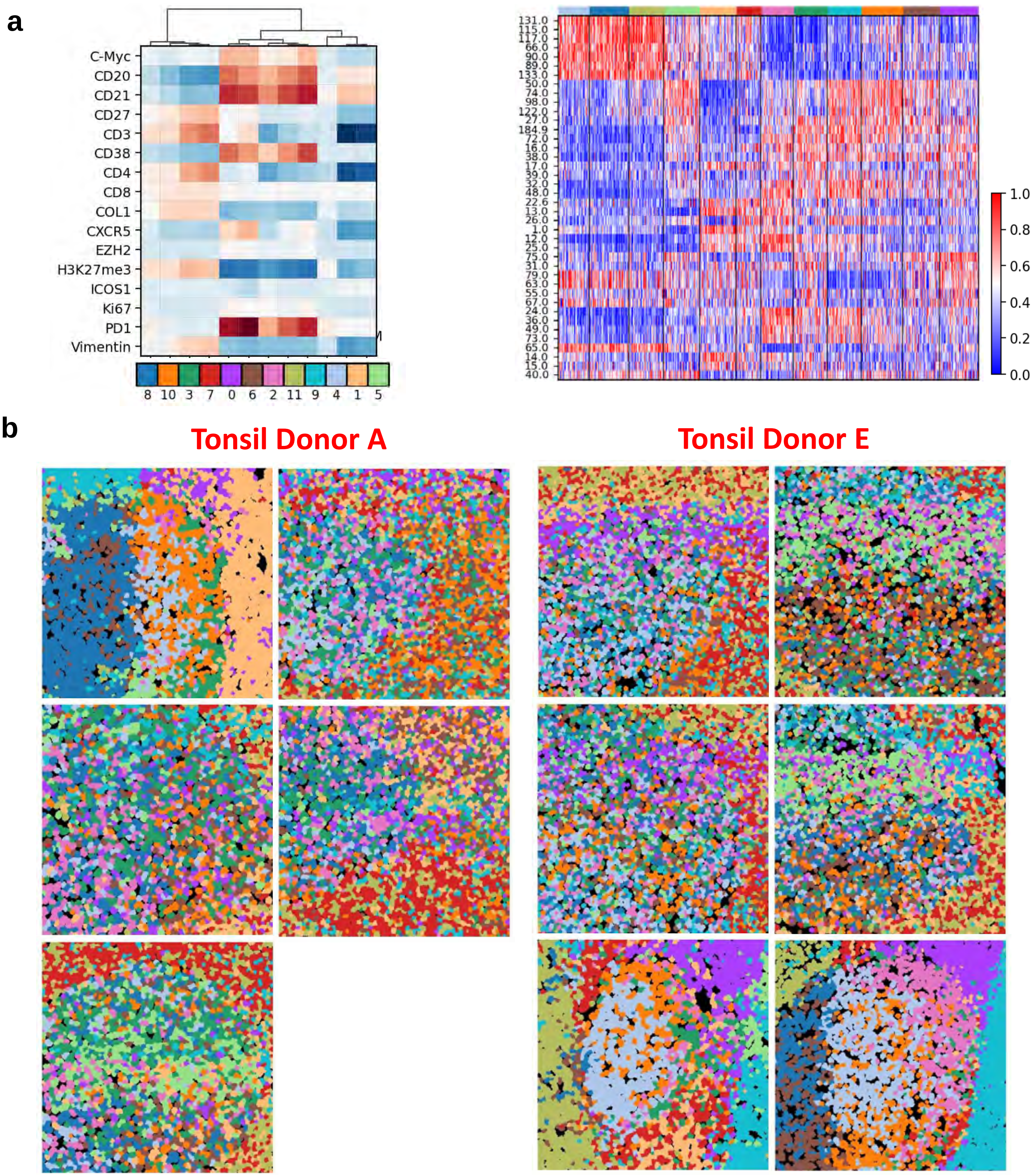

a
C-Myc
CD20
CD21
CD27
CD3
CD38
CD4
CD8
COL1
CXCR5
EZH2
H3K27me3
ICOS1
Ki67
PD1
Vimentin
8 10 3 7 0 6 2 11 9 4 1 5
1.0
0.8
0.6
0.4
0.2
0.0
b
Tonsil Donor A
Tonsil Donor E

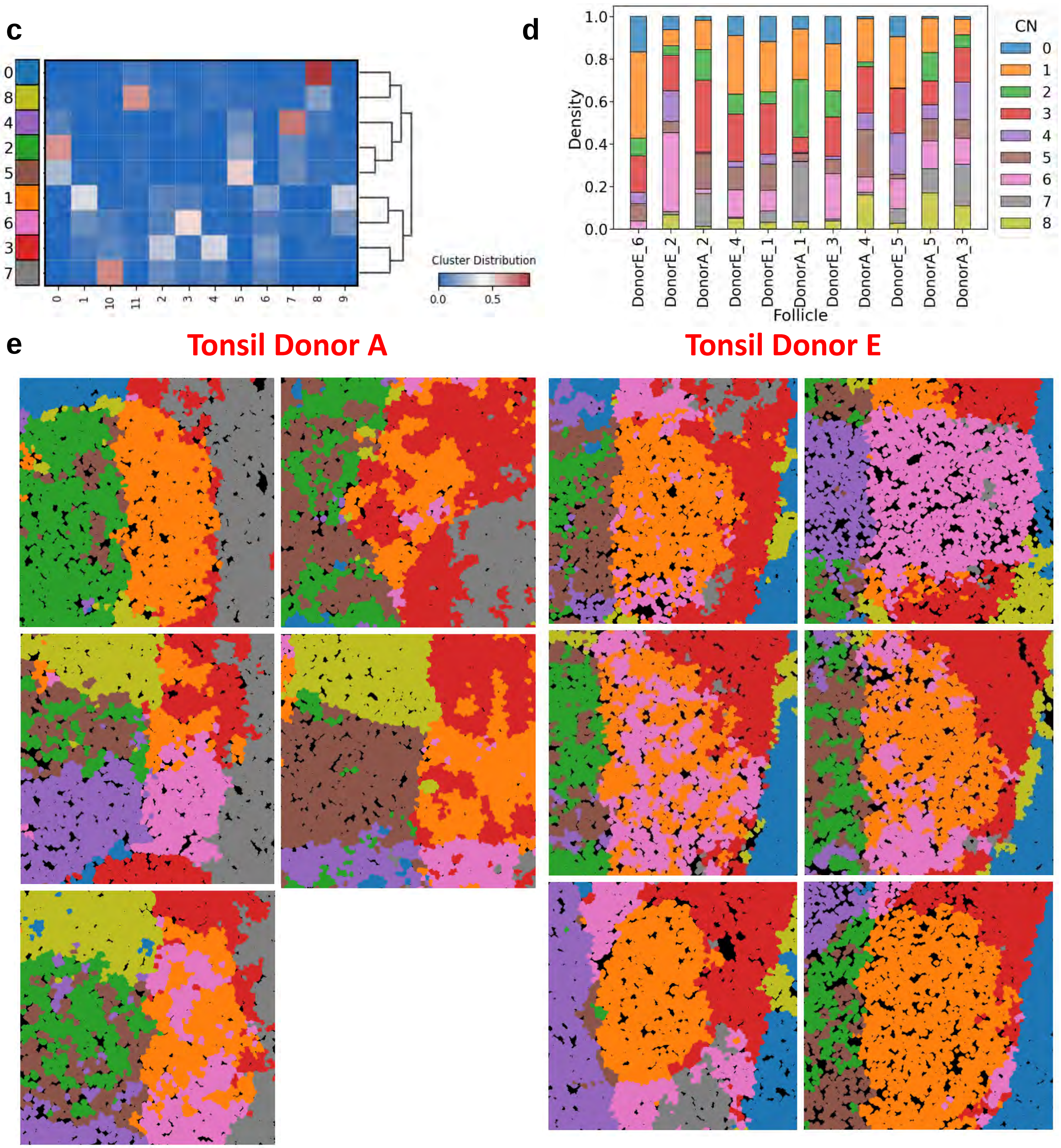

c
0
8
4
2
5
1
6
3
7
0
1
10
11
2
3
4
5
6
7
8
9
Cluster Distribution
0.0
0.5
d
1.0
0.8
0.6
0.4
0.2
0.0
Density
DonorE_6
DonorE_2
DonorA_2
DonorE_4
DonorE_1
DonorA_1
DonorE_3
DonorA_4
DonorE_5
DonorA_5
DonorA_3
Follicle
CN
0
1
2
3
4
5
6
7
8
e
Tonsil Donor A
Tonsil Donor E

Supplementary Fig. 29. Single-cell joint protein-metabolite phenotyping in tonsil follicles (n=31156 cells).

- **a** VAE single cell protein-metabolite joint embedding. Left: architecture for single-cell protein-metabolite joint embedding. Right: joint protein-metabolites single-cell phenotype.
- **b** Spatial projection of clusters obtained from **a** human tonsil tissues for all imaged follicle regions.
- **c** Density of unsupervised cluster in defined cell neighborhoods.
- **d** The density of each cell neighborhood across the follicle imaging regions.
- **e** Spatial projection of cell neighborhood obtained from **c** and **d** in human tonsil tissues for all imaged regions of interest.

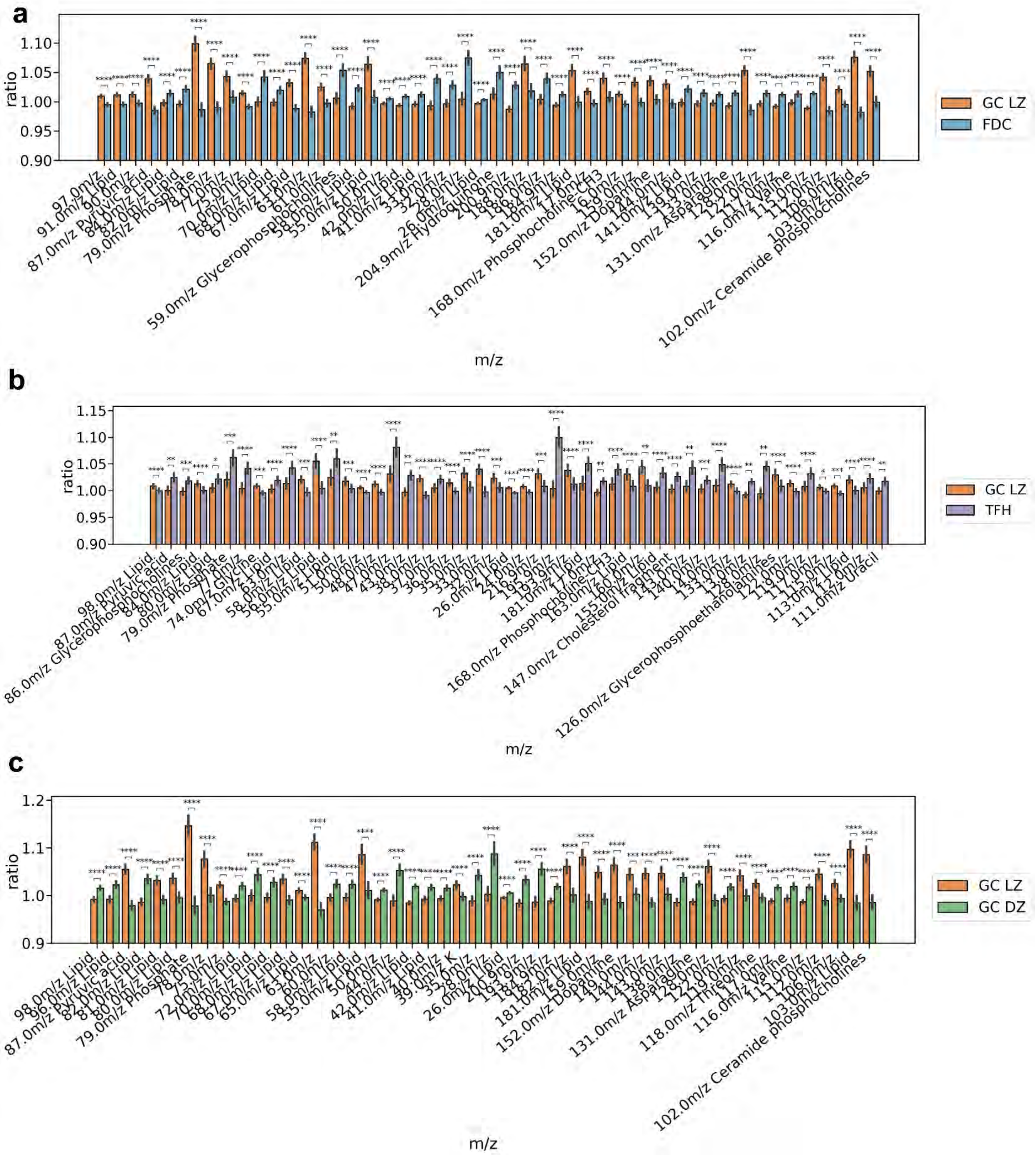

a
ratio
m/z
GC LZ
FDC
59.0m/z Glycerophosphocholines
204.9m/z Hydroquinone
168.0m/z Phosphocholine-CH3
152.0m/z Dopamine
131.0m/z Asparagine
116.0m/z Valine
102.0m/z Ceramide phosphocholines
b
ratio
m/z
GC LZ
TFH
86.0m/z Glycerophosphocholines
168.0m/z Phosphocholine-CH3
147.0m/z Cholesterol fragment
126.0m/z Glycerophosphoethanolamines
111.0m/z Uracil
c
ratio
m/z
GC LZ
GC DZ
87.0m/z Pyruvic acid
79.0m/z Phosphate
152.0m/z Dopamine
131.0m/z Asparagine
118.0m/z Threonine
116.0m/z Valine
102.0m/z Ceramide phosphocholines

Supplementary Fig. 30. Single-cell local metabolite competition in tonsil tissues. Local competition of metabolites between B cells and FDCs (**a**) (n=4371 cells), B cells and TFHs (**b**) (n=2807 cells), B cells in LZ with DZ (**c**) (n=1870 cells). Mann-Whitney-Wilcoxon test was two-sided with Bonferroni correction (ns: 0.05 < p, ****: p<=0.0001).

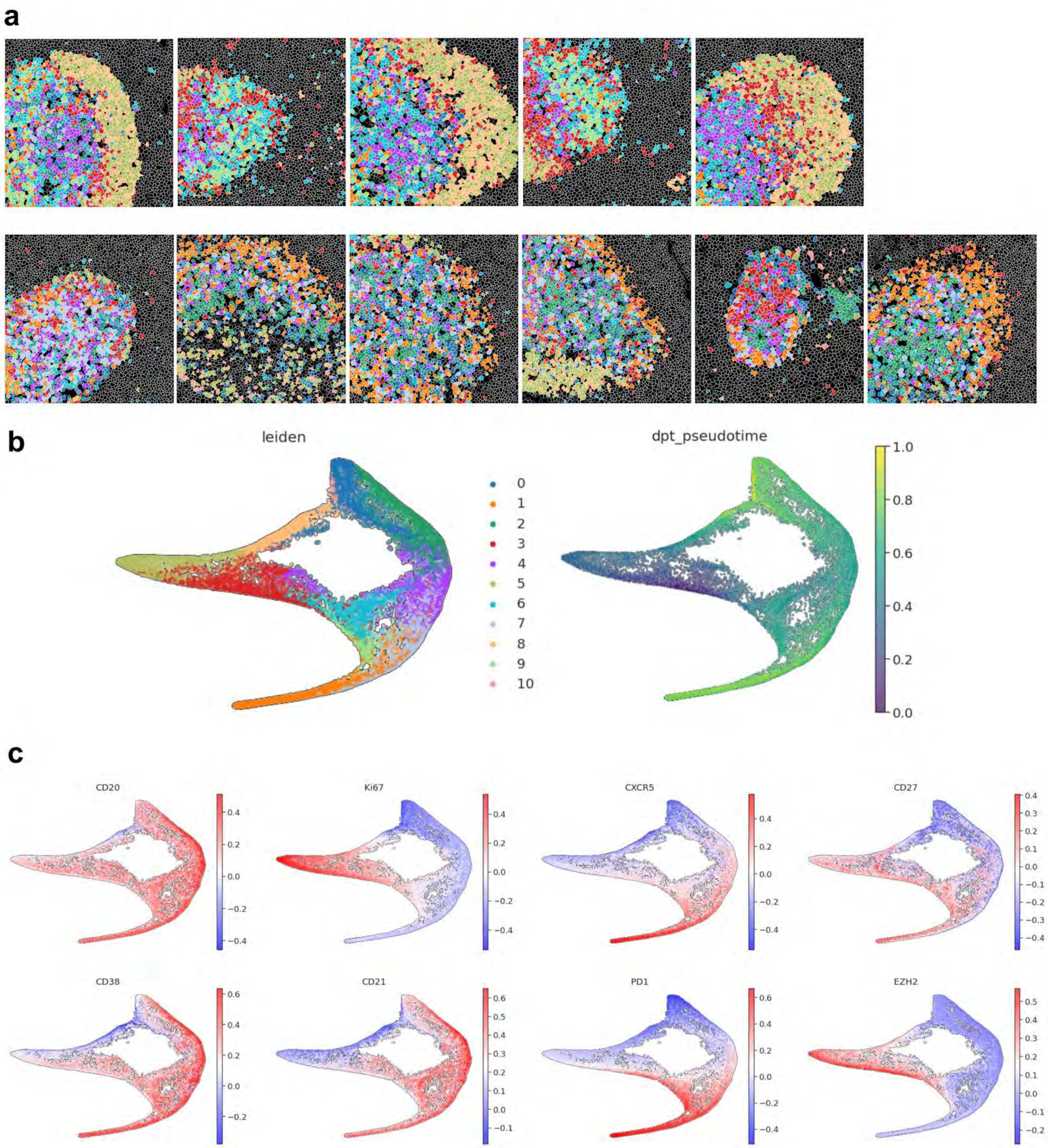
a
b
leiden
0
1
2
3
4
5
6
7
8
9
10
dpt_pseudotime
1.0
0.8
0.6
0.4
0.2
0.0
c
CD20
Ki67
CXCR5
CD27
CD38
CD21
PD1
EZH2

Supplementary Fig. 31. B-cell trajectory inside germinal centers (n=31156 cells).

a Spatial projection of selected B-cell clusters from protein profiles in human tonsil tissues for all imaged follicle regions.

b Pseudotime analysis of B-cells. Left: t-SNE embedding and unsupervised clustering of B-cells. Right: Pseudotime analysis.

c Projection of single-cell marker intensity on t-SNE map.

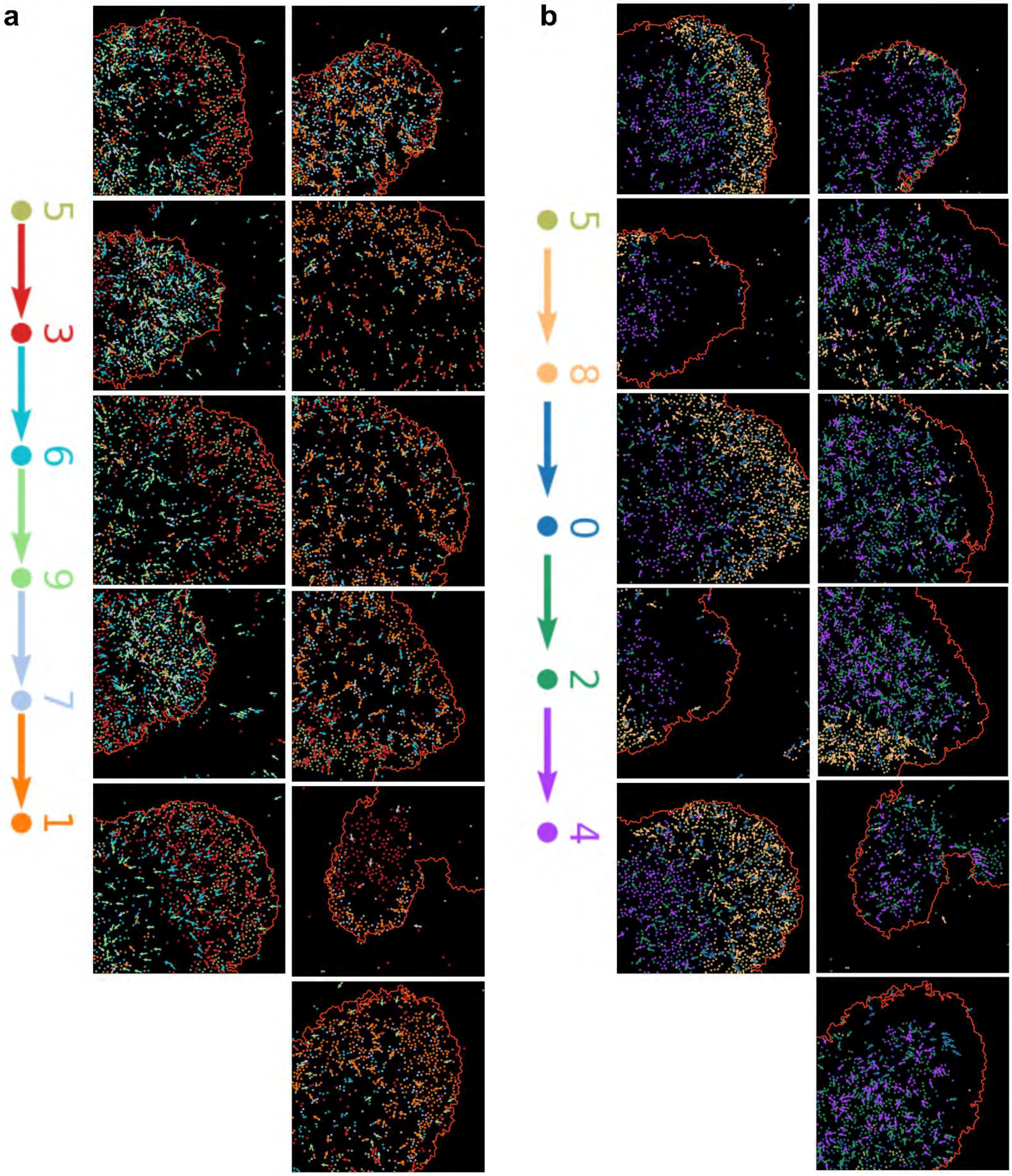
a
5
3
6
9
7
1
b
5
8
0
2
4

Supplementary Fig. 32. Spatial projection of B-cell trajectory inside germinal centers.

a Spatial projection of selected B-cell pseudotime differentiation analysis from the germinal center dark zone to the germinal center light zone. Left: Arrows showing the trajectory along each cell cluster. Right: Spatial projection of single cells. Schematics of arrows and colors were created with Biorender.com.

b Spatial projection of selected B-cell pseudotime differentiation analysis from germinal center dark zone to activated B-cells. Left: Arrows showing the trajectory along each cell cluster. Right: Spatial projection of single cells. Schematics of arrows and colors were created with Biorender.com.

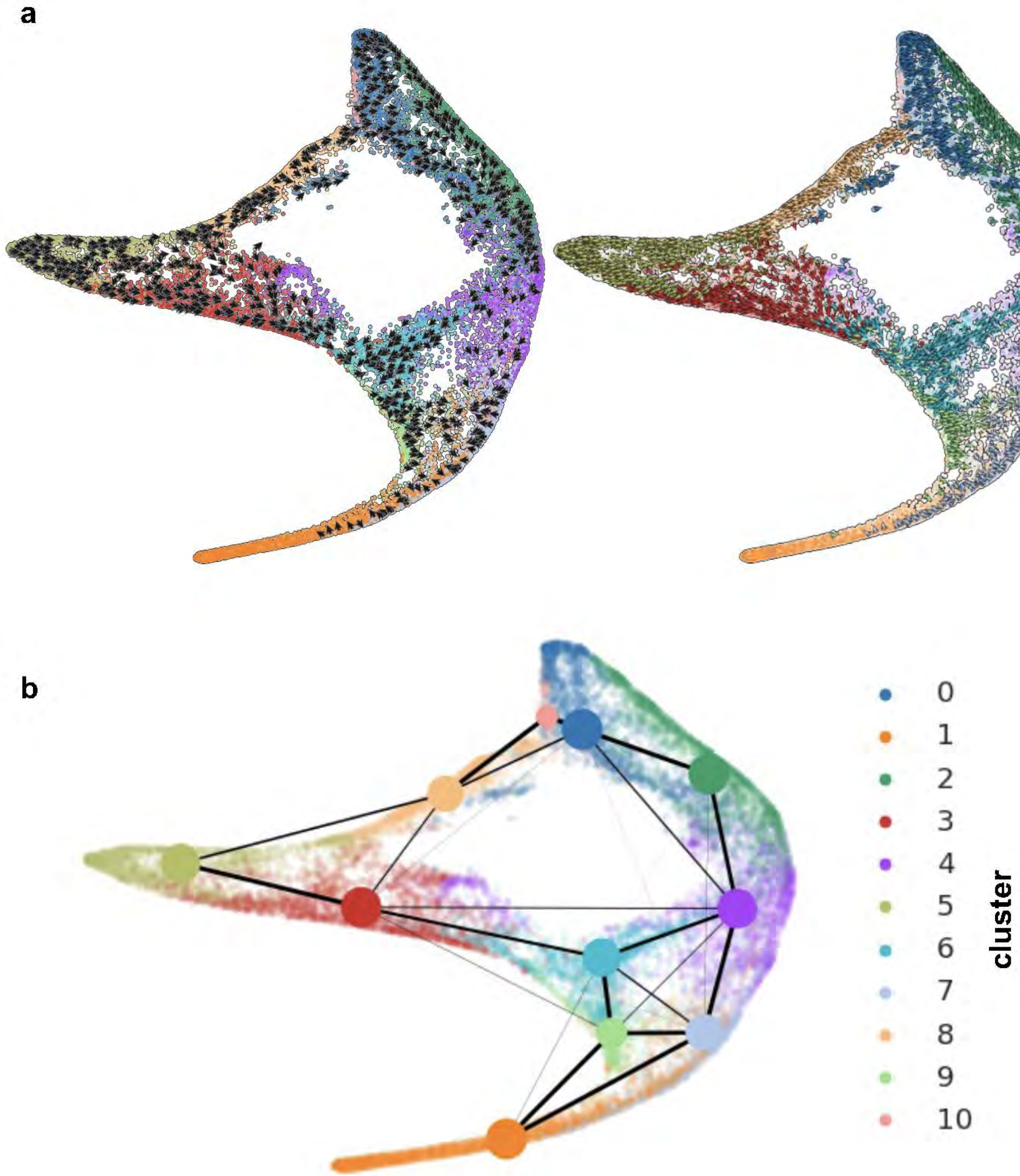
a
b
0
1
2
3
4
5
6
7
8
9
10
cluster

Supplementary Fig. 33. Projection of B-cell trajectory on t-SNE.

a Single-cell t-SNE embedding showing the trajectory of B-cells inside germinal center regions. Arrows showing the trajectory along each cell cluster.

b The graph-directed plot of B-cell embedding on t-SNE.

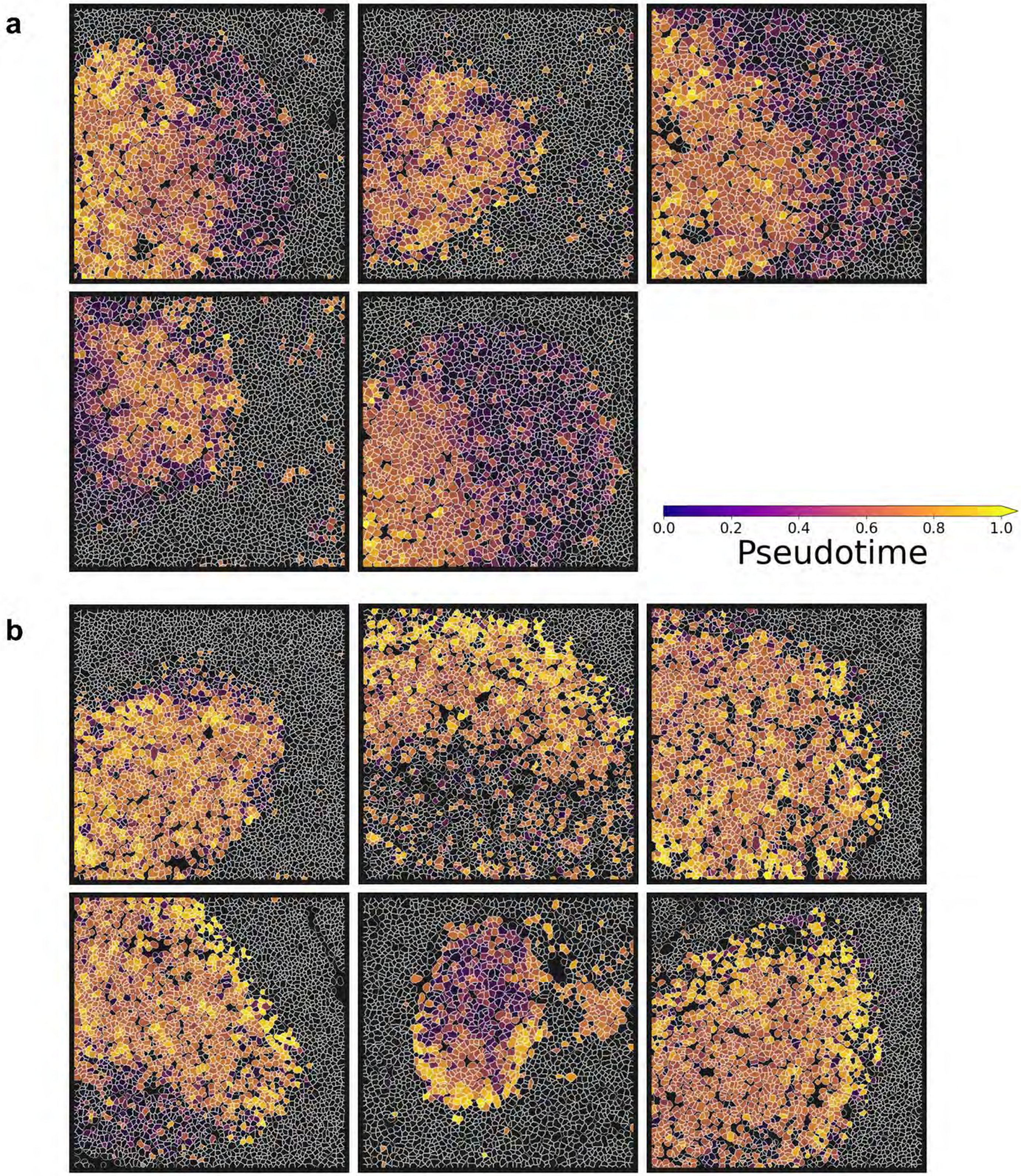
a
b
0.0
0.2
0.4
0.6
0.8
1.0
Pseudotime

Supplementary Fig. 34. Spatial projection of B-cell trajectory inside germinal centers. Spatial projection of single-cell with corresponding pseudotime value from pseudotime analysis in tonsil donor A **a** and tonsil donor B **b**.

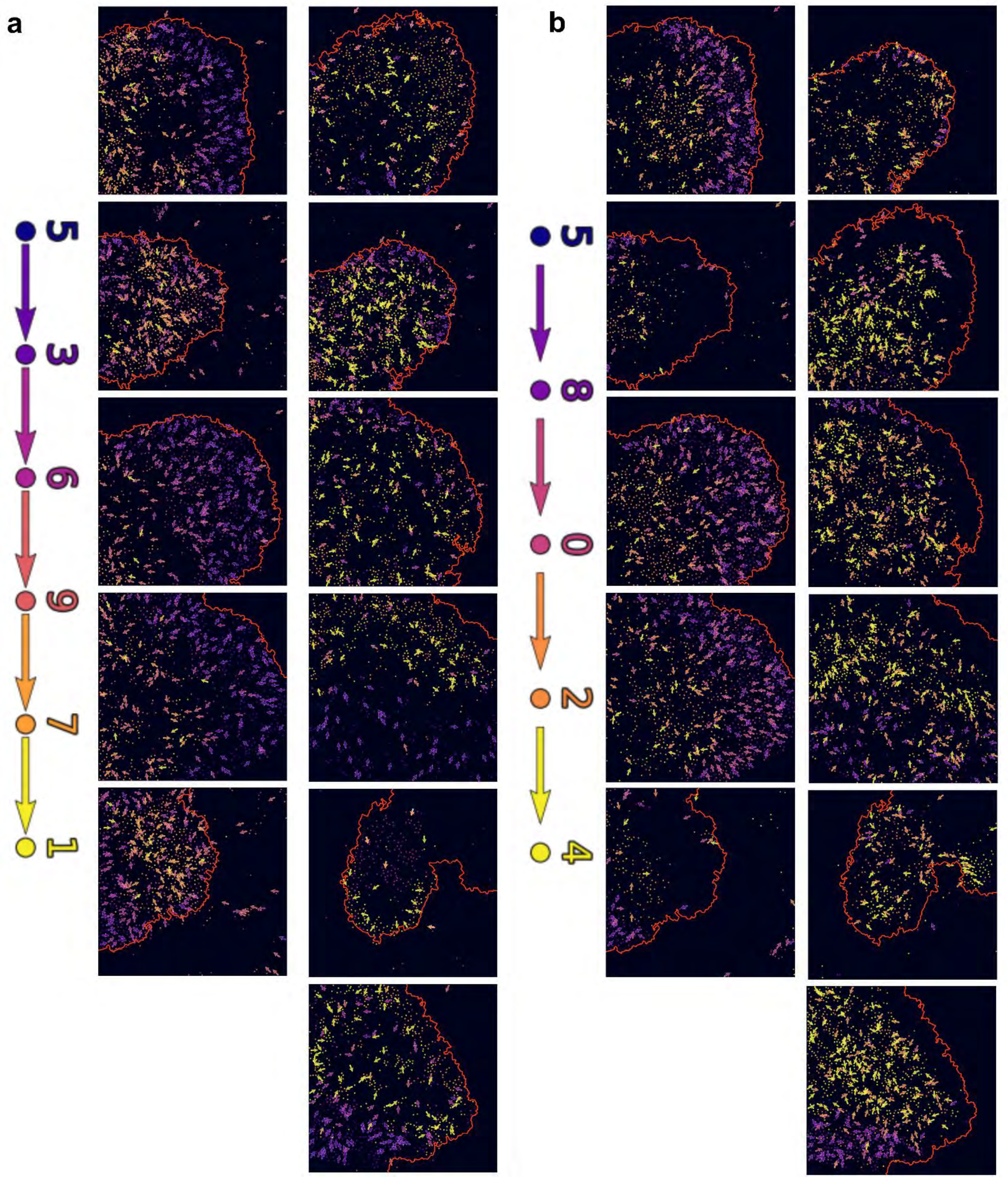
a
5
3
6
9
7
1
b
5
8
0
2
4

Supplementary Fig. 35. Spatial projection of B-cell trajectory inside germinal centers.

a Spatial projection of selected B-cell pseudotime differentiation analysis from the germinal center dark zone to the germinal center light zone. Left: Arrows showing the trajectory along each cell cluster with corresponding pseudotime value. Right: Spatial projection of single cells with corresponding pseudotime value. Schematics of arrows and colors were created with Biorender.com.

b Spatial projection of selected B-cell pseudotime differentiation analysis from germinal center dark zone to activated B-cells. Left: Arrows showing the trajectory along each cell cluster with corresponding pseudotime value. Right: Spatial projection of single cells with corresponding pseudotime value. Schematics of arrows and colors were created with Biorender.com.

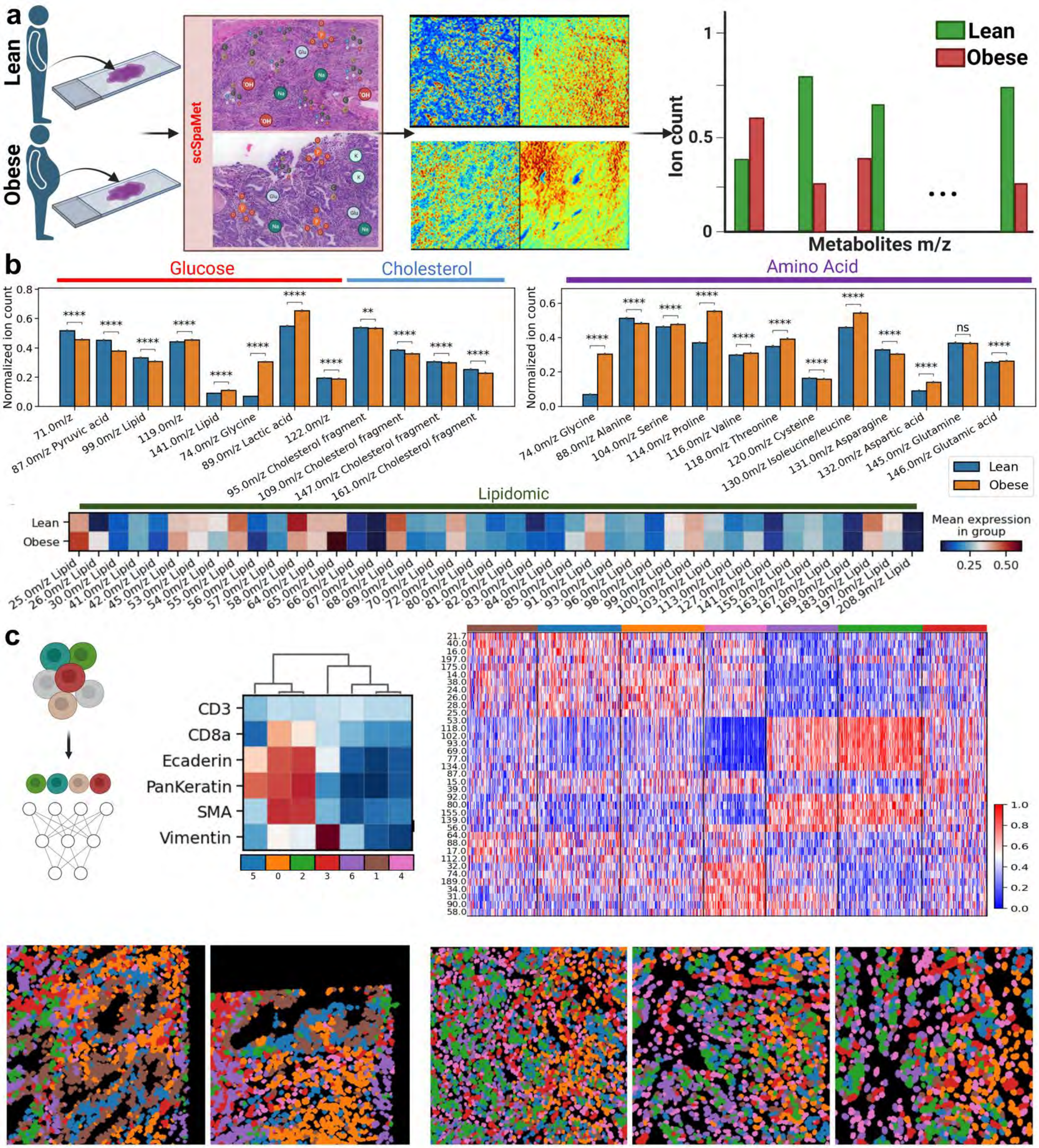

a
Lean
Obese
scSpaMet
Ion count
1
0.5
0
Metabolites m/z
Lean
Obese
b
Glucose
Cholesterol
Amino Acid
Normalized ion count
71.0m/z
87.0m/z Pyruvic acid
99.0m/z Lipid
119.0m/z
141.0m/z Lipid
74.0m/z Glycine
89.0m/z Lactic acid
122.0m/z
95.0m/z Cholesterol fragment
109.0m/z Cholesterol fragment
147.0m/z Cholesterol fragment
161.0m/z Cholesterol fragment
74.0m/z Glycine
88.0m/z Alanine
104.0m/z Serine
114.0m/z Proline
116.0m/z Valine
118.0m/z Threonine
120.0m/z Cysteine
130.0m/z Isoleucine/leucine
131.0m/z Asparagine
132.0m/z Aspartic acid
145.0m/z Glutamine
146.0m/z Glutamic acid
ns
Lean
Obese
Lipidomic
Mean expression in group
0.25 0.50
c
CD3
CD8a
Ecaderin
PanKeratin
SMA
Vimentin
5 0 2 3 6 1 4

Supplementary Fig. 36. ScSpaMet quantifies metabolite differences of lean and obese patients in human endometrium samples.

a Representative schematic showing the comparison of metabolite expression in lean and obese samples from human endometrium. Created with Biorender.com.

b Comparison of single cell metabolic expression level for identified metabolite channels between lean and obese samples (n=8215 cells). Left: metabolite channels related to Glucose pathway and Cholesterol fragments. Right: Metabolite channels related to amino acid fragments. Mann-Whitney-Wilcoxon test was two-sided with Bonferroni correction (ns: 0.05 < p, ****: p<=0.0001).

c Metabolite channels related to identified lipid channels fragmentation.

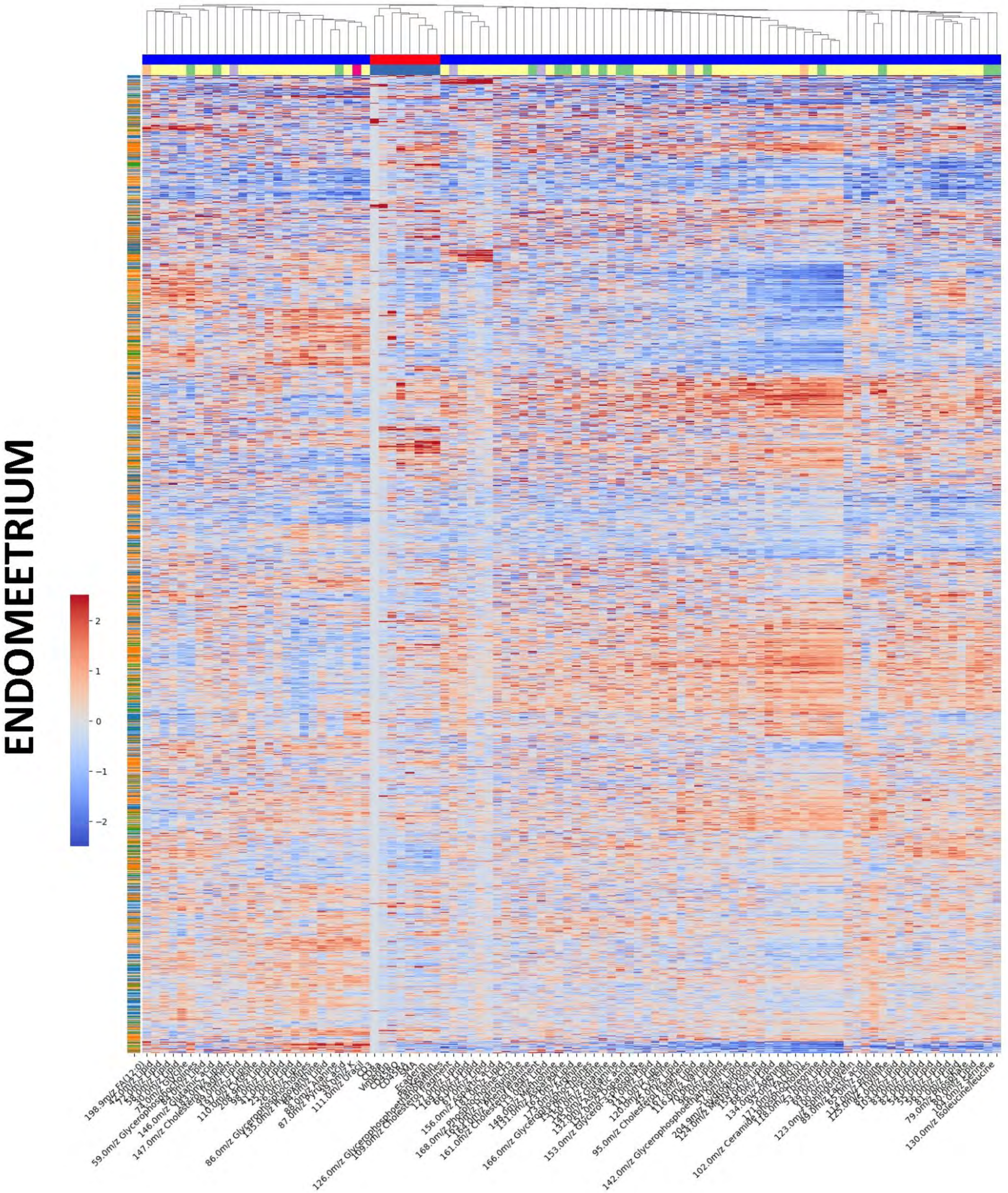

ENDOMEETRIUM
2
1
0
−1
−2

Supplementary Fig. 37. Single-cell metabolite and protein profile in endometrium tissues (n=8215 cells). Cluster map showing the cell-level metabolite and protein profile in all endometrium tissues. Colorbar corresponds to the mean intensity value at the single cell level.

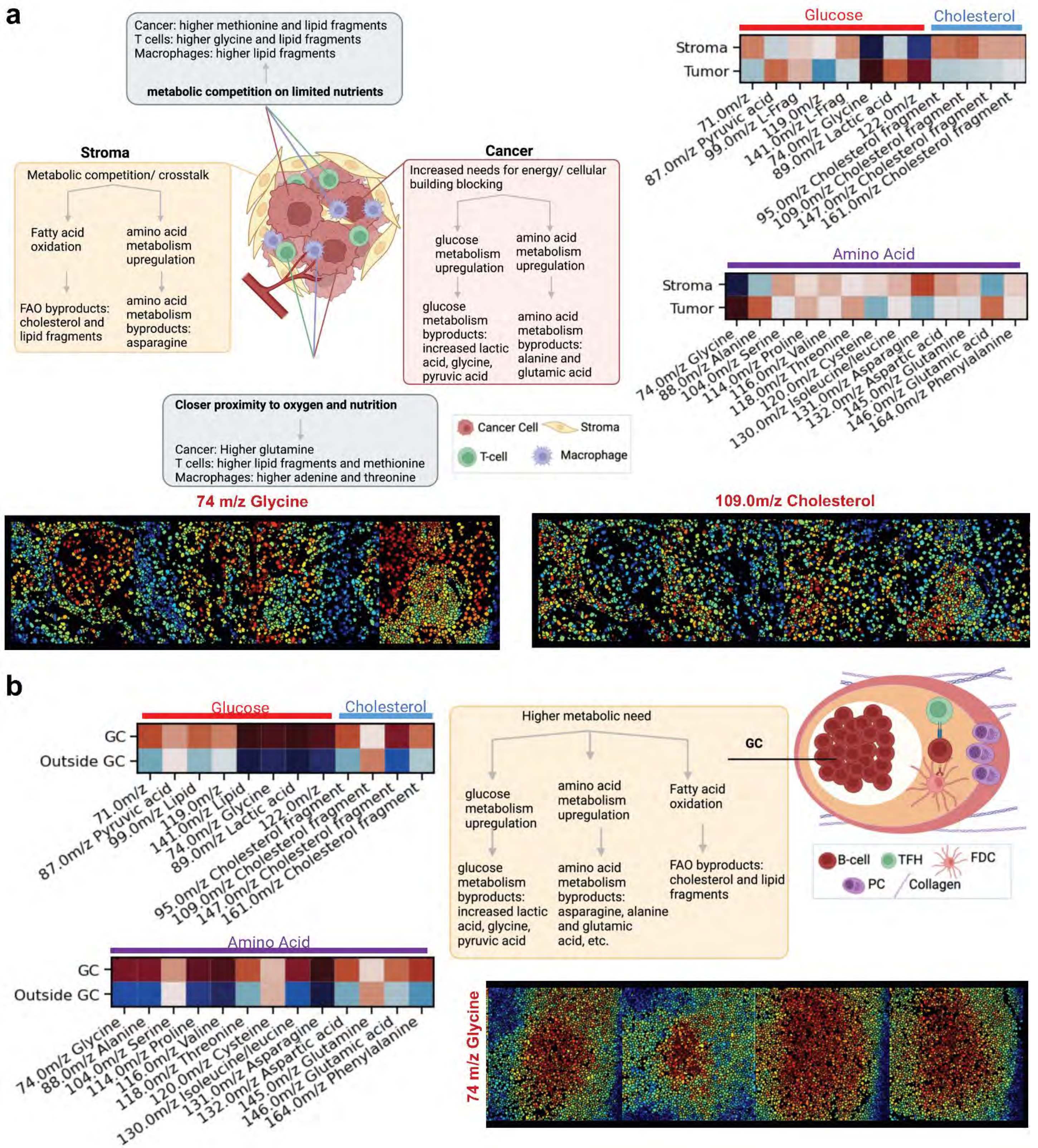
a
Cancer: higher methionine and lipid fragments
T cells: higher glycine and lipid fragments
Macrophages: higher lipid fragments
metabolic competition on limited nutrients
Stroma
Metabolic competition/ crosstalk
Fatty acid oxidation
amino acid metabolism upregulation
FAO byproducts: cholesterol and lipid fragments
amino acid metabolism byproducts: asparagine
Cancer
Increased needs for energy/ cellular building blocking
glucose metabolism upregulation
amino acid metabolism upregulation
glucose metabolism byproducts: increased lactic acid, glycine, pyruvic acid
amino acid metabolism byproducts: alanine and glutamic acid
Closer proximity to oxygen and nutrition
Cancer: Higher glutamine
T cells: higher lipid fragments and methionine
Macrophages: higher adenine and threonine
Cancer Cell
Stroma
T-cell
Macrophage
Glucose
Cholesterol
Stroma
Tumor
71.0m/z
87.0m/z Pyruvic acid
99.0m/z L-Frag
119.0m/z
141.0m/z L-Frag
74.0m/z Glycine
89.0m/z Lactic acid
122.0m/z
95.0m/z Cholesterol fragment
109.0m/z Cholesterol fragment
147.0m/z Cholesterol fragment
161.0m/z Cholesterol fragment
Amino Acid
Stroma
Tumor
74.0m/z Glycine
88.0m/z Alanine
104.0m/z Serine
114.0m/z Proline
116.0m/z Valine
118.0m/z Threonine
120.0m/z Cysteine
130.0m/z Isoleucine/leucine
131.0m/z Asparagine
132.0m/z Aspartic acid
145.0m/z Glutamine
146.0m/z Glutamic acid
164.0m/z Phenylalanine
74 m/z Glycine
109.0m/z Cholesterol
b
Glucose
Cholesterol
GC
Outside GC
71.0m/z
87.0m/z Pyruvic acid
99.0m/z Lipid
119.0m/z
141.0m/z Lipid
74.0m/z Glycine
89.0m/z Lactic acid
122.0m/z
95.0m/z Cholesterol fragment
109.0m/z Cholesterol fragment
147.0m/z Cholesterol fragment
161.0m/z Cholesterol fragment
Amino Acid
GC
Outside GC
74.0m/z Glycine
88.0m/z Alanine
104.0m/z Serine
114.0m/z Proline
116.0m/z Valine
118.0m/z Threonine
120.0m/z Cysteine
130.0m/z Isoleucine/leucine
131.0m/z Asparagine
132.0m/z Aspartic acid
145.0m/z Glutamine
146.0m/z Glutamic acid
164.0m/z Phenylalanine
Higher metabolic need
glucose metabolism upregulation
amino acid metabolism upregulation
Fatty acid oxidation
glucose metabolism byproducts: increased lactic acid, glycine, pyruvic acid
amino acid metabolism byproducts: asparagine, alanine and glutamic acid, etc.
FAO byproducts: cholesterol and lipid fragments
GC
B-cell
TFH
FDC
PC
Collagen
74 m/z Glycine

Supplementary Fig. 38. Summary of findings from joint proteomic and metabolomic analysis from our data in **a** lung cancer tissues and **b** tonsil tissues at the single cell level. Created with Biorender.com

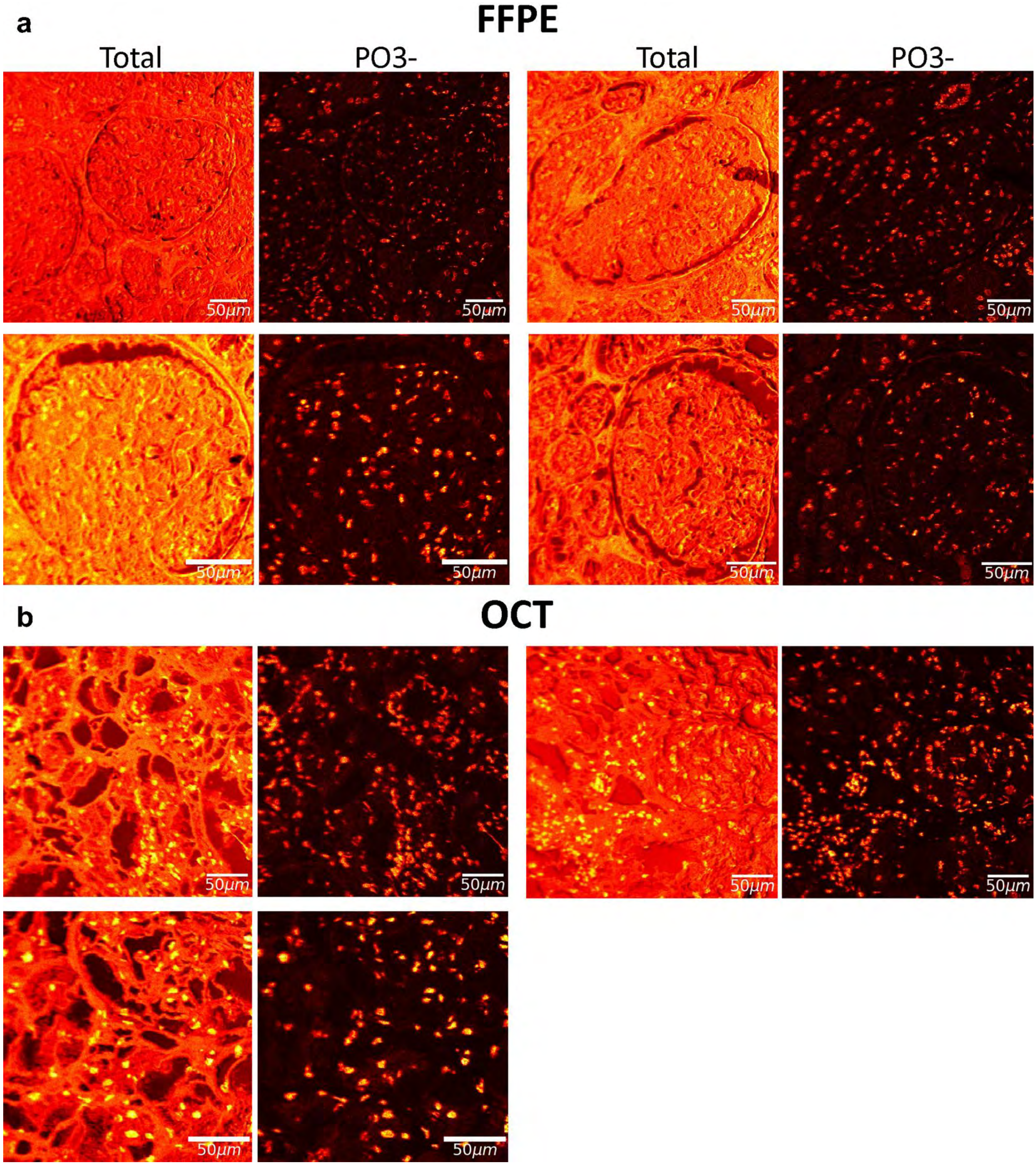
a
FFPE
Total
PO3-
Total
PO3-
50µm
b
OCT

Supplementary Fig. 39. Comparison of TOF–SIMS imaging for **a** FFPE and **b** OCT kidney tissue samples. Scale bar 50 μm.

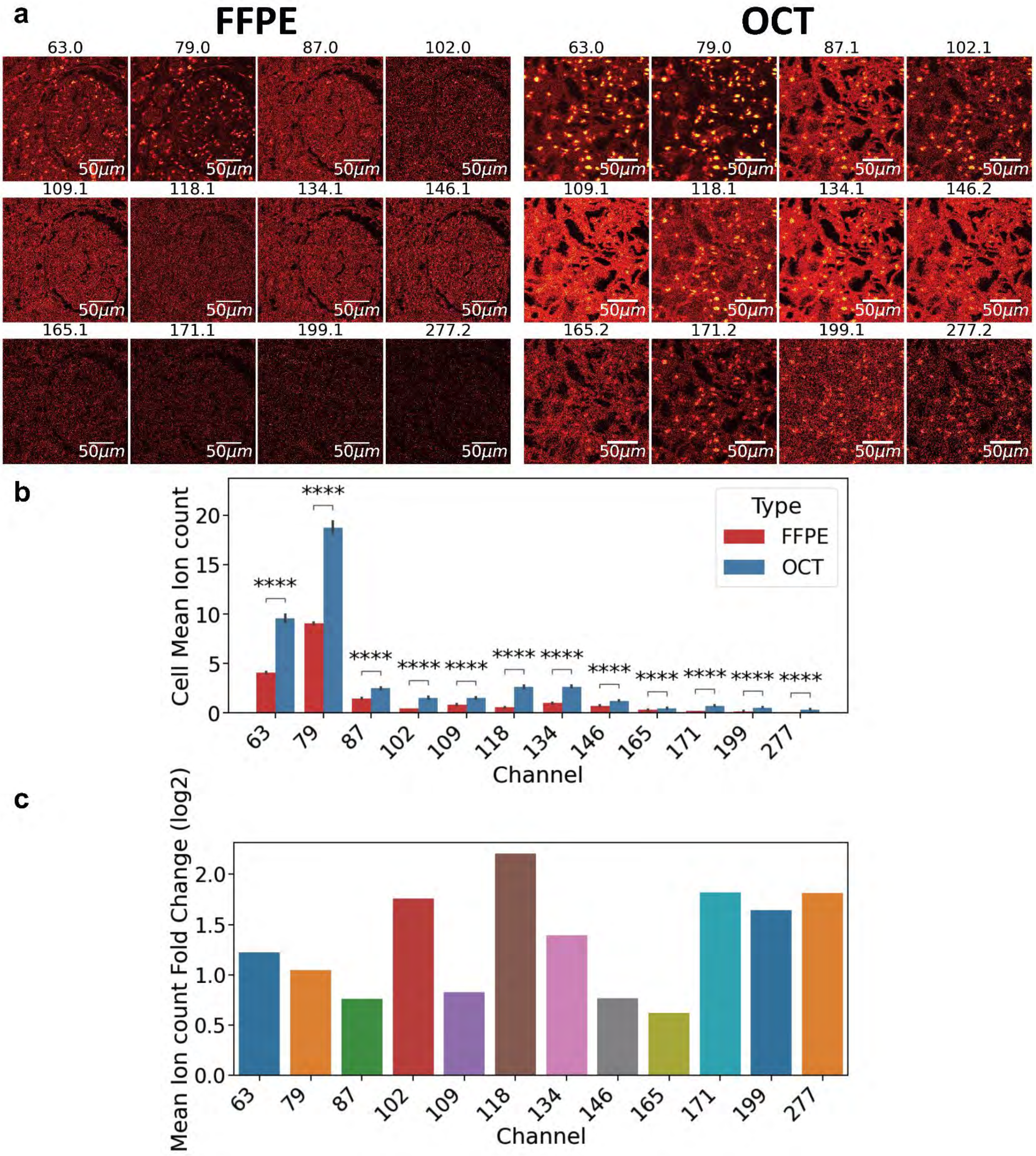
a
FFPE
OCT
63.0
79.0
87.0
102.0
87.1
102.1
109.1
118.1
134.1
146.1
146.2
165.1
171.1
199.1
277.2
165.2
171.2
50μm
b
Cell Mean Ion count
Type
FFPE
OCT
****
Channel
63
79
87
102
109
118
134
146
165
171
199
277
c
Mean Ion count Fold Change (log2)
Channel

Supplementary Fig. 40. Comparison of TOF–SIMS imaging for FFPE (n = 41 cells) and OCT kidney tissue (n = 35 cells) samples in selected channels **a**, mean ion count per cell **b**, and mean ion count fold change **c**. Mann-Whitney-Wilcoxon test was two-sided with Bonferroni correction (****: p<=0.0001). Scale bar 50 µm.

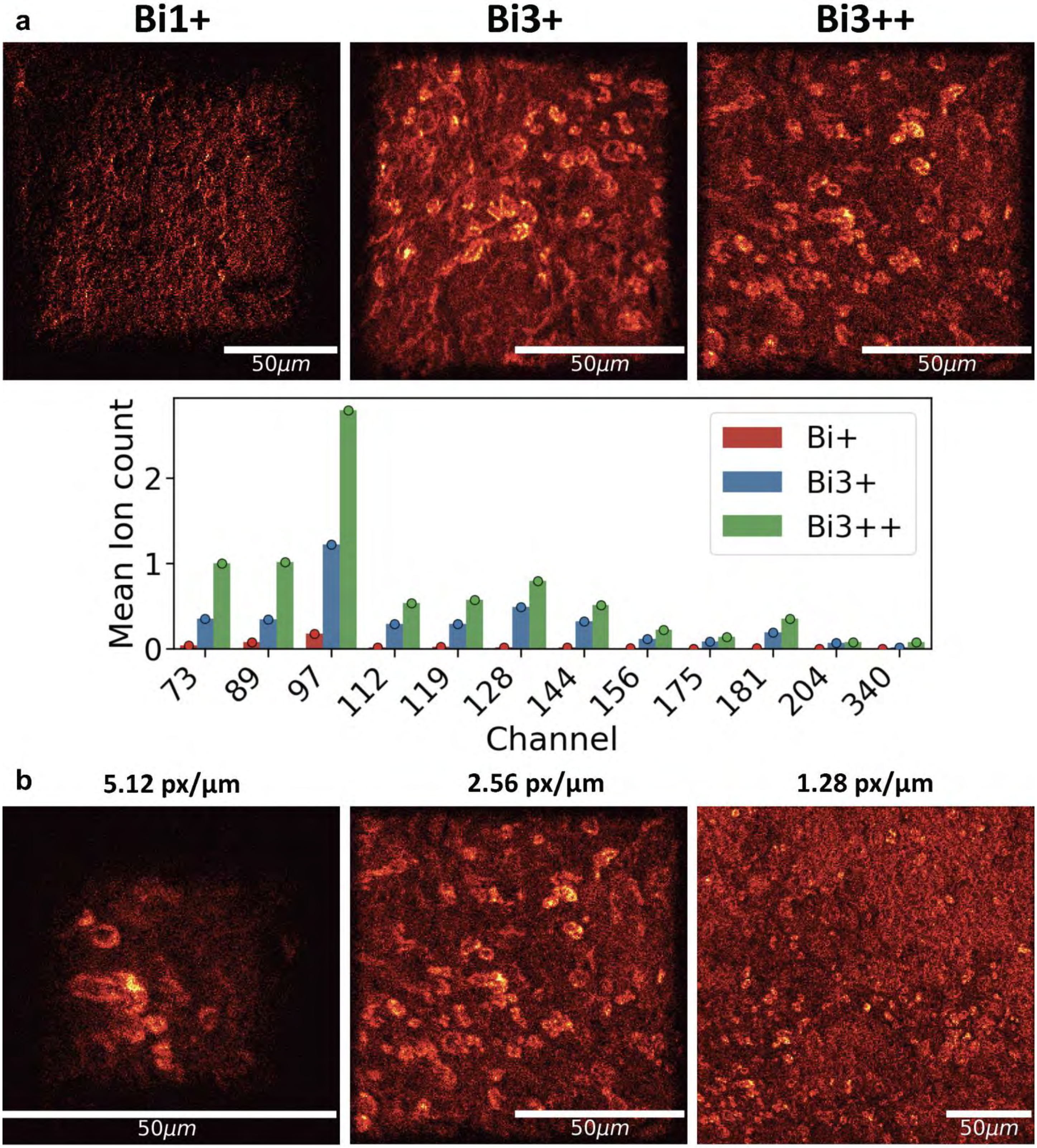
a
Bi1+
Bi3+
Bi3++
50μm
50μm
50μm
Mean Ion count
Bi+
Bi3+
Bi3++
0
1
2
73
89
97
112
119
128
144
156
175
181
204
340
Channel
b
5.12 px/μm
2.56 px/μm
1.28 px/μm
50μm
50μm
50μm

Supplementary Fig. 41. Comparison of TOF-SIMS imaging setting on lung cancer tissues. **a** Comparison of the three TOF-SIMS bismuth liquid metal ion gun modes (Bi+, Bi3+, Bi3++) for extracting single-cell features. Bar plot showing the mean ion count per pixel in selected channels. **b** Comparison of imaging regions size (50µm, 100µm, 200µm) at 256x256 pixels density. Scale bar 50 µm.

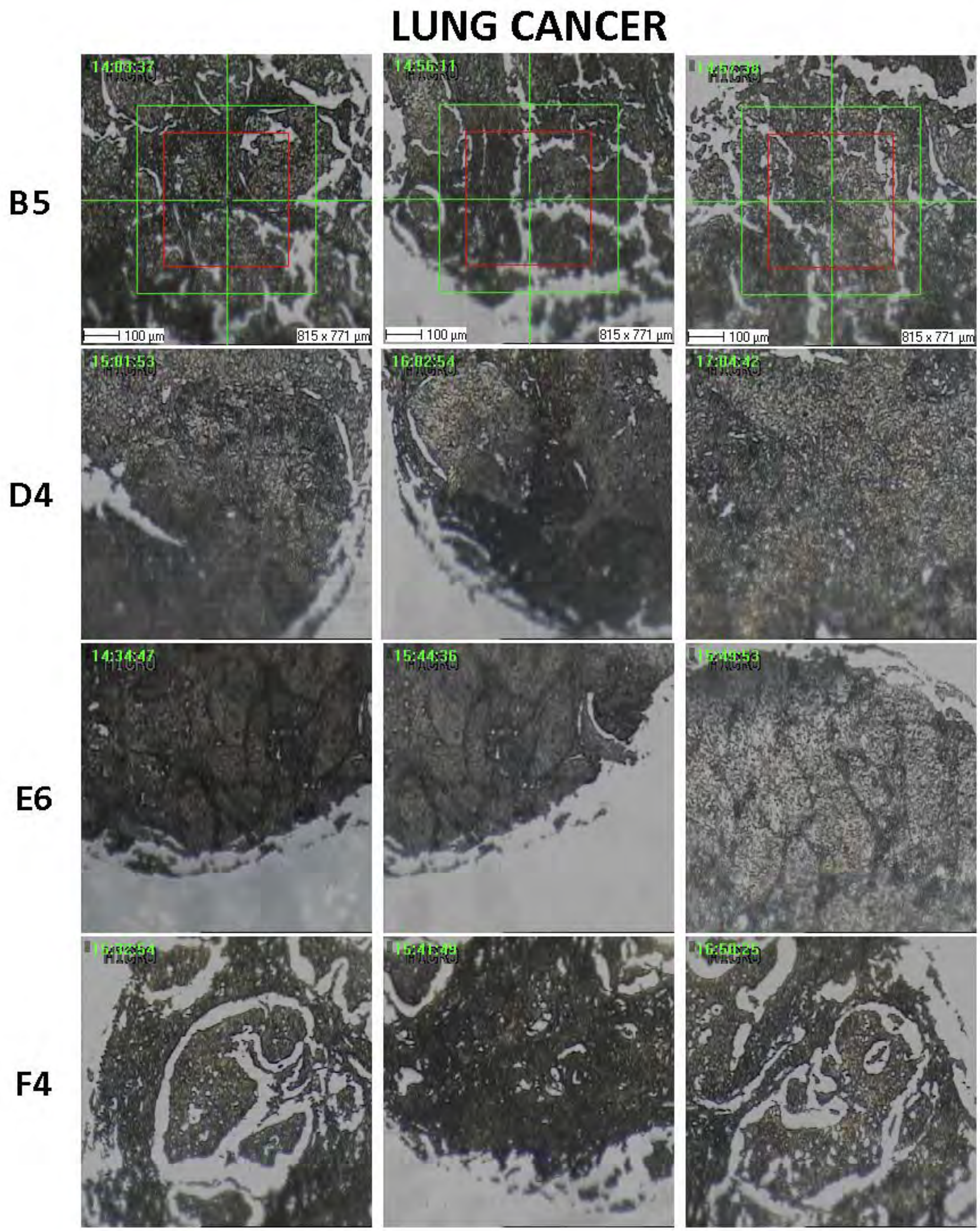

LUNG CANCER
B5
D4
E6
F4
100 µm
815 x 771 µm

Supplementary Fig. 42. Bright field imaging from TOF-SIMS showing tissue regions in human lung cancer tissues.

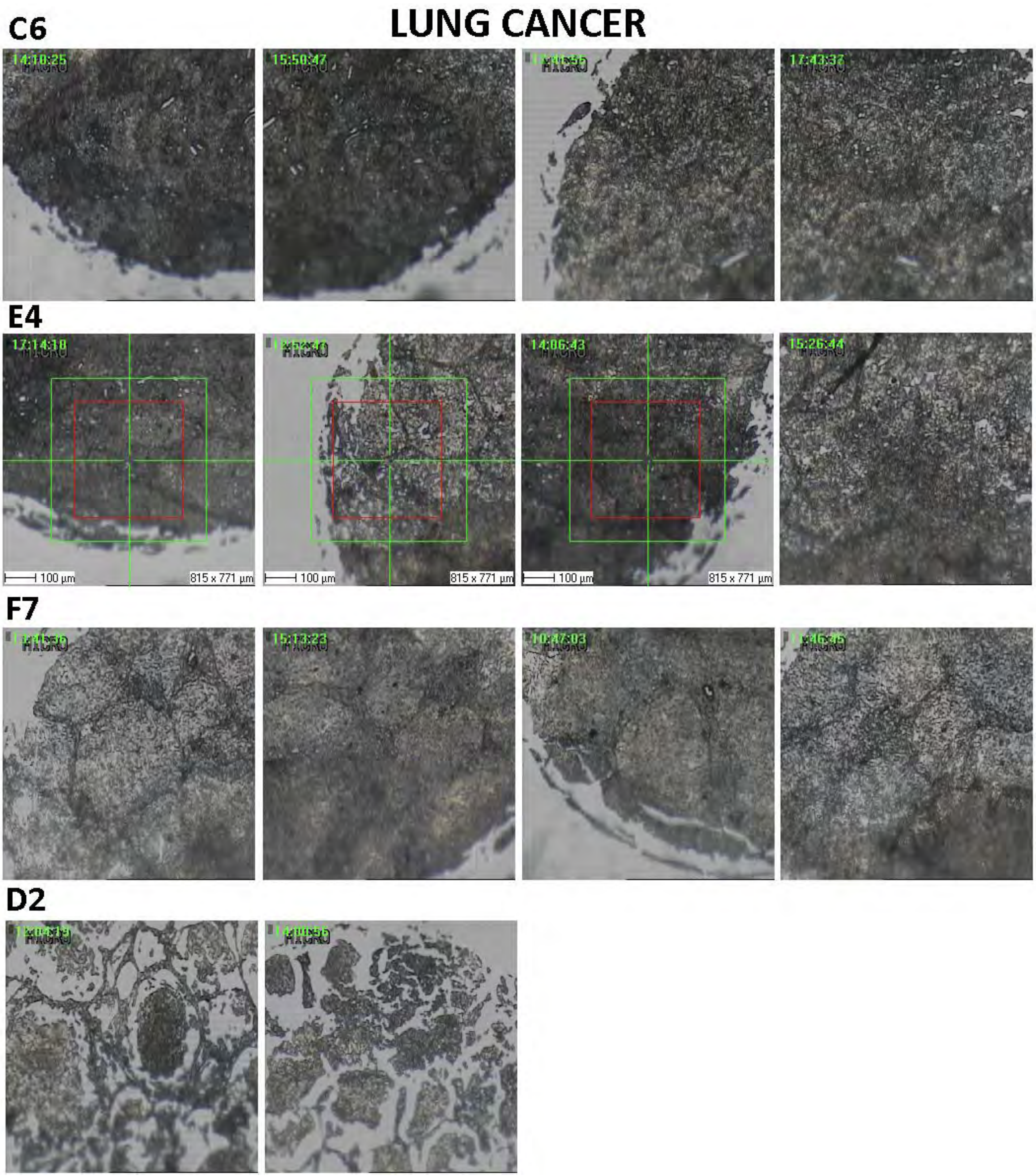
LUNG CANCER
C6
14:10:25
15:50:47
17:43:37
E4
17:14:18
14:06:43
15:26:44
100 µm
815 x 771 µm
F7
15:13:23
10:47:03
D2

Supplementary Fig. 43. Bright field imaging from TOF-SIMS showing tissue regions in human lung cancer tissues.

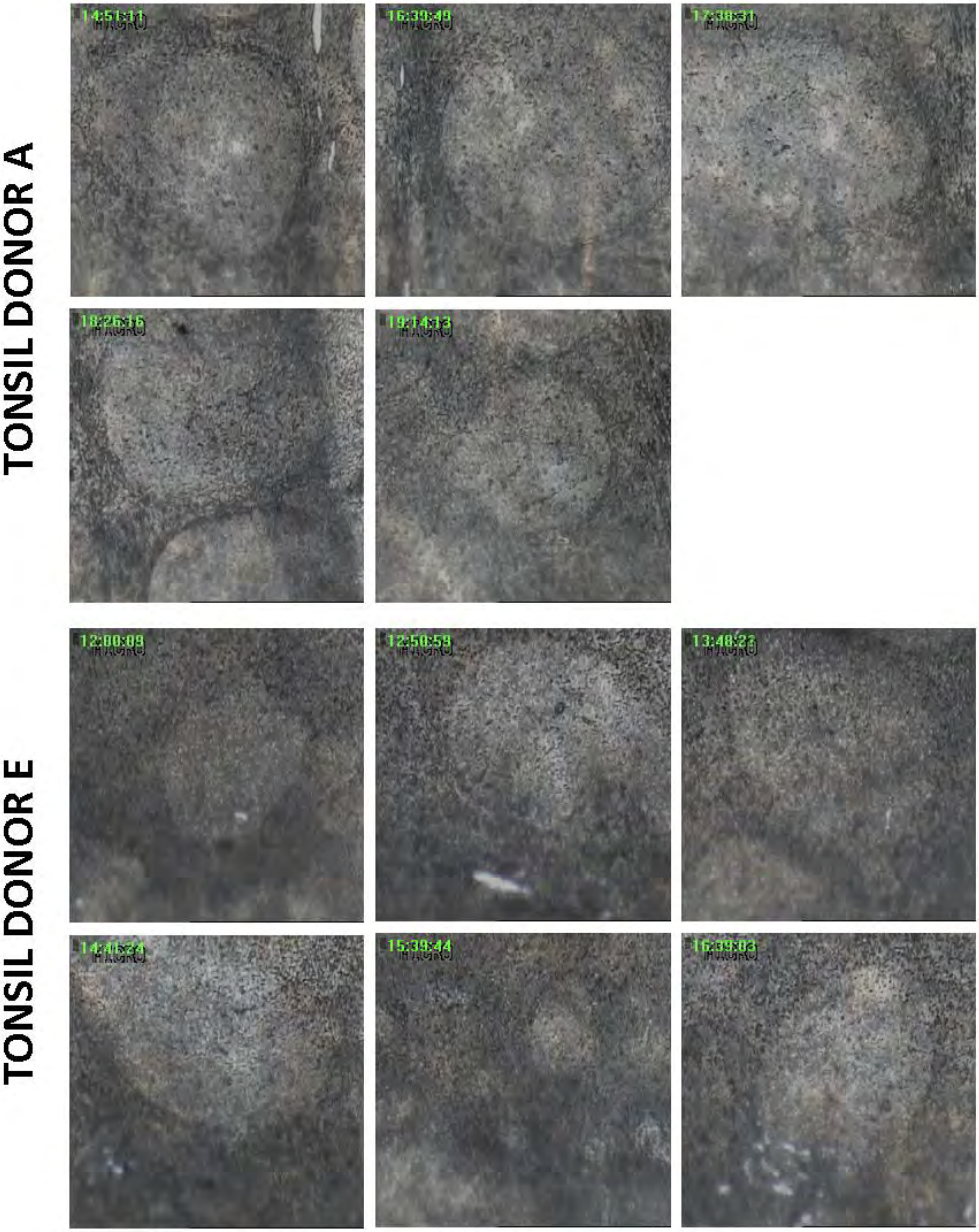
TONSIL DONOR A
14:51:11
16:39:49
18:26:16
19:14:13
TONSIL DONOR E
12:50:59
15:39:44

Supplementary Fig. 44. Bright field imaging from TOF-SIMS showing tissue regions in human tonsil tissues.

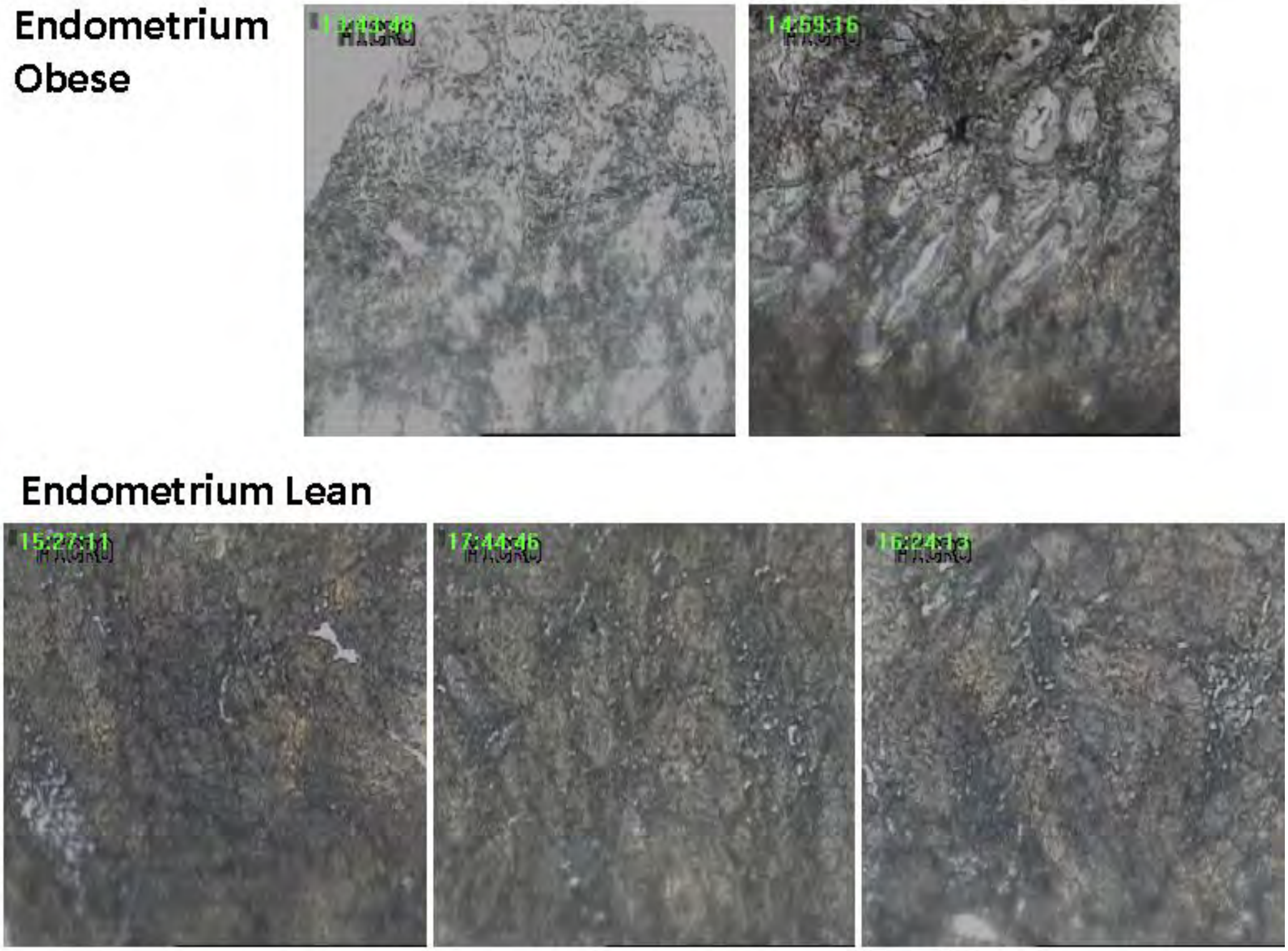
Endometrium
Obese
14:59:16
Endometrium Lean
15:27:11

Supplementary Fig. 45. Bright field imaging from TOF-SIMS showing tissue regions in human endometrium tissues.

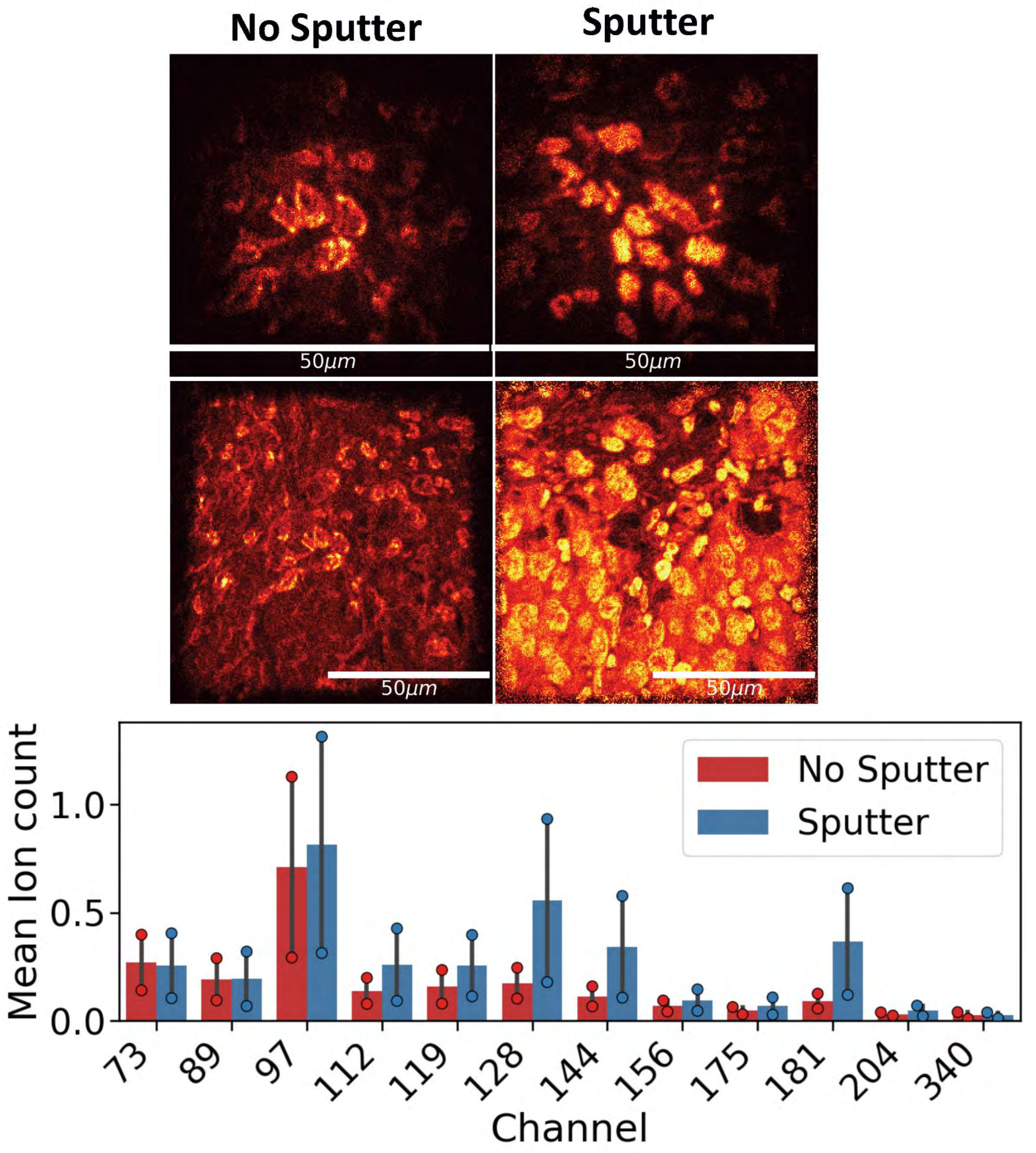
No Sputter
Sputter
50μm
50μm
50μm
50μm
Mean Ion count
0.0
0.5
1.0
No Sputter
Sputter
73
89
97
112
119
128
144
156
175
181
204
340
Channel

Supplementary Fig. 46. Comparison of TOF–SIMS imaging with and without sputtering. Bar plot showing the mean ion count when using a sputtering source for depth profiling (n = 2 samples). Scale bar 50 µm.

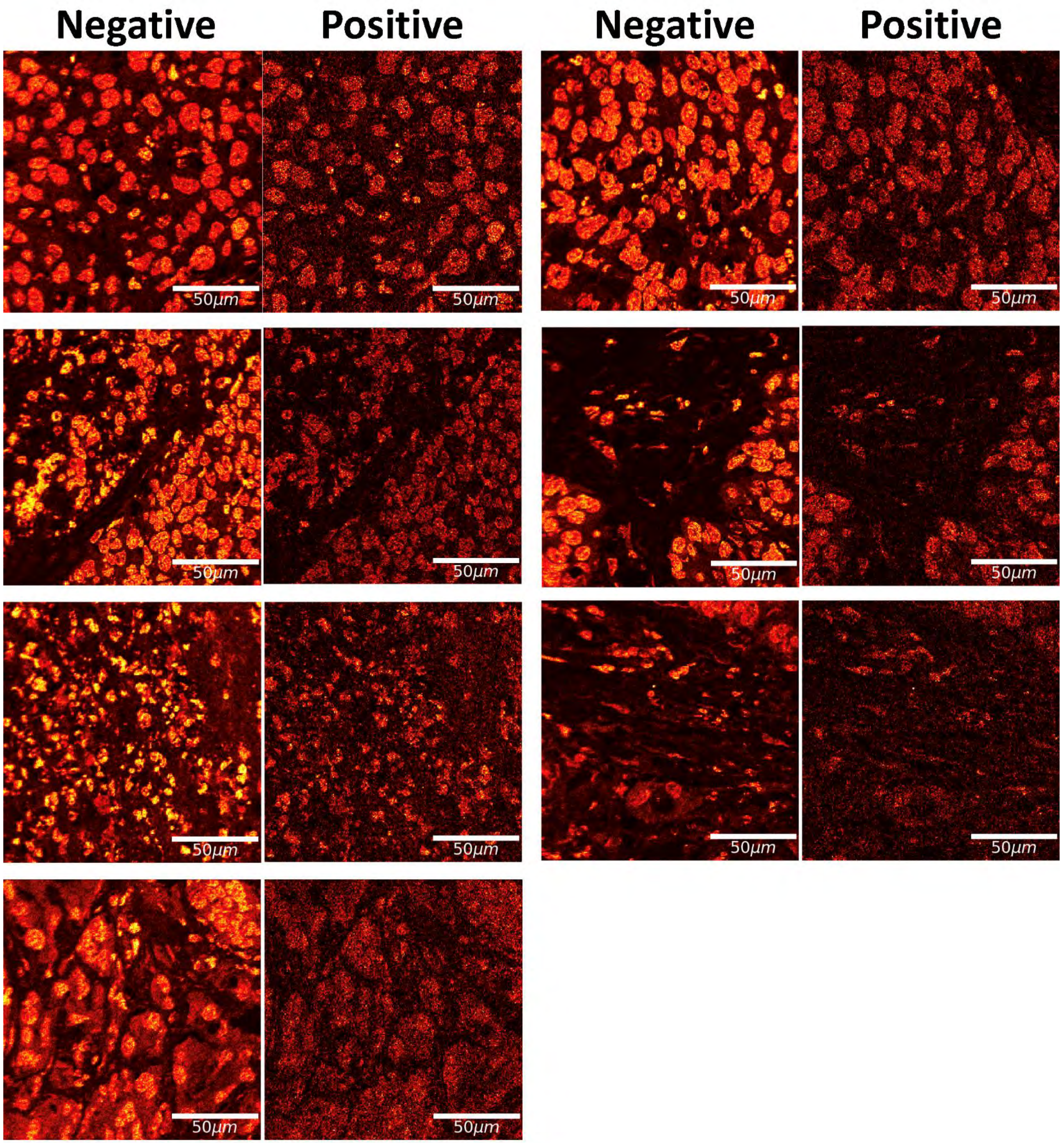
Negative
Positive
Negative
Positive
50μm
50μm
50μm
50μm
50μm
50μm
50μm
50μm
50μm
50μm
50μm
50μm
50μm
50μm

Supplementary Fig. 47. Comparison of TOF–SIMS imaging in positive and negative mode. Scale bar 50 μm.

LUNG CANCER

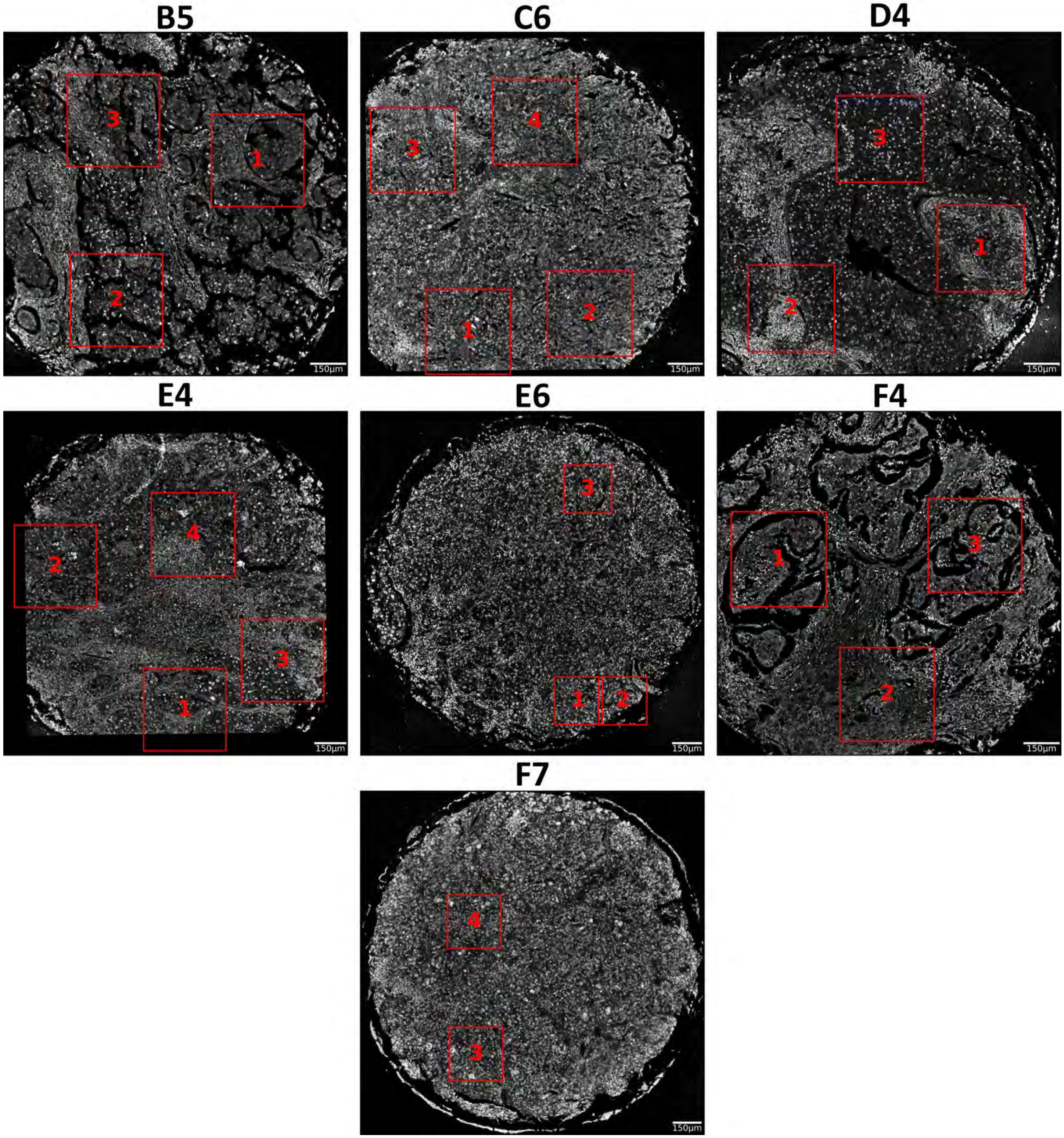

Supplementary Fig. 48. TOF–SIMS imaging regions highlighted in the IMC Histone H3 marker images in human lung cancer tissues. Scale bar 150 μm.

TONSIL DONOR A

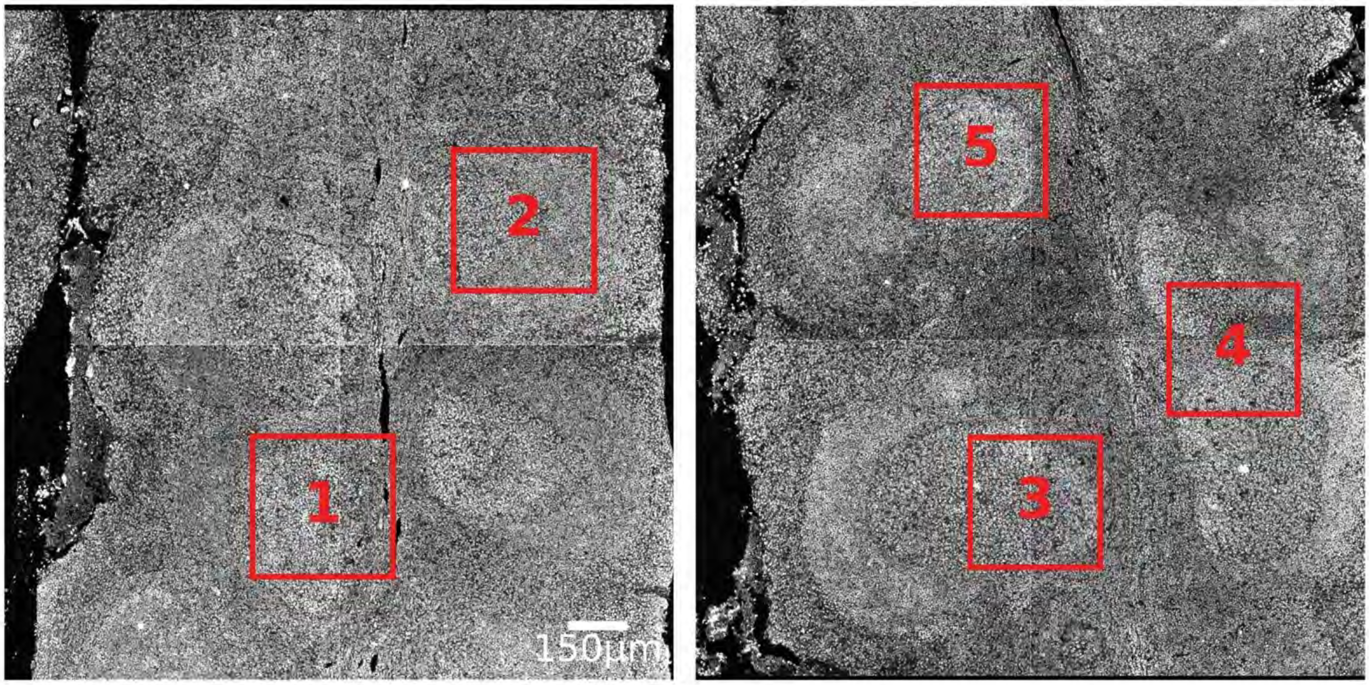

TONSIL DONOR E

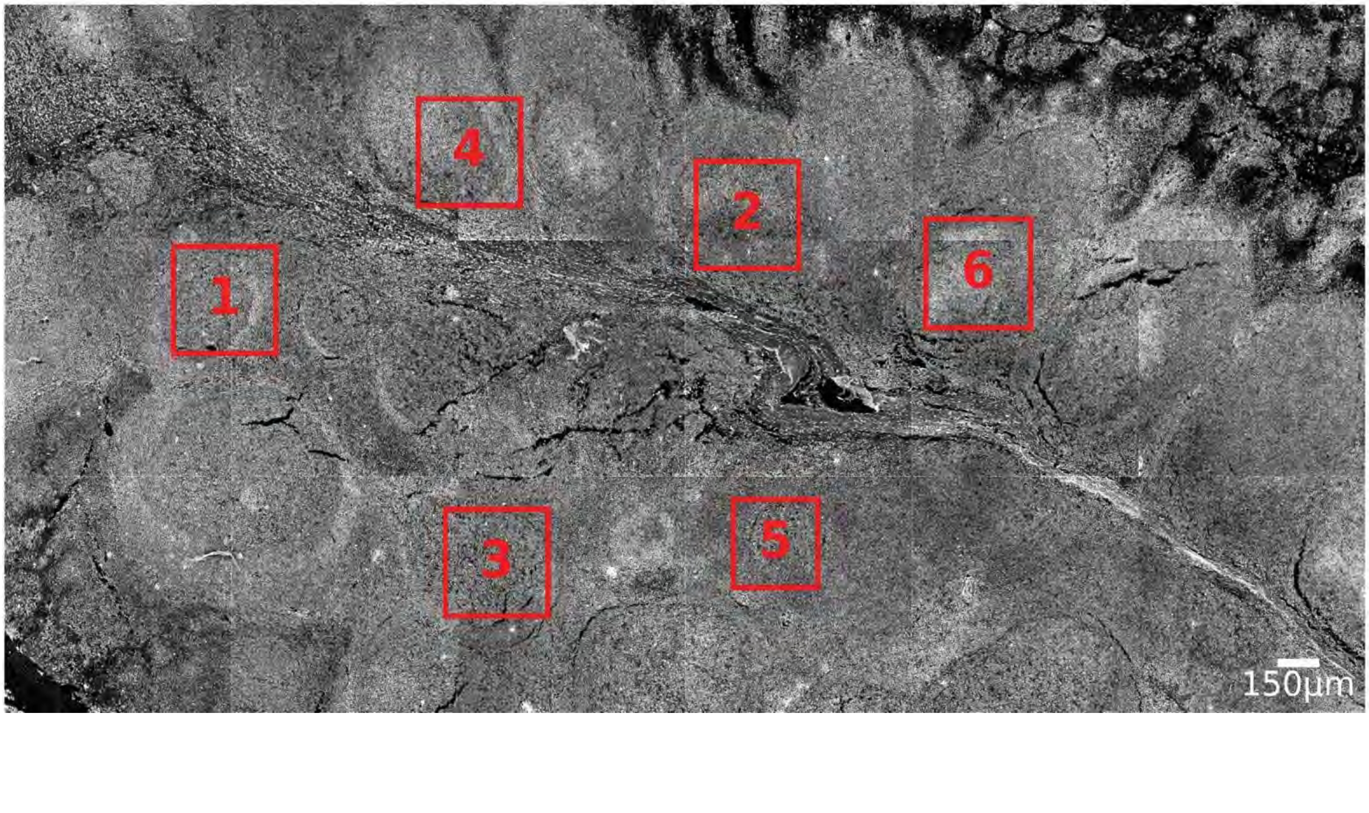

Supplementary Fig. 49. TOF–SIMS imaging regions showed in the IMC Intercalator marker images in human tonsil tissues. Scale bar 150 µm.

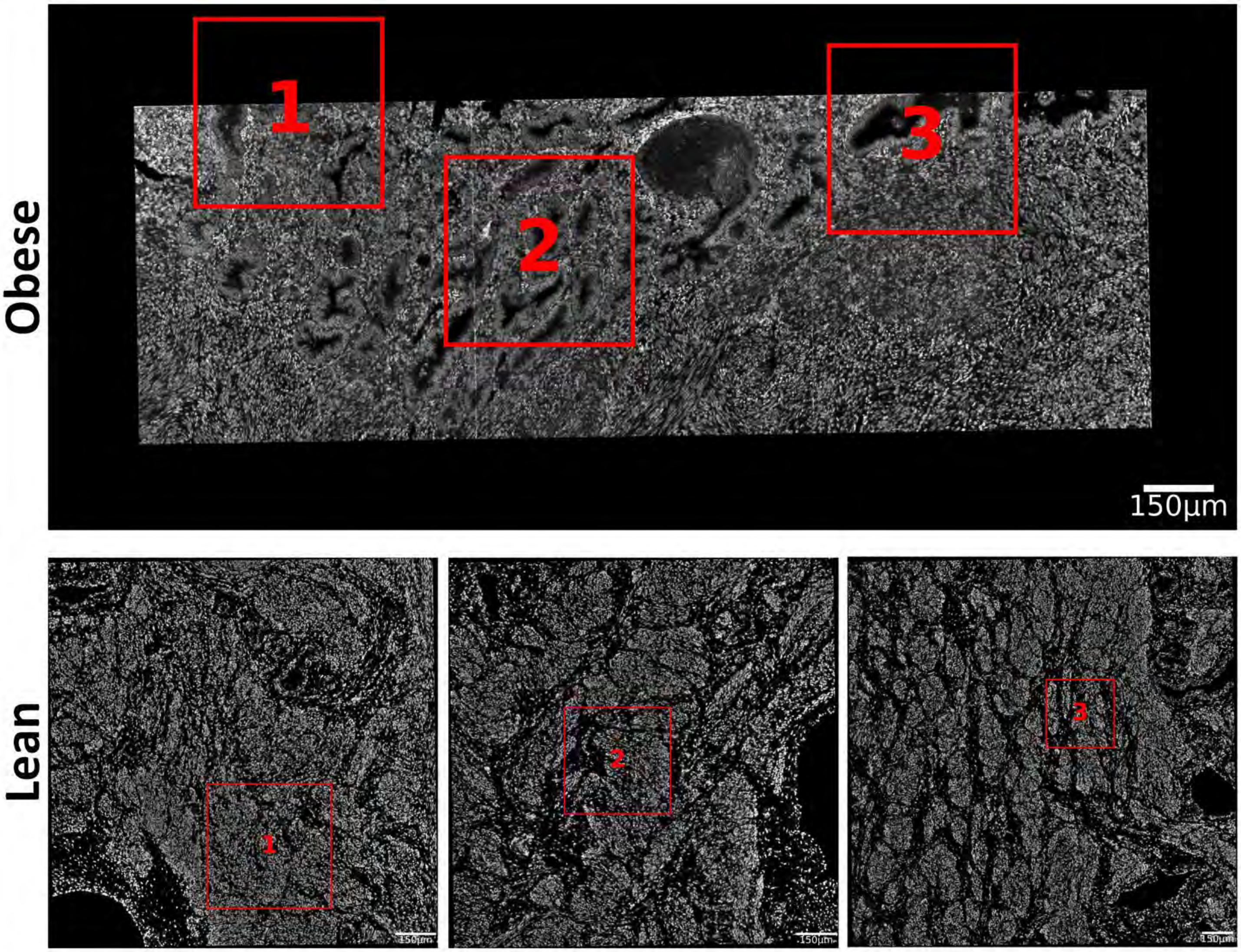

Obese
1
2
3
150µm
Lean
1
2
3

Supplementary Fig. 50. TOF–SIMS imaging regions showed in the IMC Intercalator marker images in human endometrium tissues. Scale bar 150 μm.

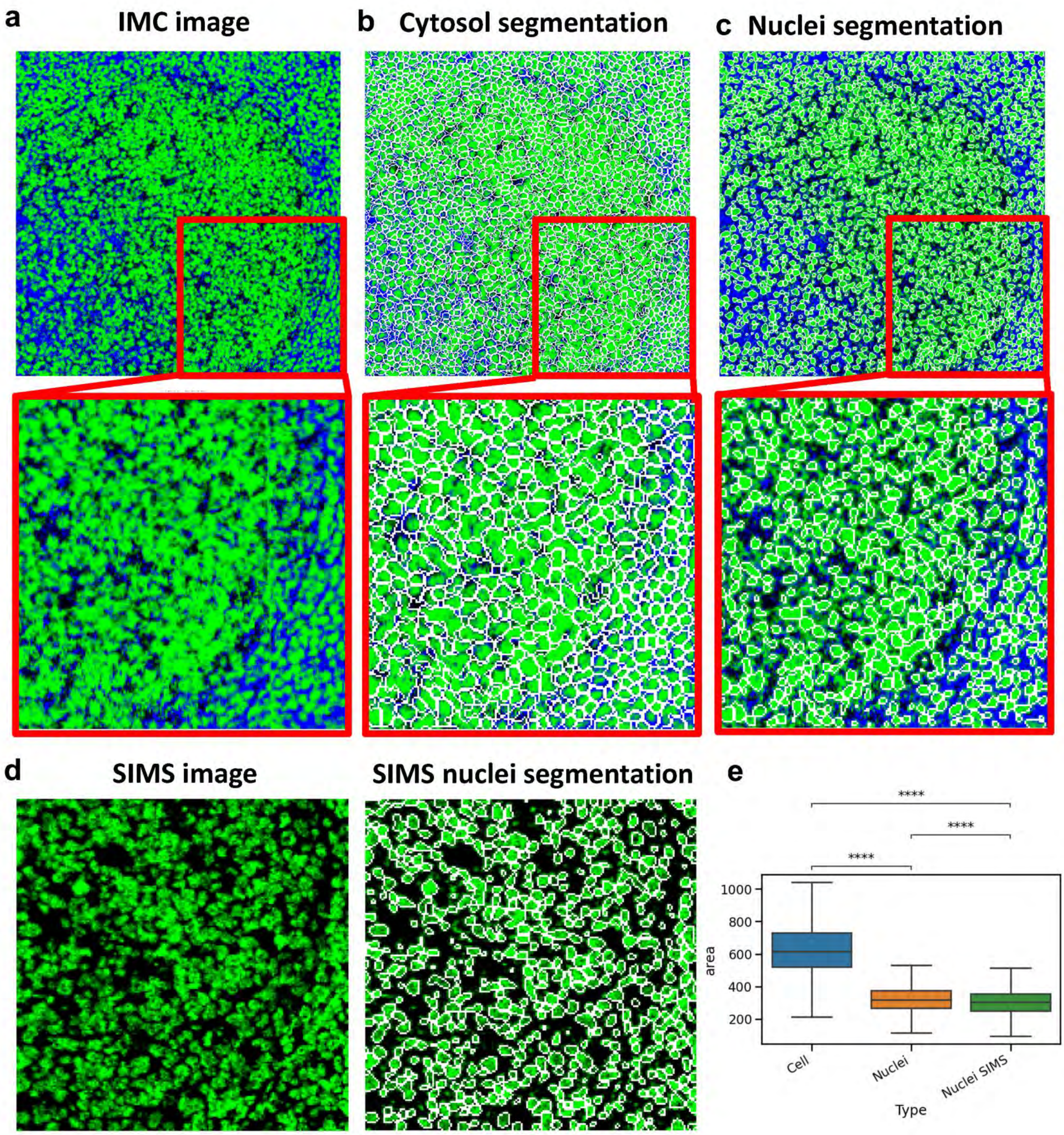
a IMC image
b Cytosol segmentation
c Nuclei segmentation
d SIMS image
SIMS nuclei segmentation
e
****
****
****
1000
800
600
400
200
area
Cell
Nuclei
Nuclei SIMS
Type

Supplementary Fig. 51. Comparison of single-cell segmentation in tonsil tissue. The image of cytosolic (blue) and nuclei (green) channels is shown by combining multiplex IMC markers **a** with the corresponding cytosol segmentation **b**, nuclei segmentation **c**, and SIMS image segmentation **d**. **e** Comparison of the average segmented cell areas for cell cytosol and nuclei region as well as nuclei region using the SIMS modality (n = 1530 cells). Mann-Whitney-Wilcoxon test was two-sided with Bonferroni correction (ns: $0.05 < p$, ****: $p<=0.0001$). All box plots with center lines showing the medians, boxes indicating the interquartile range, and whiskers indicating a maximum of 1.5 times the interquartile range beyond the box.

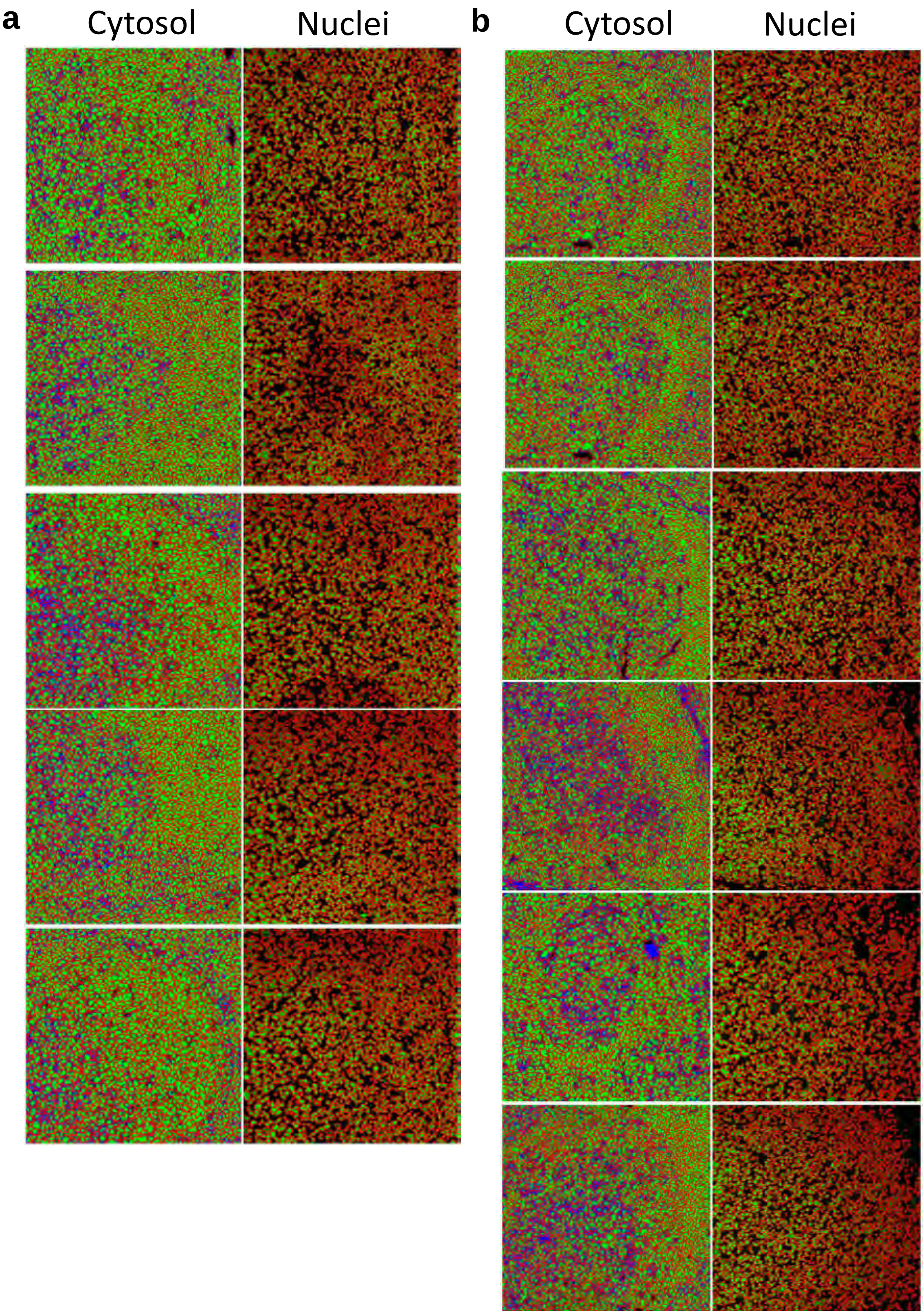
a
Cytosol
Nuclei
b
Cytosol
Nuclei

Supplementary Fig. 52. Comparison of single-cell segmentation in tonsil tissue donor A **a** and donor E **b** with *DeepCell* algorithm using cytosolic markers (left) and corresponding segmentation from nuclei channels in SIMS image (right).

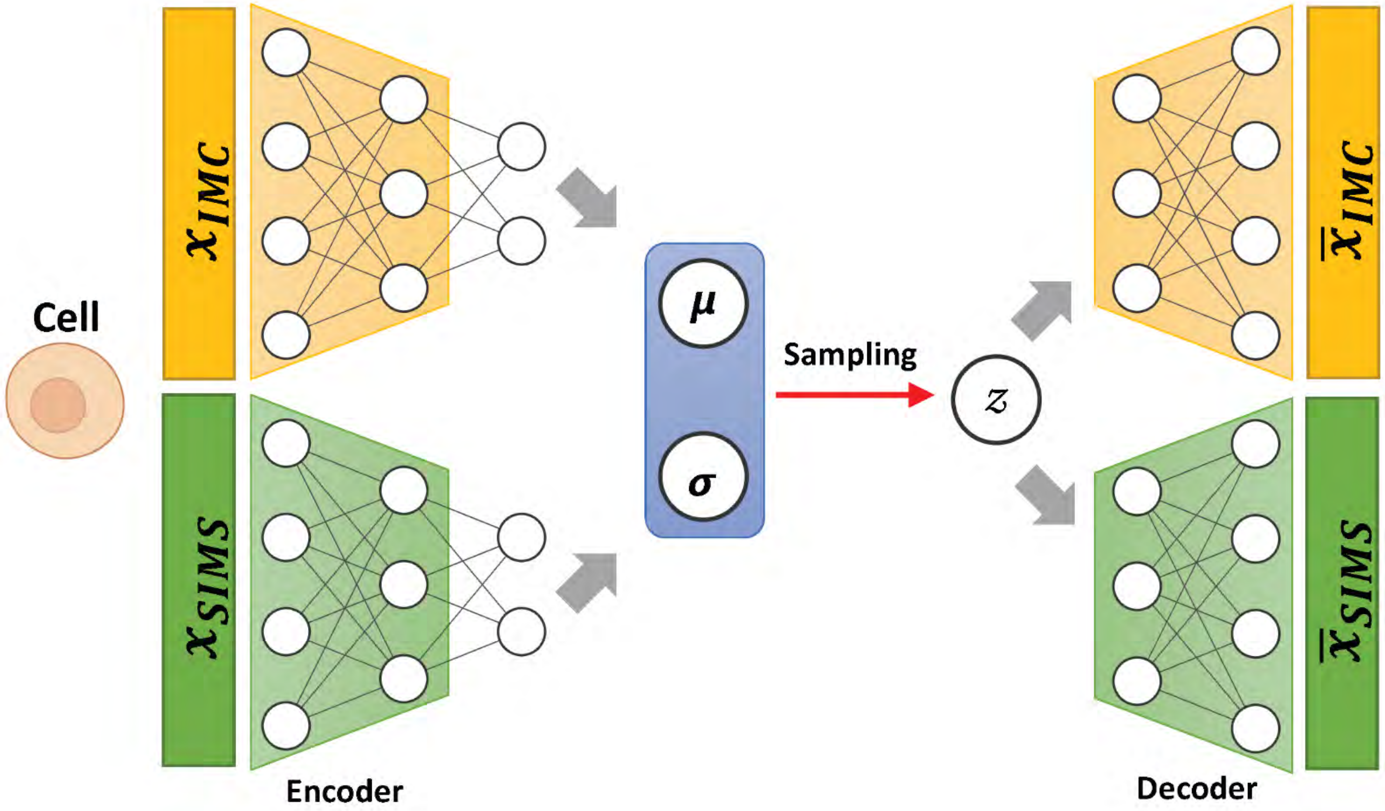

Cell
x_{IMC}
x_{SIMS}
Encoder
μ
σ
Sampling
z
$\overline{x}_{IMC}$
$\overline{x}_{SIMS}$
Decoder

Supplementary Fig. 53. VAE architecture for metabolite protein joint embedding. The input of the network is the metabolite and protein profiles and the output is the reconstructed profiles.

a

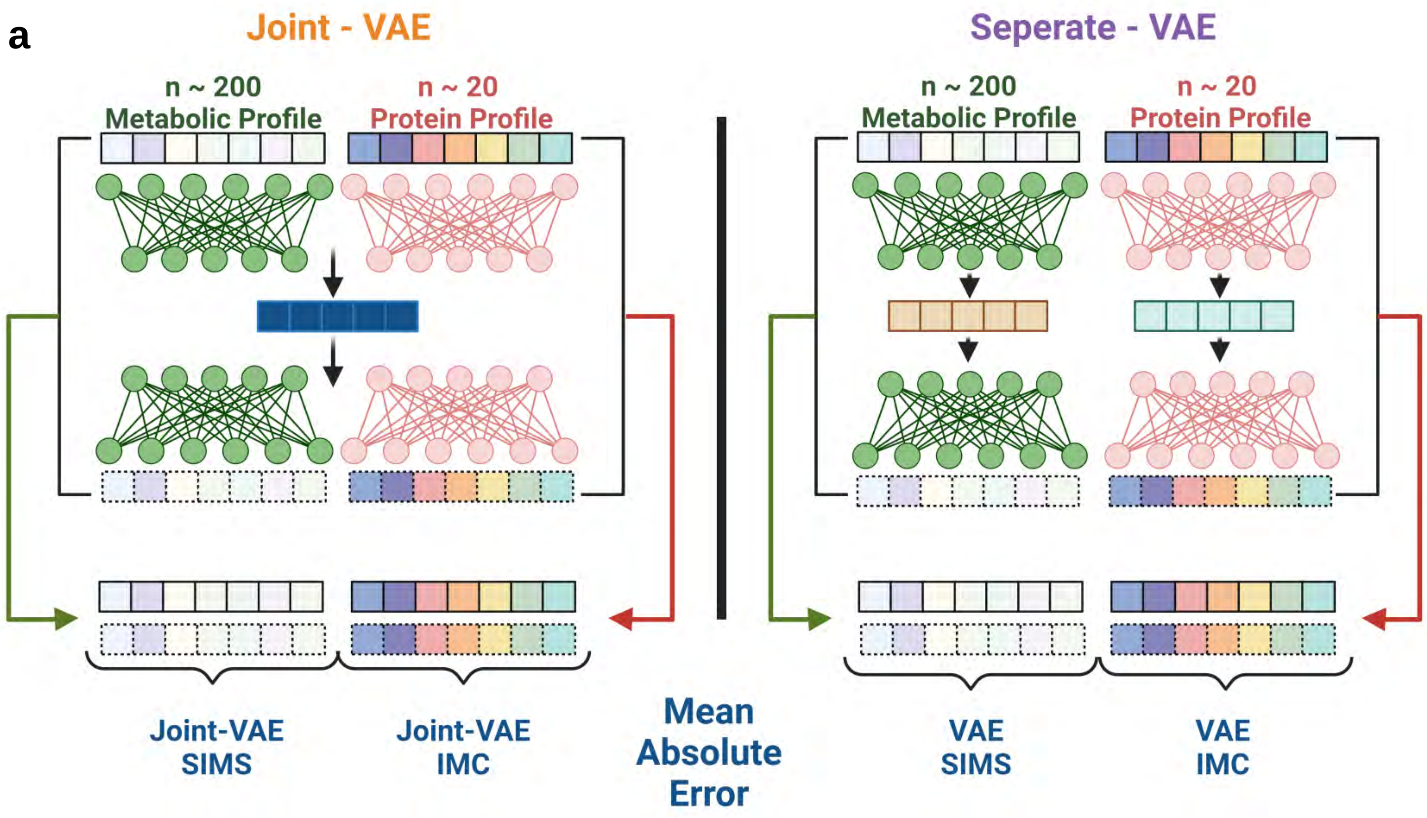

b

Dataset	Model	Modality	MAE
Tonsil	Joint-VAE	SIMS	0.6103
		IMC	0.0701
	VAE	SIMS only	0.6381
		IMC only	0.0813
Lung	Joint-VAE	SIMS	0.5074
		IMC	0.0822
	VAE	SIMS only	0.5589
		IMC only	0.0802
Endomet rium	Joint-VAE	SIMS	0.6201
		IMC	0.1093
	VAE	SIMS only	0.6307
		IMC only	0.1019

c

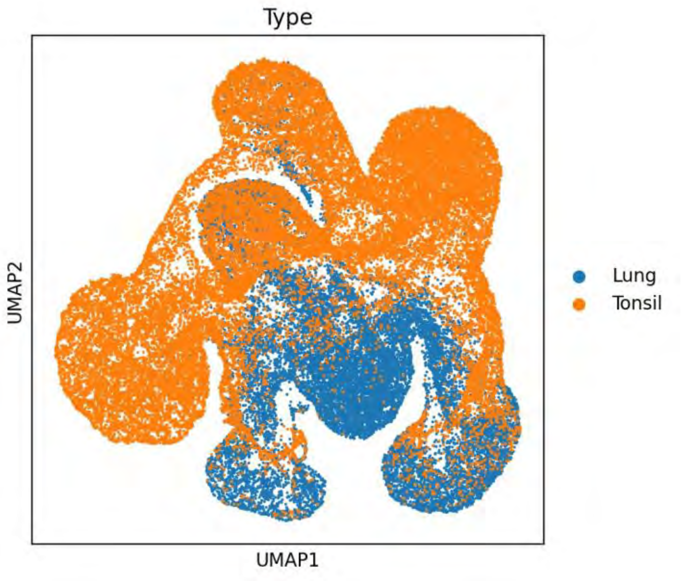

Supplementary Fig. 54. VAE embeddings for joint metabolite and protein profiles

a Schematic representation of joint embedding reconstruction compared to individual modality reconstruction using VAE for metabolite and protein profiles. Created with Biorender.com

b Table showing the mean absolute error of reconstruction from joint-VAE and single modality VAE across the imaged datasets.

c UMAP shows the VAE joint embedding using common protein markers in lung cancer and tonsil datasets.

Supplementary Tables

Method	Capability	Mass imaging	Resolution	Cell type specificity	Cell target region	Cell size comptability
**scSpaMet**	Integrates metabolite and specific protein labeling at single-cell level	TOF-SIMS/IMC	500-nm resolution	25 Cell types	Cytosol and nucleus	Small cell size
**3D-SMF**	Detect tissue region metabolites	TOF-SIMS	500-nm resolution	20 protein markers	Tissue region	None
**SpaceM**	Integrates MALDI with confocal imaging	MALDI	10-μm resolution	2 Cell types	Whole-cell	Large cell size
**SEAM**	Only detect nuclear metabolites	TOF-SIMS	1-μm resolution	No true cell types	Nucleus	Small cell size
**Raman Spectrometry**	Only detects limited metabolic targets	Microscopy	300-nm resolution	No true cell types	Cytosol and nucleus	Small cell size

Supplementary Table 1. Comparison of emerging single-cell spatial metabolomics technologies

Method	Integration modality	Require matching markers	Same cell measurements	Same tissue
MARIO	Protein and transcriptome measurements. (CODEX / CITE-seq)	Partially shared features/markers	No	No
MNNs, Scanorama, Conos	The same modality designed for scRNA-seq batch effect removal	Total matching	No	No
Seurat v4	Protein, ATAC, and RNA	Partial matching cell type	Yes	Yes
Liger	DNA methylation, chromatin accessibility, and RNA	Partial matching cell type	No	No
SpaceM	No integration	No matching. Define cell type using a light microscope	Yes	Yes
SEAM	Metabolites and Transcriptome	No matching. Analysis of correlative expression from sequential tissues	No	No

Supplementary Table 2. Comparison of emerging single-cell spatial multi-omic analysis

Marker	Clone	Metal Tag	Dilution
CD20	H1	161Dy	1:400
CD3	Polyclonal	170Er	1:100
CD4	ERP6855	156Gd	1:200
CD45RO	UCHL1	173Yb	1:50
CD68	KP1	159Tb	1:50
CD8a	C8/144B	162Dy	1:100
FoxP3	236A/E7	155Gd	1:30
Pan-Keratin	C11	148Nd	1:100
Granzyme B	EPR20129-17	167Er	1:100
Ki-67	B56	168Er	1:50
SMA	1A4	141Pr	1:200
Collagen type I	Polyclonal	169Tm	1:300
E-cadherin	24E10	158Gd	1:50
Histone 3	D1H2	171Yb	1:50
Vimentin	D21H3	143Nd	1:100
CD11b	EMP1344	149Sm	1:50
CD44	IM7	153Eu	1:100
CD31	EPR3094	151Eu	1:100
CD45RA	HI100	166Er	1:50
CD11c	Polyclonal	154Sm	1:100
Intercalator	—	191Ir/193Ir	1:400

Supplementary Table 3. List of antibodies, their conjugated metal tags, their clones, and their concentrations used for imaging mass cytometry in lung cancer microarrays.

**Core number**	**Sex**	**Pathology Diagnosis**	**Grade**	**Stage**	**Type**
B5, F7	M	Adenocarcinoma	3	IIIA	Malignant
C6, F4	M	Adenocarcinoma	3	IIA	Malignant
D4, E4, E6	M	Adenocarcinoma	3	IB	Malignant

Supplementary Table 4. BS04081a patient specifications for cancer type, stage, and grade.

Donor No.	Sex	Pathology Diagnosis
A-E	M	Normal Tonsil Tissue

Supplementary Table 5. HuFPT161 patient specifications for tonsil tissue.

Marker	Clone	Metal Tag	Dilution
CD3	Polyclonal	170Er	1:100
CD45RO		166Er	1:50
CD45RA	UCHL1	173Yb	1:50
CD8a	C8/144B	!62Dy	1:100
Pan-Keratin	C11	148Nd	1:100
SMA	1A4	141Pr	1:200
E-Cadherin	24E10	158Gd	1:50
Vimentin	D21H3	143Nd	1:100
Intercaltor		192/193Ir	1:100

Supplementary Table 6. List of antibodies, their conjugated metal tags, their clones, and their concentrations used for imaging mass cytometry in endometrium tissues

Supplementary References

1. Passarelli, M. K. *et al.* The 3D OrbiSIMS—label-free metabolic imaging with subcellular lateral resolution and high mass-resolving power. *Nat. Methods* **14**, 1175–1183 (2017).
2. Yuan, Z. *et al.* SEAM is a spatial single nuclear metabolomics method for dissecting tissue microenvironment. *Nat. Methods* **18**, 1223–1232 (2021).
3. Piehowski, P. D. *et al.* MS/MS Methodology To Improve Subcellular Mapping of Cholesterol Using TOF-SIMS. *Anal. Chem.* **80**, 8662–8667 (2008).
4. Kezutyte, T., Desbenoit, N., Brunelle, A. & Briedis, V. Studying the penetration of fatty acids into human skin by ex vivo TOF-SIMS imaging. *Biointerphases* **8**, 3 (2013).
5. Passarelli, M. K. & Winograd, N. Lipid imaging with time-of-flight secondary ion mass spectrometry (ToF-SIMS). *Biochim. Biophys. Acta BBA - Mol. Cell Biol. Lipids* **1811**, 976–990 (2011).
6. Ganesh, S. *et al.* Spatially resolved 3D metabolomic profiling in tissues. *Sci. Adv.* **7**, (2021).
7. Bernard, L., Crockett, R. & Kawecki, M. Monosaccharides: A ToF-SIMS reference spectra database. I. Negative polarity. *Surf. Sci. Spectra* **26**, 025001 (2019).
