# Supplementary material for: Single-cell spatial metabolomics with cell-type specific protein profiling for tissue systems biology
Source: Nat Commun. 2023 Dec 13;14:8260. doi: 10.1038/s41467-023-43917-5 (PMC10716522; doi:10.1038/s41467-023-43917-5)
Supplement: Supplementary file 3 — Description of Additional Supplementary Files [file 41467_2023_43917_MOESM3_ESM.pdf]

Description of Additional Supplementary Files

Supplementary Data 1: List of antibodies, their conjugated metal tags, their clones, and their concentrations used for the imaging mass cytometry tissue labeling step. * refers to the markers/antibodies with in-house conjugations to the metal tags.

Supplementary Data 2: Putative annotation of TOF-SIMS channels sorted by m/z.

Supplementary Data 3: Putative annotation of TOF-SIMS channels sorted by literature reference.
