# Supplementary material for: Single-cell spatial metabolomics with cell-type specific protein profiling for tissue systems biology
Source: Nat Commun. 2023 Dec 13;14:8260. doi: 10.1038/s41467-023-43917-5 (PMC10716522; doi:10.1038/s41467-023-43917-5)
Supplement: Supplementary file 7 — Reporting Summary [file 41467_2023_43917_MOESM7_ESM.pdf]

nature portfolio

Corresponding author(s): Ahmet F Coskun

Last updated by author(s): Nov 9, 2023

Reporting Summary

Nature Portfolio wishes to improve the reproducibility of the work that we publish. This form provides structure for consistency and transparency in reporting. For further information on Nature Portfolio policies, see our Editorial Policies and the Editorial Policy Checklist.

Statistics

For all statistical analyses, confirm that the following items are present in the figure legend, table legend, main text, or Methods section.

n/a	Confirmed	
☐	☒	The exact sample size (*n*) for each experimental group/condition, given as a discrete number and unit of measurement
☐	☒	A statement on whether measurements were taken from distinct samples or whether the same sample was measured repeatedly
☐	☒	The statistical test(s) used AND whether they are one- or two-sided<br>*Only common tests should be described solely by name; describe more complex techniques in the Methods section.*
☐	☒	A description of all covariates tested
☐	☒	A description of any assumptions or corrections, such as tests of normality and adjustment for multiple comparisons
☐	☒	A full description of the statistical parameters including central tendency (e.g. means) or other basic estimates (e.g. regression coefficient) AND variation (e.g. standard deviation) or associated estimates of uncertainty (e.g. confidence intervals)
☐	☒	For null hypothesis testing, the test statistic (e.g. *F*, *t*, *r*) with confidence intervals, effect sizes, degrees of freedom and *P* value noted<br>*Give P values as exact values whenever suitable.*
☒	☐	For Bayesian analysis, information on the choice of priors and Markov chain Monte Carlo settings
☐	☒	For hierarchical and complex designs, identification of the appropriate level for tests and full reporting of outcomes
☐	☒	Estimates of effect sizes (e.g. Cohen's *d*, Pearson's *r*), indicating how they were calculated

Our web collection on statistics for biologists contains articles on many of the points above.

Software and code

Policy information about availability of computer code

Data collection	TOF-SIMS (IONTOF 5 GmbH, Münster, Germany), Imaging Mass Cytometry, Fluidigm's MCD Viewer software (v 1.0.560.2), Keyence Microscope software units commercially available
Data analysis	Custom Python Codes. The analysis codes are available at https://github.com/coskunlab/ScSpaMet and also provided as a supplementary material. The IMC data was exported using MCD Viewer (v.1.0.560.2). Analysis used Anaconda (v. 4.12.0) and Jupyterlab (v. 3.2.8).

For manuscripts utilizing custom algorithms or software that are central to the research but not yet described in published literature, software must be made available to editors and reviewers. We strongly encourage code deposition in a community repository (e.g. GitHub). See the Nature Portfolio guidelines for submitting code & software for further information.

Data

Policy information about availability of data

All manuscripts must include a data availability statement. This statement should provide the following information, where applicable:

- Accession codes, unique identifiers, or web links for publicly available datasets
- A description of any restrictions on data availability
- For clinical datasets or third party data, please ensure that the statement adheres to our policy

Data availability
The IMC and 3D-SMF image data generated in this study have been deposited at https://doi.org/10.5281/zenodo.6784251. The mass spectrometry proteomics data

have been deposited to the ProteomeXchange Consortium via the PRIDE partner repository with the dataset identifier PXD045840. The metabolomic raw data have been deposited to MetaboLights with the dataset identifier MTBLS8685 (www.ebi.ac.uk/metabolights/MTBLS8685). Source Data are provided with this paper.

Research involving human participants, their data, or biological material

Policy information about studies with human participants or human data. See also policy information about sex, gender (identity/presentation), and sexual orientation and race, ethnicity and racism.

Reporting on sex and gender	Sex was not considered in the study design. Sex of participants was determined based on self-report. No sex-based analyses was performed due to lack of female patient in tissue cores.
Reporting on race, ethnicity, or other socially relevant groupings	Race, Ethnicity, or other socially relevant groupings were not considered in the study design.
Population characteristics	For lung tissue samples and tonsil samples, covariate-relevant population characteristics is provided in Supplementary Table 4-5.
Recruitment	*Describe how participants were recruited. Outline any potential self-selection bias or other biases that may be present and how these are likely to impact results.*
Ethics oversight	*Identify the organization(s) that approved the study protocol.*

Note that full information on the approval of the study protocol must also be provided in the manuscript.

Field-specific reporting

Please select the one below that is the best fit for your research. If you are not sure, read the appropriate sections before making your selection.

[x] Life sciences [ ] Behavioural & social sciences [ ] Ecological, evolutionary & environmental sciences

For a reference copy of the document with all sections, see nature.com/documents/nr-reporting-summary-flat.pdf

Life sciences study design

All studies must disclose on these points even when the disclosure is negative.

Sample size	21 regions from tumor microarray, 11 regions from tonsil slides, and 5 regions from large endometrium slides.<br>No Statistical method was used to predetermine sample size.<br>Samples were collected until the sample size were sufficient to give comparison and reliable estimates.
Data exclusions	There are no data exclusions.
Replication	At least 2 replicated per tissue from different imaging regions.
Randomization	Not randomized. Covariates were not analyzed in our study. We are interested in spatial correlation of proteomic and metabolomic.
Blinding	Not blinded. We are interested in spatial correlation of proteomic and metabolomic at the single cell level.

Reporting for specific materials, systems and methods

We require information from authors about some types of materials, experimental systems and methods used in many studies. Here, indicate whether each material, system or method listed is relevant to your study. If you are not sure if a list item applies to your research, read the appropriate section before selecting a response.

Materials & experimental systems

n/a	Involved in the study	
[ ]	[x]	Antibodies
[x]	[ ]	Eukaryotic cell lines
[x]	[ ]	Palaeontology and archaeology
[x]	[ ]	Animals and other organisms
[x]	[ ]	Clinical data
[x]	[ ]	Dual use research of concern
[x]	[ ]	Plants

Methods

n/a	Involved in the study	
[x]	[ ]	ChIP-seq
[x]	[ ]	Flow cytometry
[x]	[ ]	MRI-based neuroimaging

Antibodies

Antibodies used	Supplementary Table 2,3 and 4 shows the details of these antibodies CD20, CD3, CD4, CD45RO, CD68, CD8a, FoxP3, Pan-Keratin, Granzyme B, Ki-67, SMA, Collagen Ty1, E-cadherin, Histone 3, Vimentin, CD11b, CD44, CD31, CD45RA, CD11C, Intercaltor, CD38, BCL6, ICOS1, C-Myc, PD1, CD27, CD138, CXCR5, EZH2, CD21, CD86, CD83, CXCR4, H3K27ME3.<br>Antibodies were obtained from Standards Biotools:<br>EPR4106, D21H3, K112-91, D1K2T, EPR1344, Polyclonal, 236A/E7, ERP6855, H1, C8/144B, 9E10, ERP4877(2), B56, Poly, EPR8569<br>Antibodies were obtained from abcam:<br>EPR6454, EPR23463-30, EPR9307(2), EP3093, BU63, EPR23809-19, UMB2,<br>Antibodies were obtained from Cell Signaling technologies:<br>C36B11
Validation	Antibodies were mostly obtained from Fluidigm as a validated immune panel. Additional validations were performed by immunohistochemistry (IHC) in tonsil formalin-fixed, paraffin-embedded (FFPE) sections. All antibodies were validated on human samples before and after metal isotope conjugation

Plants

Seed stocks	N/A
Novel plant genotypes	N/A
Authentication	N/A
